# Deaminative methylselenation of anlines with PhSO_2_SeCH_3_

**DOI:** 10.3389/fchem.2026.1762521

**Published:** 2026-03-31

**Authors:** Limei Wang, Bo Zhang, Yan Yan, Lijuan Liu, Ge Ou, Ning Xiao

**Affiliations:** 1 Pharmaceutical Management Department, Jilin FAW General Hospital, Changchun, China; 2 Medical Equipment Department, Jilin FAW General Hospital, Changchun, China; 3 Department of Laboratory Medicine, Jilin FAW General Hospital, Changchun, China; 4 Neurosurgery Department, Jilin FAW General Hospital, Changchun, China; 5 Office of the Drug Clinical Trial Institution, Jilin FAW General Hospital, Changchun, China

**Keywords:** acid-free, anilines, Deamination, metal-free, methylselenation

## Abstract

Herein, we present a method for methylselenation of aryldiazonium salts that does not require metals or acids, achieved by directly transforming an amino group into a methylseleno group. This strategy is compatible with diverse anilines and facilitates rapid late-stage methylselenation of key pharmaceuticals and their derivatives, clearly demonstrating the utility of PhSO_2_SeCH_3_ as a methylselenation reagent. Mild reaction conditions proceed efficiently at a 100 mmol scale, allowing double methylselenation of anilines and concise construction of aryl-SeCD_3_.

## Introduction

**SCHEME 1:**
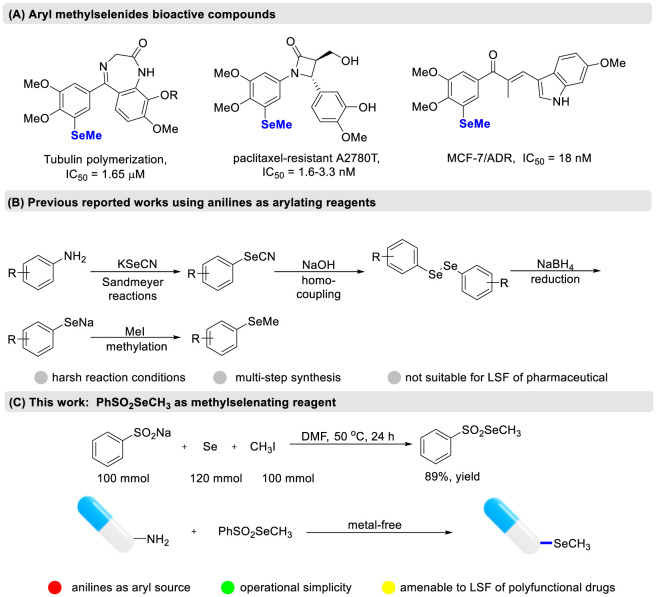
Strategies for the construction of aryl methyl selenides. **(A)** Aryl methylselenides bioactive compounds. **(B)** Previous reported works using anilines as arylating reagents. **(C)** This work: PhSO_2_SeCH_3_ as methylselenating reagent.

Owing to the distinctive biological properties of selenide compounds, the incorporation of selenium atom into molecular skeleton has attracted ongoing interest in medicinal chemistry (Hou et al., 2022; Madabeni et al., 2024). These compounds could enhance absorption by cancer cells and possess strong antioxidant effects (Chuai et al., 2021). Among the commonly recognized selenides, aryl methyl selenides are frequently encountered in numerous candidate drugs (Scheme 1A) (An et al., 2018; Tang et al., 2021). The atomic substitution strategy is often employed in the structural optimization of lead compounds to discover new drugs. Schiesser group replaced the thiomethyl group in fosinopril with a selenomethyl group, which effectively inhibits the angiotensin AT1 receptor subtype and extends duration of blood pressure-lowering effect (Lei et al., 2023). Despite the potential of aryl methyl selenides in drug design, there are only a few synthetic methods available for introducing the methylselenyl group into an aryl ring. A traditional approach involves nucleophilic substitution between methyl iodide and sodium benzeneselenolate (Scheme 1B) (Levanova et al., 2013). However, due to the instability of sodium benzeneselenolate, these reactions must be performed under strictly anhydrous conditions.

Structurally diverse anilines are commercially available starting material. The amino group can be transformed into various significant functional groups through the Sandmeyer reaction. Existing methods for converting anilines into aryl methyl selenides generally require multiple steps, including Sandmeyer selenocyanation, followed by homo-coupling, reduction and subsequent methylation (Scheme 1B) (Pang et al., 2017). Although this four-step process is relatively reliable and widely used in pharmaceutical chemistry, the multiple isolation steps often result in low yields of the final products. Additionally, this method typically involves isolating diazonium salts and require acid additives to facilitate their formation. Moreover, the continuous use of acidic, basic, and reductive reaction conditions inevitably results in poor functional group tolerance, environmental concerns and limited product diversity. Therefore, a direct methylselenation of anilines to access valuable aryl methyl selenides is highly fascinating. As far as we know, such a transformation has not yet been described. Moreover, Liang group reported an example of manganese-assissted direct methylation of diphenyl diselenides using dimethyl carbonate as the methyl source (Zhang et al., 2023). Nevertheless, the use of excess metal reducing agents generates a significant amount of solid waste during large-scale reactions, posing serious environmental burden. Dimethyl diselenide is also frequently employed as a methylselenating reagent with arylboronic acids or aryl carboxylic acids under appropriate conditions (Chen et al., 2022; Fu et al., 2022; Wang et al., 2017; Zhou et al., 2025). However, the involvement of transition metal catalysts often results in residual metal ions remaining in the final products. A key drawback of electrochemical synthesis is the challenge of scaling up the reaction. Moreover, dimethyl diselenide has a very strong odor, and even minor leaks can contaminate an entire laboratory, greatly limiting its industrial use. These difficulties in synthesizing aryl methyl selenides considerably impede their design and discovery in the development of new drugs. We report the first example of a metal- and acid-free deaminative methylselenation of anilines using PhSO_2_SeCH_3_ (Scheme 1C). The mild and practical reaction conditions allow the transformation of various anilines into functionalized aryl methyl selenides with excellent tolerance for different functional groups.

## Results and discussion

Initially, we performed methylselenation of anilines with the addition of an acidic additive (Table 1), as acid is conventionally used to promote aniline diazotization. Disappointingly, no desired product was observed, regardless of whether metal catalysts were added or organic solvents were replaced under different atmospheric conditions, except for the occasional observation of deaminative protonation byproducts (entry 1). Later, a control experiment demonstrated that the addition of tBuONO and acid decomposed PhSO_2_SeCH_3_ (Qiu et al., 2013; Zheng et al., 2014), whereas only the addition of tBuONO in DMA at room temperature under air remarkably enhanced the yield of **3a** (entry 2). Next, we studied common additives to optimize the reactions, such as bases perhaps cleave the sulfonyl selenoester and transition-metal salts may accelerate denitrogenated radical cross-coupling reactions (Huang et al., 2020; Matheis et al., 2014). To our surprise, different copper salts, typically used in the Sandmeyer reaction, showed extremely poor catalytic activity (entry 3). However, the use of FeCl_2_ significantly enhanced the transformation (entry 4). To our delight, the yield of the target product remained almost unchanged without the addition of a catalyst. It is noteworthy that the utilize of Cs_2_CO_3_ completely inhibited the conversion of PhSO_2_SeCH_3,_ and most of the raw material was recovered (entry 5). Furthermore, the introduction of benzoyl peroxide into the reaction system still obtained the anticipated product, suggesting that the current methylselenation proceeds via a radical pathway (entry 6) (Xia et al., 2014). As far as we know, the diazotization reaction of aromatic amines is exothermic, which favors the stabilization of diazonium salts at low temperatures. Therefore, we decided to conduct the reaction at 0 °C (entry 7). Next, we examined the effect of solvent and found that other solvents resulted in lower yields of the corresponding product (entries 8-10). In addition, CH_3_CN and water with relatively poor hydrogen-donating capacities, also inhibited this transformation. When the reaction solution was placed under an oxygen atmosphere, the yield of the product decreased significantly (entry 11). Finally, we tested different nitration reagents to improve the yield and found that the best nitration agent was still tBuONO, while NaNO_2_ had no effect at all (entries 12-14). A blank experiment confirmed the importance of tBuONO in forming diazonium salts of aniline (entry 15).

**TABLE 1:** Reaction optimization^a^.

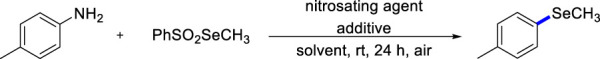				
Entry	Nitrosating agent	Additive [mol%]	Solvent	Yield of 3a [%]^b^
1	tBuONO	TsOH (100)	DMA	0
2	tBuONO	​	DMA	82
3	tBuONO	Cu(OAc)_2_ (20)	DMA	4
4	tBuONO	FeCl_2_ (20)	DMA	77
5	tBuONO	Cs_2_CO_3_ (100)	DMA	0
6	tBuONO	BPO	DMA	74
7^c^	tBuONO	​	DMA	86
8	tBuONO	​	THF	Trace
9	tBuONO	​	CH_3_CN	0
10	tBuONO	​	H_2_O	0
11^d^	tBuONO	​	DMA	49
12	TBANO_2_	​	DMA	33
13	iAmONO	​	DMA	65
14	NaNO_2_	​	DMA	0
15	​	​	DMA	0

^a^ Optimal conditions: p-toluidine (0.2 mmol), PhSO_2_SeCH_3_ (0.3 mmol), nitrosating agent (0.3 mmol), additive, solvent (2 mL), room temperature, in air, 4 h.

^b^ Isolated yield.

^c^ 0 °C.

^d^ Oxygen atmosphere.

**SCHEME 2:**
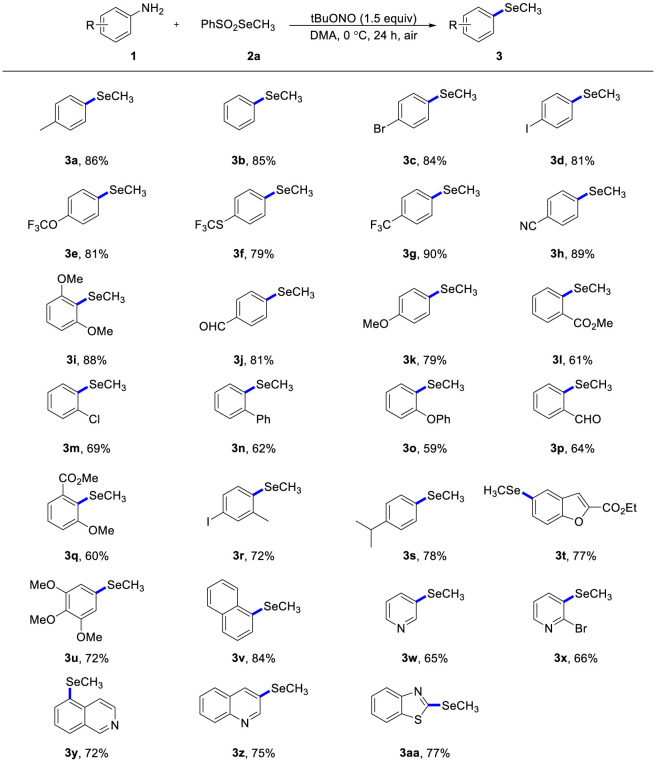
Scope of anilines^a^. a) Optimal conditions: anilines (0.2 mmol), PhSO2SeMe (0.3 mmol), tBuONO (0.3 mmol), DMA (2 mL), 0 °C, air, 24 h. b) Isolated yield.

With robust reaction conditions established, we evaluated the substrate scope with respect to various functionalized anilines (Scheme 2). In general, this methylselenation showed excellent tolerance for various functional groups, including alkyl (**3a**, **3r**, **3j**), halogen (**3c**, **3d**, **3m**, **3r**), trifluoromethoxy (**3e**), trifluoromethylthio (**3f**), trifluoromethyl (**3g**), cyano (**3h**), suIfonyl (**3i**), ester (**3l**, **3q**), formyl (**3p**) and methoxyl (**3u**). The steric hindrance had a negligible effect when ortho-substituted anilines were exposed to the ideal reaction conditions, and the intended products (**3l**-**3s**) were produced in moderate to good yields. Remarkably, anilines bearing iodine substitutent substituent was also compatible with this transformation, giving the correpsonding product (**3d**) in good yield. In addition, multifunctionalized anilines, such as 5-aminosalicylic acid was suitable for this chemo-selective methylselenation, affording product **3t**. Similar to aniline, 1-naphthylamine was compatible with the reaction conditions, providing the desired product **3v** in excellent yield. Trisubstituted anilines (**3u**) was toleranced, producing compounds containing the important 3,4,5-trimethoxy in numerous antitumor active molecules. Heterocyclic compounds are prevalent in many pharmaceutical agents, selective methylselenation of these frameworks offers a valuable platform for drug discovery. The results illustrated in Scheme 2 demonstrate that a variety of heterocyclic compounds could be accommodated, including pyridine (**3w**, **3x**), isoquinoline (**3y**), quinolone (**3z**), benzothiazole (**3aa**) and benzofuran (**3t**).

**SCHEME 3:**
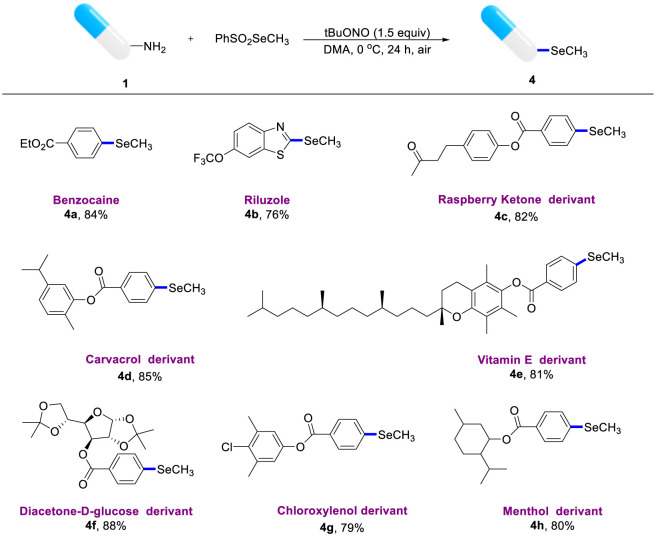
Complex molecular structure modification^a^. a) Optimal conditions: anilines (0.2 mmol), PhSO2SeMe (0.3 mmol), tBuONO (0.3 mmol), DMA (2 mL), 0 °C, air, 24 h. b) Isolated yield.

The versatile methylselenation has inspired us to explore and develop late-stage functional modifications of complex drugs (Scheme 3). To our delight, this simple reaction system proved to be highly useful in several cases. For example, benzocaine and riluzole bearing aniline moiety smoothly underwent site-specific modification to obtain the desired products (**4a**, **4b**). A variety of complex anilines, such as raspberry ketone and carvacrol derivatives also performed well and afforded the expected products (**4c**, **4d**) in 88% and 84% respectively. Remarkably, derivatives of life functional molecules such as Vitamin E and diacetoneglucose underwent this deaminative methylselenation protocol, giving the corresponding products (**4e**, **4f**) in good yields. Similarly, chloroxylenol and menthol derivatives were well-suited to give the anticipated products **4g** and **4h**, respectively.

**SCHEME 4:**
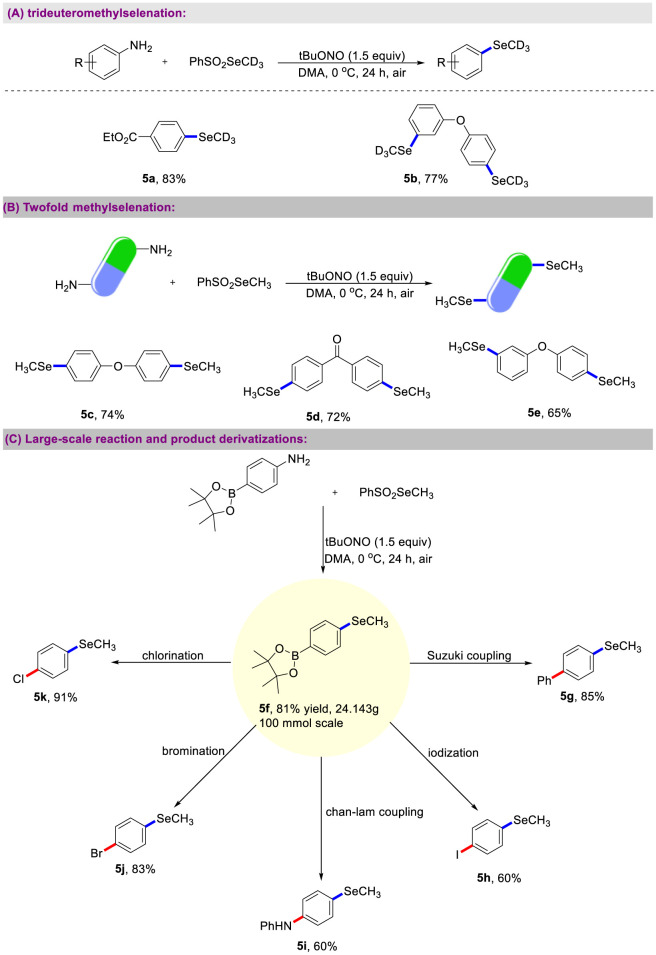
Protocol application. **(A)** Trideuteromethylselenation. **(B)** Twofold methylselenation. **(C)** Large-scale reaction and product derivatizations.

Deuterium labeling is an important tool that can stabilize pharmaceuticals, improve pharmacokinetics, and reduce drug dosage (Kopf et al., 2022). To validate the effectiveness and practicality of this synthetic strategy, we designed and synthesized PhSO_2_SeCD_3_ as a trideuteromethylselenation reagent. Under mild conditions, this reagent was successfully applied to the deaminative mono- and di-trideuteromethylselenation of aromatic amines (Scheme 4A). This method not only possesses high deuterium labeling rate but also enables the cost-effective synthesis of polytrideuteromethylselenated compounds. Remarkably, the deaminative methylselenation of anlines offers a concise route for preparing products containing double aryl methyl selenides (Scheme 4B). During the reaction, we also detected a small amount of by-products resulting from deaminative protonation, which caused the yield of the double reaction to be somewhat lower compared to the single reaction. Product separation via chromatographic column is tedious. Ideally, the subsequent coupling reaction for product derivatization can be performed directly on the crude aryl methyl selenide products without prior purification. We studied the sequential C-N cleavage methylselenation of anilines followed by palladium-catalyzed Suzuki cross-coupling (Scheme 4C). The reaction mixture was filtered upon completion of the methylselenation process, and the DMA solution was then easily removed. Treatment of the crude product with PhB(OH)_2_ afforded the Suzuki product **5g** in high yield. The utility of this methylselenation product was further demonstrated in the straightforward synthesis of *p*-halo phenyl methyl selenides (**5k**, **5j**, **5h**) from 4,4,5,5-tetramethyl-2-(4-(methylselanyl)phenyl)-1,3,2-dioxaborolane **5f**. In these reactions, the Bpin functional group was chemo-selectively transformed into halo groups. Next, we combined the developed methylselenation with copper-catalyzed Chan-Lam coupling, as demonstrated in the preparation of **5i** without isolating the **5f** intermediate. This continuous reaction is due to the fact that the generated intermediates are clean, and only through conventional post-experimental work-up, sequential coupling reaction can undoubtedly accelerate the synthesis of complex molecules.

Compound **4c** exhibits enhanced anti-proliferative activity over the standard agent in HCT116 colon cancer cells. (A) Dose-response curve of the standard agent (IC_50_ = 99.29 ± 15.44 µM). (B) Dose-response curve of Compound **4c** (IC_50_ = 46.95 ± 2.2 µM). HCT116 cells were treated with compounds for 24 h. Cell survival rates were measured and normalized to controls. Data represent mean ± SD of three independent experiments (n = 3).

**SCHEME 5:**
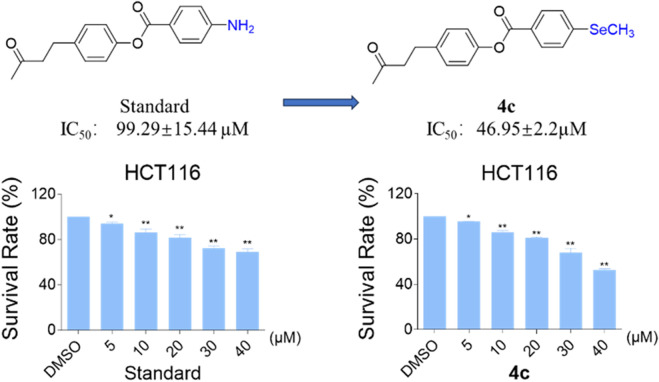
Anti-tumor activity of aryl methyl selenide.

Selenide compounds often exhibit antitumor activity through molecular structural modifications, emerging as a promising class of candidate drugs (Gandin et al., 2018). We evaluated the antitumor activity of the prepared aryl methyl selenides (Scheme 5), confirming that the late-stage methylselenation of anilines produced compound **4c,** which demonstrated significantly stronger inhibitory activity against HCT116 cells compared to the standard. At high concentrations (40 μM), the survival rate of cells treated with **4c** was less than 50%, whereas the survival rate in the standard group remained above 70%. These results highlight the notable dose-dependent inhibitory effect of **4c**, underscoring its potential in the development of novel antitumor drugs.

**SCHEME 6:**
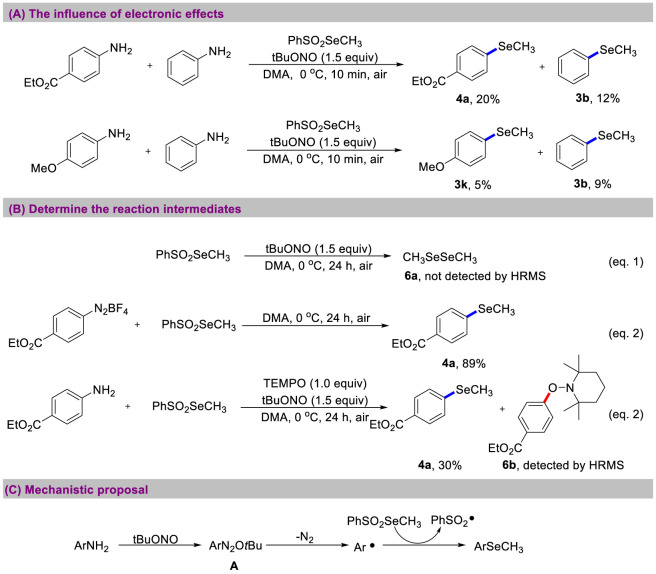
Investigation of mechanism. **(A)** The influence of electronic effects. **(B)** Determine the reaction intermediates. **(C)** Mechanistic proposal.

To gain an in-depth understanding of the methylselenation mechanism, we conducted experiments to investigate the influence of electronic effect (Scheme 6A). When ethyl 4-aminobenzoate and aniline were mixed in a 1:1 ratio, the reaction proceeded for 10 min, generating **4a** and **3b** in a 5:3 ratio. In contrast, a mixture of *p*-anisidine and aniline produced **3k** and **3b** in a 5:9 ratio, suggesting that anilines bearing electron-withdrawing substituents are significantly more reactive than those with electron-donating substituents. Then, PhSO_2_SeCH_3_ was subjected to the reaction conditions (Scheme 6B). CH_3_SeSeCH_3_ was not detected by HRMS and nearly all of the starting material was recovered (Equation 1). When the diazonium salt was reacted with PhSO_2_SeCH_3_ without the presence of tBuONO (Equation 2), the target product **4a** was obtained in 89% yield, demonstrating that tBuONO is essential for the diazotization step. Next, we performed the reaction between benzocaine and PhSO_2_SeCH_3_ in the presence of TEMPO. The yield of **4a** decreased to 30%, and the radical-trapped compound **6b** was detected by HRMS (Equation 3). This experiment suggests the involvement of aryl radical species in the current transformations. According to these results, we propose a radical mechanism for the methylselenation of anilines (Scheme 6C). First, diazotization of anilines with *t*BuONO produces diazonium salt **A** (Zhang et al., 2021). Then, homolytic cleavage of the diazonium salt generates an aryl radical with the release of N_2_. Finally, the *in-situ* generated aryl radical reacts with PhSO_2_SeCH_3_ to afford the aryl methyl selenides and a sulfonyl radical (Zhao et al., 2018).

## Conclusion

In summary, we have demonstrated a novel protocol to access significant aryl methyl selenides. The outstanding advantages of this strategy are as follows: 1) PhSO_2_SeCH_3_ serves as a versatile methylselenation reagent. 2) Inexpensive and commercially available anilines act as aryl radical precursors. The reaction system is mild and metal-free, effectively avoiding metal contamination of the selenide products. 3) Nice functional group tolerance enables double methylselenation, deuterium labeling and late-stage methylselenation of aniline-containing pharmaceuticals. The direct conversion of NH_2_ groups of anilines into SeCH_3_ was successfully performed on a 24g scale. We believe that PhSO_2_SeCH_3_ will find promising applications in the field of pharmaceutical chemistry.

## Data Availability

The original contributions presented in the study are included in the article/Supplementary Material, further inquiries can be directed to the corresponding author.
