# Supplementary material for: Deaminative methylselenation of anlines with PhSO2SeCH3
Source: Front Chem. 2026 Mar 31;14:1762521. doi: 10.3389/fchem.2026.1762521 (PMC13076583; doi:10.3389/fchem.2026.1762521)
Supplement: Supplementary file 1 [file Table1.docx]

Deaminative methylselenation of anlines with PhSO_2_SeCH_3_

Limei Wang^1†^, Bo Zhang^2†^, Yan Yan^3†^, Lijuan Liu^1^, Ge Ou^4^ and Ning Xiao^5,^*

1 Pharmaceutical Management Department, Jilin FAW General Hospital, Changchun 130000, China

2 Medical Equipment Department, Jilin FAW General Hospital, Changchun 130000, China

3 Clinical Laboratory Department, Jilin FAW General Hospital, Changchun 130000, China

4 Neurosurgery Department, Jilin FAW General Hospital, Changchun 130000, China

5 Office of Clinical Trial Institutions, Jilin FAW General Hospital, Changchun 130000, China

* Correspondence: xiaoning900711@163.com

**Table of Contents**

**(1) General considerations, experimental data…….…S2-S32**

**(2) ^1^H, ^13^C and ^19^F NMR spectra of products….…….S33-S80**

**(3) HRMS spectra of products………………….…..S81-S104**

**General Information**

^1^H NMR (500 MHz), ^13^C NMR (125 MHz) and ^19^F NMR (470 MHz) spectra were recorded in CDCl_3_ and DMSO-D6 solutions using a Zhongke Oxford Qone AS400. High-resolution mass spectra were recorded on an ESI-Q-TOF mass spectrometer. Analysis of crude reaction mixture was done on the Varian 4000 GC/MS and 1200 LC. All reactions were conducted using standard Schlenk techniques. Column chromatography was performed using EM silica gel 60 (300−400 m). Cyclic voltammetry data were measured with a Shanghai Chenhua potentiostat (CHI660E).

**General Experimental Procedures**

**Typical procedure for the preparation of PhSO_2_SeCH_3_：**

Sodium benzenesulfinate (100 mmol), Se (120 mmol), CH_3_I (100 mmol) along with a stirring bar are added into a 500 mL reaction tube. Subsequently, 100 mL DMF was added to dissolve the compounds. After that, the reaction tube was tightened with a polytetrafluoroethylene stopper and placed in an aluminum block to heat up to 50°C, where it was allowed to react for 24 hours. After the reaction was complete, it was cooled to room temperature, and the DMF solvent was removed by rotary evaporation. Then add water and ethyl acetate for extraction, dry, and spin dry the organic solvent to obtain a yellow liquid (21.0 g, 89% yield). **^1^H NMR** (400 MHz, Chloroform-*d*) δ 7.90-7.88 (m, 2H), 7.67-7.63 (m, 1H), 7.59-7.55 (m, 2H), 2.64 (s, 3H). **^13^C NMR** (101 MHz, Chloroform-*d*) δ 146.37, 133.70, 129.31, 126.50, 12.93. **HRMS** (ESI): calcd for C_7_H_8_O_2_NaSSe [M + Na]^+^ 258.9308, found 258.9305.

**
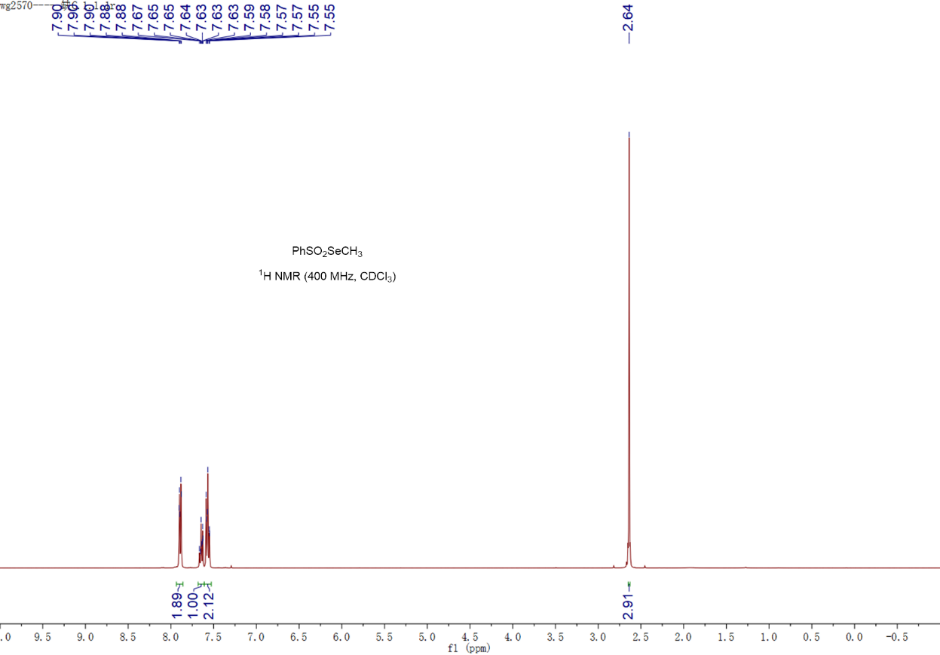
**

**
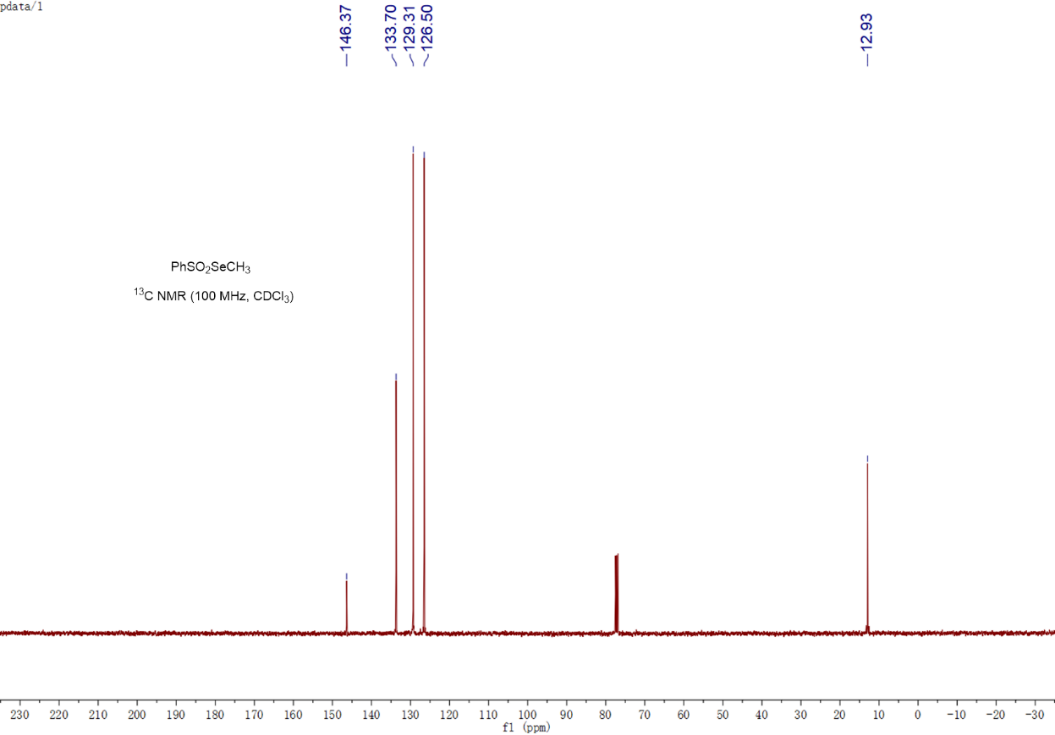
**

**
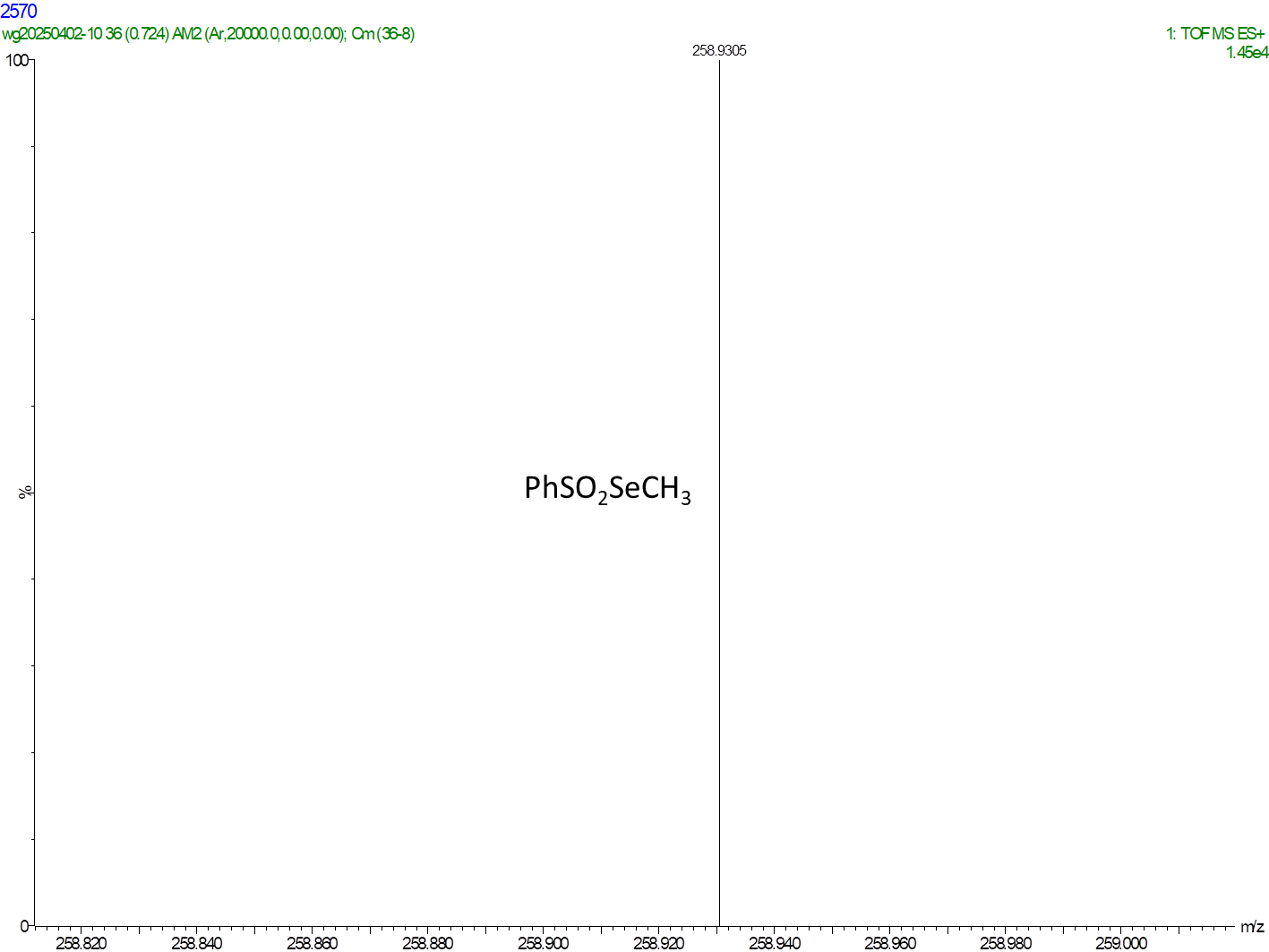
**

**Typical procedure for the preparation of PhSO_2_SeCD_3_：**

Sodium benzenesulfinate (100 mmol), Se (120 mmol), CD_3_I (100 mmol) along with a stirring bar are added into a 500 mL reaction tube. Subsequently, 100 mL DMF was added to dissolve the compounds. After that, the reaction tube was tightened with a polytetrafluoroethylene stopper and placed in an aluminum block to heat up to 50°C, where it was allowed to react for 24 hours. After the reaction was complete, it was cooled to room temperature, and the DMF solvent was removed by rotary evaporation. Then add water and ethyl acetate for extraction, dry, and spin dry the organic solvent to obtain a yellow liquid (20.5 g, 86% yield). **^1^H NMR** (400 MHz, Chloroform-*d*) δ 7.89-7.86 (m, 2H), 7.66-7.62(m, 1H), 7.58-7.54 (m, 2H). **^13^C NMR** (101 MHz, Chloroform-*d*) δ 146.38, 133.73, 129.34, 126.47. **HRMS** (ESI): calcd for C_7_H_5_D_3_O_2_NaSSe [M + Na]^+^ 261.9496, found 261.9491.


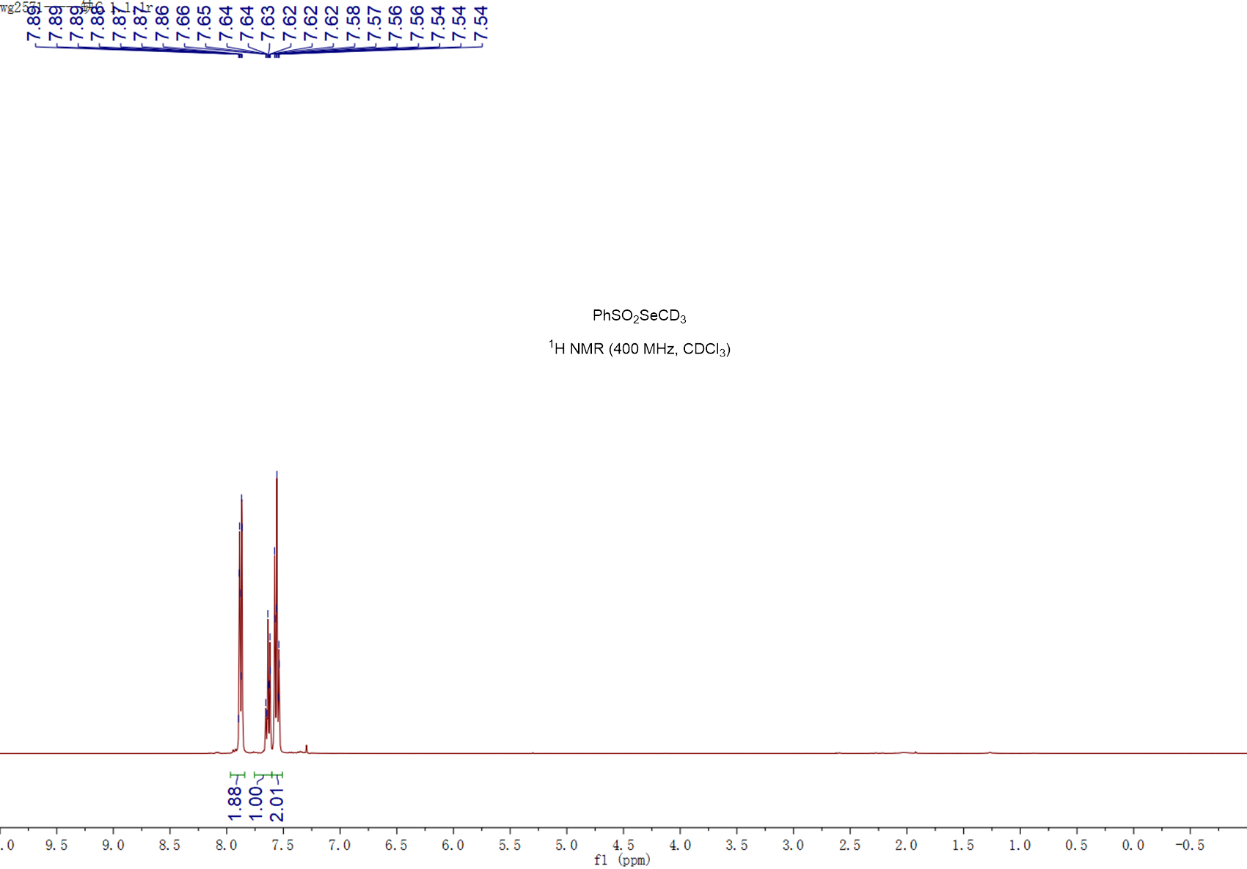


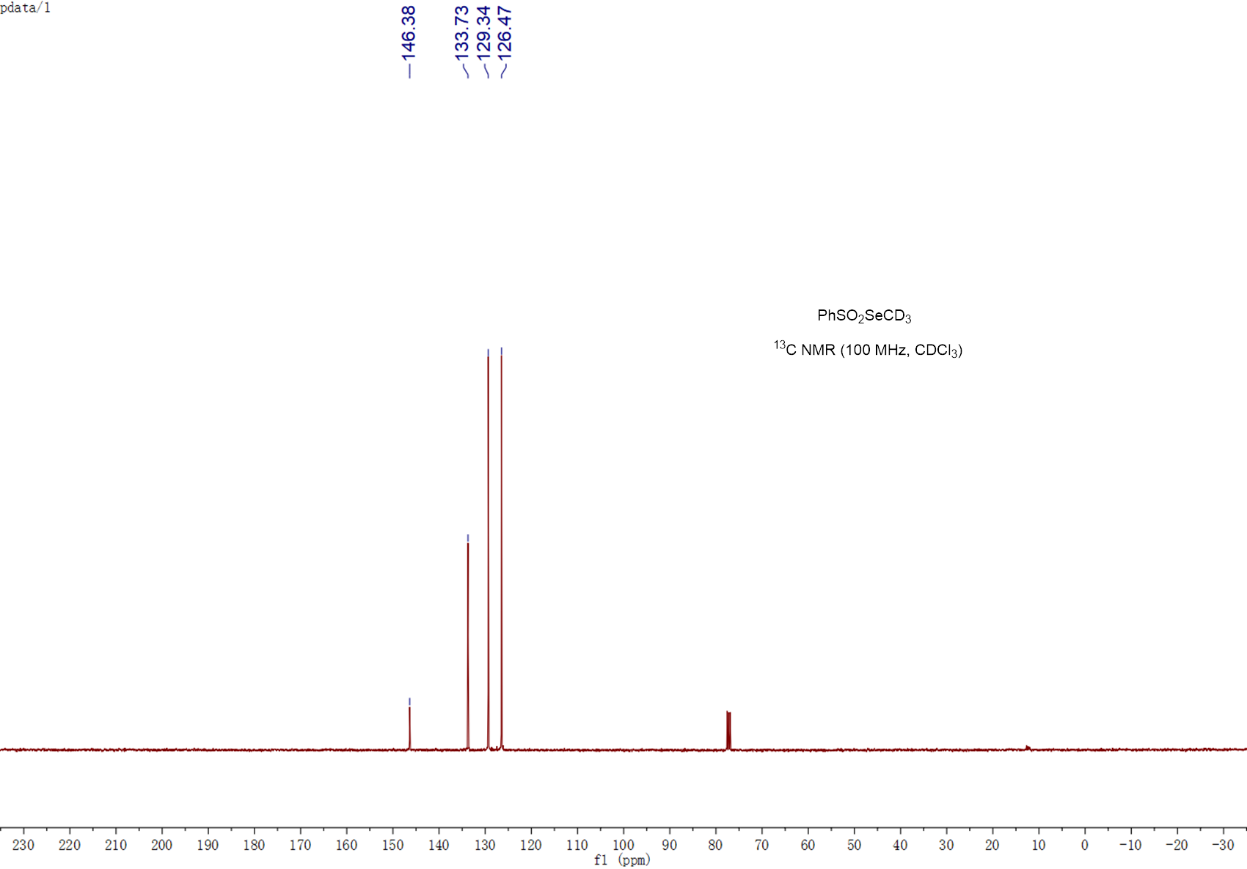


**
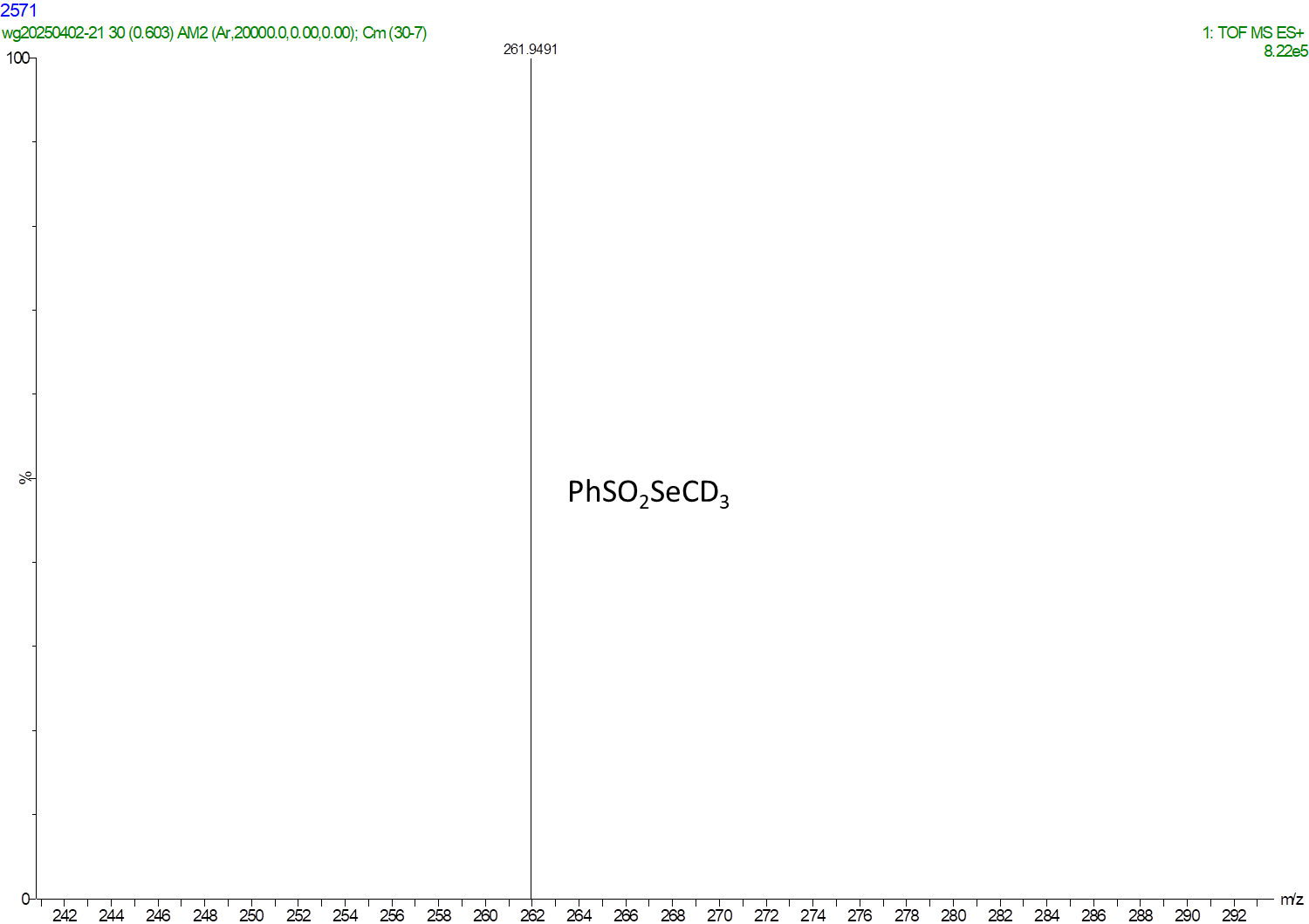
**

**Cell Experiment Method**

The HCT116 cell line was purchased from the Cell Bank of the Chinese Academy of Sciences (CBCAS, Shanghai, China). The culture conditions for this cell line are 1640 and McCoy's 5A medium containing 10% fetal bovine serum (FBS) respectively, in a humid environment at 37°C with 5% CO₂. During sub - culturing, HCT116 cells were digested with an appropriate amount of trypsin for 15 seconds. Then, an appropriate amount of medium containing 10% FBS was added to terminate the digestion. The medium with cells was placed in a centrifuge tube and centrifuged at 1200 rpm for 3 minutes. After centrifugation, the supernatant was discarded, and the cells were resuspended in an appropriate amount of medium. In a six - well plate, 1 mL of medium and 150 μL of cell suspension were added to each well, shaken well and inoculated into the six - well plate. The cells were cultured in a humid environment at 37°C with 5% CO₂ for 24 hours before adding drugs. After adding drugs, the cells continued to be treated for another 24 hours, and then the cells were collected. The cell survival rate was determined by trypan blue staining. The determination of the cell survival rate was consistent with previous literature.

Reference: Zheng P, Xia Y, Shen X, Lu H, Chen Y, Xu C, Qiu C, Zhang Y, Zou P, Cui R, Huang X. Combination of TrxR1 inhibitor and lenvatinib triggers ROS-dependent cell death in human lung cancer cells. Int J Biol Sci. 2024 Jan 1;20(1):249-264.

**Scheme 5.** Anti-tumor activity of aryl methyl selenide


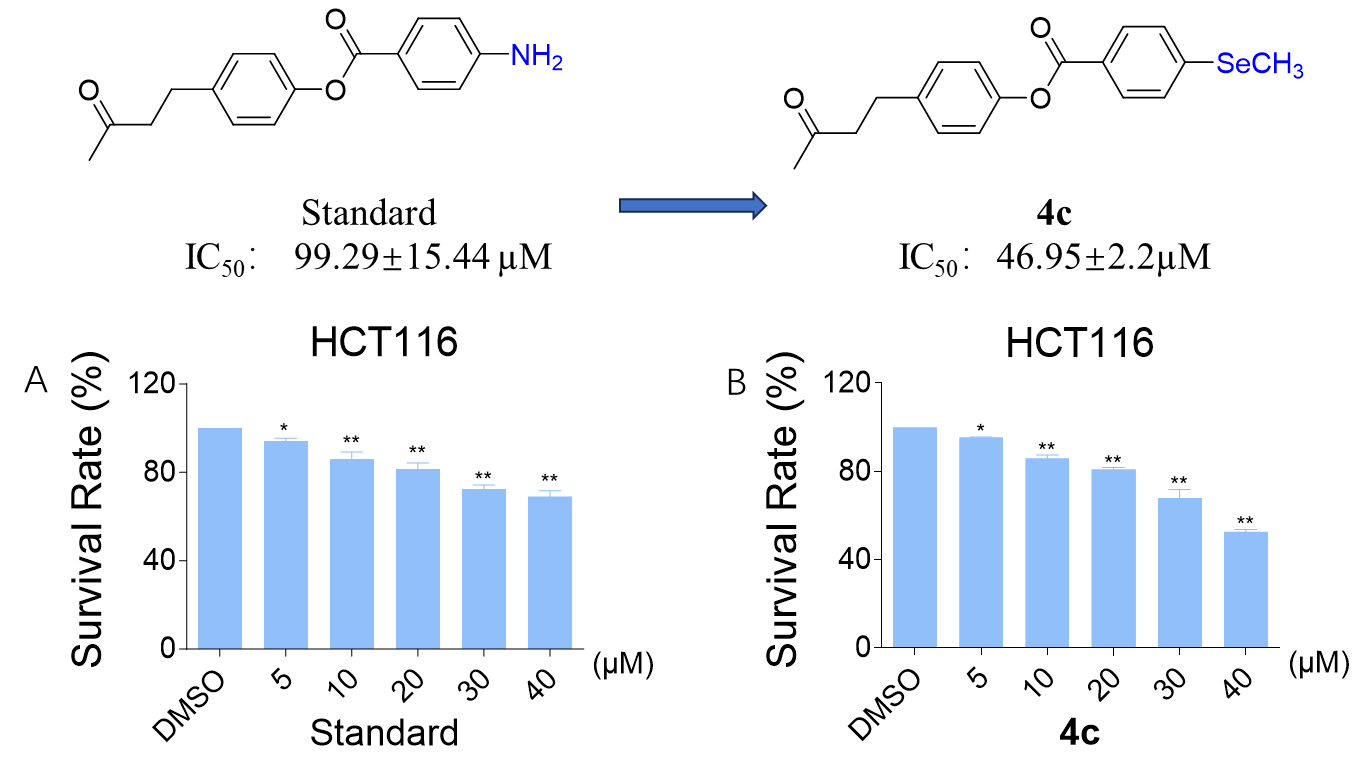


Compound **4c** exhibits enhanced anti-proliferative activity over the standard agent in HCT116 colon cancer cells. (A) Dose-response curve of the standard agent (IC_50_ = 99.29±15.44 µM). (B) Dose-response curve of Compound **4c** (IC_50_ = 46.95±2.2µM). HCT116 cells were treated with compounds for 24 hours. Cell survival rates were measured and normalized to controls. Data represent mean ± SD of three independent experiments (n=3).

**Typical procedure for the preparation of 3：**

aniline (0.2 mmol), PhSO_2_SeCH_3_ (0.3 mmol), tBuONO (0.3 mmol) along with a stirring bar into a 35 mL reaction tube. Subsequently, 2 mL DMA was added to dissolve the compounds. After that, the reaction tube was tightened with a polytetrafluoroethylene stopper and placed in ice-bath, where it was allowed to react for 24 hours. After the reaction was complete, adding 30 mL water and EtOAc to carry out extraction and dry, then the organic solvent was removed by rotary evaporation. Next, silica gel powder was added for drying, and finally, the target product **3** was purified through column chromatography.

**Gram-scale synthesis of 5f:**

4-(4,4,5,5-tetramethyl-1,3,2-dioxaborolan-2-yl)aniline (100 mmol), PhSO_2_SeCH_3_ (150 mmol), tBuONO (150 mmol) along with a stirring bar into a 2500 mL reaction tube. Subsequently, 500 mL DMA was added to dissolve the compounds. After that, the reaction tube was tightened with a rubber stopper and placed in ice-bath, where it was allowed to react for 24 hours. After the reaction was complete, adding 500 mL water and EtOAc (3 times) to carry out extraction and dry, then the organic solvent was removed by rotary evaporation. Next, silica gel powder was added for drying, and finally, the target product **5f** was purified through column chromatography.

**Derivatization reaction of 5f:**

4,4,5,5-tetramethyl-2-(4-(methylselanyl)phenyl)-1,3,2-dioxaborolane (0.2 mmol), PhBr (0.3 mmol), Pd(PPh_3_)_4_ (0.02 mmol), K_2_CO_3_ (0.4 mmol), 1 mL DME and 1mL H_2_O were added into 35 mL reaction tube. After that, the reaction tube was tightly sealed with a rubber stopper, and then it was evacuated and filled with nitrogen. Next, the reaction tube was placed in 100 ^o^C oil bath for 24 h. After the reaction was complete, adding 20 mL water and EtOAc (3 times) to carry out extraction and dry, then the organic solvent was removed by rotary evaporation. Next, silica gel powder was added for drying, and finally, the target product **5g** was purified through column chromatography.

4,4,5,5-tetramethyl-2-(4-(methylselanyl)phenyl)-1,3,2-dioxaborolane (0.2 mmol), KI (0.4 mmol), CuI (0.02 mmol), 1,10-phen (0.04 mmol), 1 mL MeOH and 1mL H_2_O were added into 35 mL reaction tube. After that, the reaction tube was tightly sealed with a rubber stopper. Next, the reaction tube was placed in 80 ^o^C oil bath for 24 h. After the reaction was complete, adding 20 mL water and EtOAc (3 times) to carry out extraction and dry, then the organic solvent was removed by rotary evaporation. Next, silica gel powder was added for drying, and finally, the target product **5h** was purified through column chromatography.

4,4,5,5-tetramethyl-2-(4-(methylselanyl)phenyl)-1,3,2-dioxaborolane (0.2 mmol), PhNH_2_ (0.4 mmol), Cu(OAc)_2_ (0.04 mmol), Et_3_N (0.2 mmol), 2 mL CH_3_CN and 100 µL H_2_O were added into 35 mL reaction tube. After that, the reaction tube was tightly sealed with a rubber stopper. Next, the reaction tube was placed in 80 ^o^C oil bath for 24 h. After the reaction was complete, adding 20 mL water and EtOAc (3 times) to carry out extraction and dry, then the organic solvent was removed by rotary evaporation. Next, silica gel powder was added for drying, and finally, the target product **5i** was purified through column chromatography.

4,4,5,5-tetramethyl-2-(4-(methylselanyl)phenyl)-1,3,2-dioxaborolane (0.2 mmol), CuBr_2_ (0.6 mmol), 1 mL MeOH and 100 mL H_2_O were added into 35 mL reaction tube. After that, the reaction tube was tightly sealed with a rubber stopper. Next, the reaction tube was placed in 70 ^o^C oil bath for 24 h. After the reaction was complete, adding 20 mL water and EtOAc (3 times) to carry out extraction and dry, then the organic solvent was removed by rotary evaporation. Next, silica gel powder was added for drying, and finally, the target product **5j** was purified through column chromatography.

4,4,5,5-tetramethyl-2-(4-(methylselanyl)phenyl)-1,3,2-dioxaborolane (0.2 mmol), CuCl_2_ (0.6 mmol), 1 mL MeOH and 100 mL H_2_O were added into 35 mL reaction tube. After that, the reaction tube was tightly sealed with a rubber stopper. Next, the reaction tube was placed in 70 ^o^C oil bath for 24 h. After the reaction was complete, adding 20 mL water and EtOAc (3 times) to carry out extraction and dry, then the organic solvent was removed by rotary evaporation. Next, silica gel powder was added for drying, and finally, the target product **5k** was purified through column chromatography.

**Mechanistic Studies**

Aniline (0.2 mmol), ethyl 4-aminobenzoate (0.2 mmol), PhSO_2_SeCH_3_ (0.3 mmol), tBuONO (0.3 mmol) along with a stirring bar are added into a 35 mL reaction tube. Subsequently, 2 mL DMA was added to dissolve the compounds. After that, the reaction tube was tightened with a polytetrafluoroethylene stopper and placed in ice-bath, where it was allowed to react for 10 minutes. After the reaction was complete, adding 30 mL water and EtOAc to carry out extraction and dry, then the organic solvent was removed by rotary evaporation. Next, silica gel powder was added for drying, and finally, the target product **4a** and **3b** were purified through column chromatography.

Aniline (0.2 mmol), p-anisidine (0.2 mmol), PhSO_2_SeCH_3_ (0.3 mmol), tBuONO (0.3 mmol) along with a stirring bar are added into a 35 mL reaction tube. Subsequently, 2 mL DMA was added to dissolve the compounds. After that, the reaction tube was tightened with a polytetrafluoroethylene stopper and placed in ice-bath, where it was allowed to react for 10 minutes. After the reaction was complete, adding 30 mL water and EtOAc to carry out extraction and dry, then the organic solvent was removed by rotary evaporation. Next, silica gel powder was added for drying, and finally, the target product **3k** and **3b** were purified through column chromatography.

PhSO_2_SeCH_3_ (0.3 mmol), tBuONO (0.3 mmol) along with a stirring bar are added into a 35 mL reaction tube. Subsequently, 2 mL DMA was added to dissolve the compounds. After that, the reaction tube was tightened with a polytetrafluoroethylene stopper and placed in ice-bath, where it was allowed to react for 24 h. After the reaction was complete and the product **6a** was not detected by GC-MS and HRMS.

Diazonium salt (0.2 mmol) and PhSO_2_SeCH_3_ (0.3 mmol) along with a stirring bar are added into a 35 mL reaction tube. Subsequently, 2 mL DMA was added to dissolve the compounds. After that, the reaction tube was tightened with a polytetrafluoroethylene stopper and placed in ice-bath, where it was allowed to react for 24 h. After the reaction was complete and the product **4a** was isolated in 89%.

Ethyl 4-aminobenzoate (0.2 mmol), PhSO_2_SeCH_3_ (0.3 mmol), TEMPO (0.2 mmol), tBuONO (0.3 mmol) along with a stirring bar into a 35 mL reaction tube. Subsequently, 2 mL DMA was added to dissolve the compounds. After that, the reaction tube was tightened with a polytetrafluoroethylene stopper and placed in ice-bath, where it was allowed to react for 24 hours. After the reaction was complete, adding 30 mL water and EtOAc to carry out extraction and dry, then the organic solvent was removed by rotary evaporation. Next, silica gel powder was added for drying, and finally, the target product **4a** was isolated in 30%, and **6b** was detected by HRMS.

**
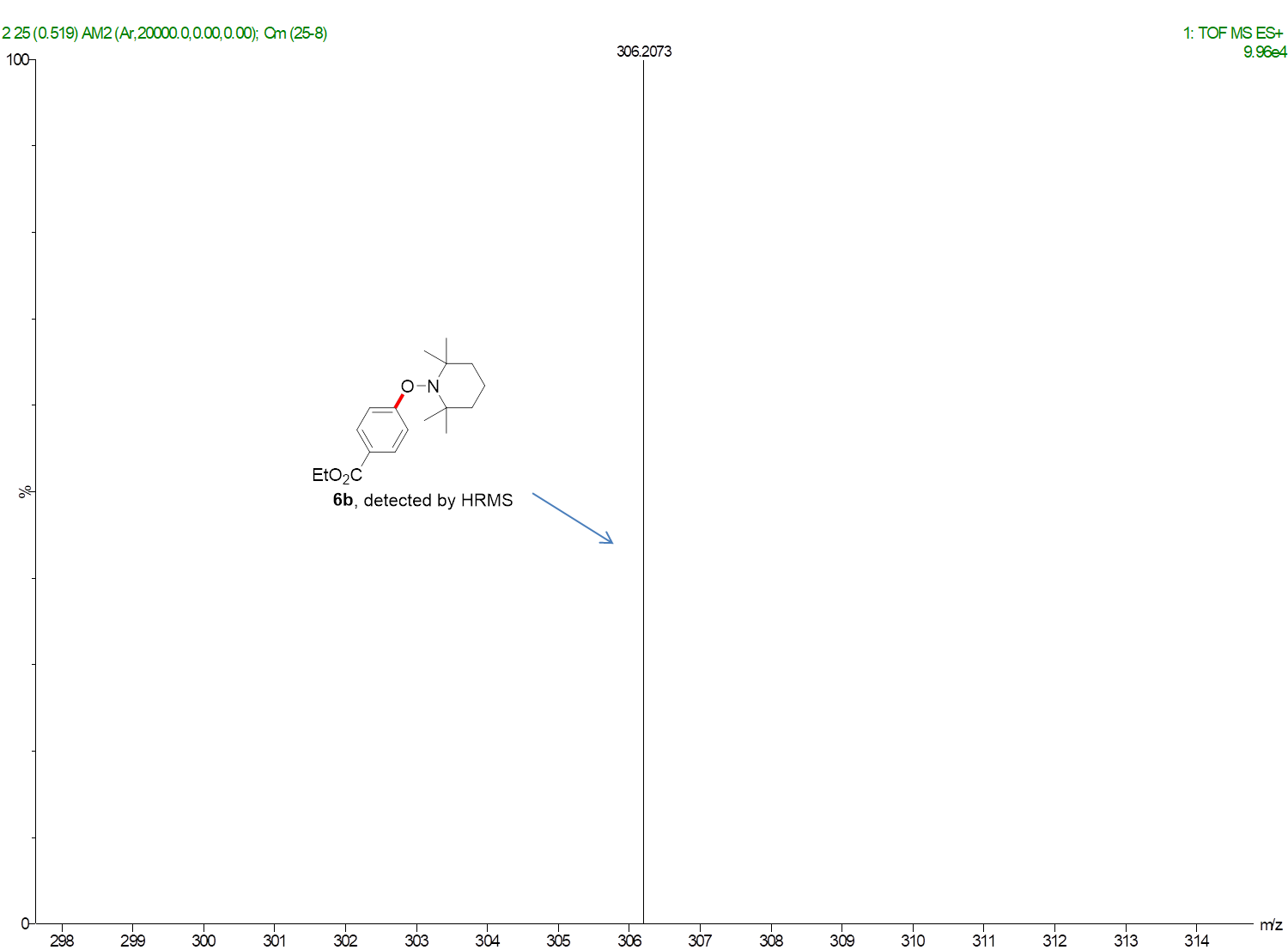
**

**Characterization of Products in Details :**

methyl(p-tolyl)selane

Following the general procedure, using (petroleum ether : EtOAc = 9 : 1) as the eluant afforded a yellow liquid (32.0 mg, 86% yield). **^1^H NMR** (400 MHz, Chloroform-*d*) δ 7.40 (d, *J* = 8.1 Hz, 2H), 7.14 (d, *J* = 7.9 Hz, 2H), 2.38 (d, *J* = 4.7 Hz, 6H). **^13^C NMR** (101 MHz, Chloroform-*d*) δ 136.23, 133.08, 131.00, 130.19, 129.94, 127.96, 21.12, 7.78. **HRMS** (ESI): calcd for C_18_H_10_Se [M ]^+^ 185.9948, found185.9948.

methyl(phenyl)selane

Following the general procedure, using (petroleum ether : EtOAc = 9 : 1) as the eluant afforded a yellow liquid (29.2 mg, 85% yield). **^1^H NMR** (400 MHz, Chloroform-*d*) δ 7.48-7.46 (m, 2H), 7.32-7.22 (m, 3H), 2.40 (s, 3H). **^13^C NMR** (101 MHz, Chloroform-*d*) δ 133.06, 131.91, 130.48, 129.39, 129.09, 126.18, 7.28. **HRMS** (ESI): calcd for C_7_H_8_Se [M ]^+^ 171.9791, found 171.9799.

(4-bromophenyl)(methyl)selane

Following the general procedure, using (petroleum ether : EtOAc = 9 : 1) as the eluant afforded a yellow liquid (41.9 mg, 84% yield). **^1^H NMR** (400 MHz, Chloroform-*d*) δ 7.40 (d, *J* = 8.5 Hz, 2H), 7.31 (d, *J* = 8.5 Hz, 2H), 2.38 (s, 3H). **^13^C NMR** (101 MHz, Chloroform-*d*) δ 132.11, 132.00, 130.91, 120.17, 7.55. **HRMS** (ESI): calcd for C_7_H_7_SeBr [M ]^+^ 249.8896, found 249.8902.

(4-iodophenyl)(methyl)selane

Following the general procedure, using (petroleum ether : EtOAc = 9 : 1) as the eluant afforded a yellow solid (48.2 mg, 81% yield). Mp = 44-45℃. **^1^H NMR** (400 MHz, Chloroform-*d*) δ 7.59 (d, *J* = 8.5 Hz, 2H), 7.18 (d, *J* = 8.3 Hz, 2H), 2.37 (s, 3H). **^13^C NMR** (101 MHz, Chloroform-*d*) δ 137.99, 132.16, 131.94, 91.10, 7.36. **HRMS** (ESI): calcd for C_7_H_7_SeI [M ]^+^ 297.8758, found 297.8756.

methyl(4-(trifluoromethoxy)phenyl)selane

Following the general procedure, using (petroleum ether : EtOAc = 9 : 1) as the eluant afforded a yellow liquid (41.4 mg, 81% yield). **^1^H NMR** (400 MHz, Chloroform-*d*) δ 7.47 (d, *J* = 8.7 Hz, 2H), 7.15 (d, *J* = 8.5 Hz, 2H), 2.40 (s, 3H). **^13^C NMR** (101 MHz, Chloroform-*d*) δ 147.88, 131.83, 130.30, 121.75, 119.24, 7.68. **^19^F NMR** (375 MHz, Chloroform-*d*) δ -57.99 (3F); **HRMS** (ESI): calcd for C_8_H_7_OSeF_3_ [M ]^+^ 255.9614, found 255.9621.

(4-(methylselanyl)phenyl)(trifluoromethyl)sulfane

Following the general procedure, using (petroleum ether : EtOAc = 9 : 1) as the eluant afforded a yellow liquid (42.9 mg, 79% yield). **^1^H NMR** (400 MHz, Chloroform-*d*) δ 7.56 (d, *J* = 8.1 Hz, 2H), 7.45 (d, *J* = 8.5 Hz, 2H), 2.42 (s, 3H). **^13^C NMR** (101 MHz, Chloroform-*d*) δ 137.12, 136.82, 131.04, 130.10, 127.97, 124.91, 121.17, 6.75. **^19^F NMR** (375 MHz, Chloroform-*d*) δ -43.02 (3F); **HRMS** (ESI): calcd for C_8_H_8_F_3_SSe [M + H]^+^ 272.9464, found 272.9462.

methyl(4-(trifluoromethyl)phenyl)selane

Following the general procedure, using (petroleum ether : EtOAc = 9 : 1) as the eluant afforded a yellow solid (43.2 mg, 90% yield). Mp = 89-90℃. **^1^H NMR** (400 MHz, Chloroform-*d*) δ 7.55-7.49 (m, 4H), 2.44 (s, 3H). **^13^C NMR** (101 MHz, Chloroform-*d*) δ 137.62, 129.49, 128.09 (d, *J* = 32.6 Hz), 125.71 (q, *J* = 3.8 Hz), 124.28 (d, *J* = 273.1 Hz), 6.78. **^19^F NMR** (375 MHz, Chloroform-*d*) δ -62.46 (3F); **HRMS** (ESI): calcd for C_8_H_8_F_3_Se [M + H]^+^ 240.9743, found 240.9750.

4-(methylselanyl)benzonitrile

Following the general procedure, using (petroleum ether : EtOAc = 9 : 1) as the eluant afforded a yellow solid (35.1 mg, 89% yield). Mp = 51-52℃. **^1^H NMR** (400 MHz, Chloroform-*d*) δ 7.53 (d, *J* = 8.1 Hz, 2H), 7.45 (d, *J* = 8.4 Hz, 2H), 2.43 (s, 3H). **^13^C NMR** (101 MHz, Chloroform-*d*) δ 140.41, 132.24, 129.15, 119.03, 108.93, 6.65. **HRMS** (API): calcd for C_8_H_7_NSe [M ]^+^ 196.9744, found 196.9746.

(2,6-dimethoxyphenyl)(methyl)selane

Following the general procedure, using (petroleum ether : EtOAc = 9 : 1) as the eluant afforded a yellow solid (40.8 mg, 88% yield). Mp = 44-45℃. **^1^H NMR** (400 MHz, Chloroform-*d*) δ 7.28-7.25 (m, 1H), 6.59 (d, *J* = 8.3 Hz, 2H), 3.92 (s, 6H), 2.30 (s, 3H). **^13^C NMR** (101 MHz, Chloroform-*d*) δ 160.43, 129.41, 104.02, 56.25, 7.72. **HRMS** (ESI): calcd for C_9_H_12_O_2_Se [M ]^+^ 232.0003, found 232.0000.

4-(methylselanyl)benzaldehyde

Following the general procedure, using (petroleum ether : EtOAc = 9 : 1) as the eluant afforded a yellow liquid (32.4 mg, 81% yield). **^1^H NMR** (400 MHz, Chloroform-*d*) δ 9.96 (s, 1H), 7.77 (d, *J* = 8.3 Hz, 2H), 7.52 (d, *J* = 8.3 Hz, 2H), 2.46 (s, 3H). **^13^C NMR** (101 MHz, Chloroform-*d*) δ 191.52, 142.48, 133.98, 130.01, 128.86, 6.49. **HRMS** (ESI): calcd for C_8_H_9_OSe [M + H]^+^ 200.9819, found 200.9821.

(4-methoxyphenyl)(methyl)selane

Following the general procedure, using (petroleum ether : EtOAc = 9 : 1) as the eluant afforded a yellow liquid (31.9 mg, 79% yield). **^1^H NMR** (400 MHz, Chloroform-*d*) δ 7.46 (d, *J* = 8.7 Hz, 2H), 6.87 (d, *J* = 8.7 Hz, 2H), 3.83 (s, 3H), 2.35 (s, 3H). **^13^C NMR** (101 MHz, Chloroform-*d*) δ 158.92, 133.54, 121.64, 114.90, 55.36, 8.75. **HRMS** (ESI): calcd for C_8_H_10_OSe [M ]^+^ 201.9897, found 201.9903.

methyl 2-(methylselanyl)benzoate

Following the general procedure, using (petroleum ether : EtOAc = 9 : 1) as the eluant afforded a yellow solid (28.1 mg, 61% yield). Mp = 48-49℃. **^1^H NMR** (400 MHz, Chloroform-*d*) δ 8.08 (dd, *J* = 7.9, 1.6 Hz, 1H), 7.48 (ddd, *J* = 8.6, 7.2, 1.6 Hz, 1H), 7.42 (dd, *J* = 8.2, 1.3 Hz, 1H), 7.25 (ddd, *J* = 8.1, 7.1, 1.3 Hz, 1H), 3.97 (s, 3H), 2.30 (s, 3H). **^13^C NMR** (101 MHz, Chloroform-*d*) δ 167.36, 138.99, 132.77, 131.61, 127.89, 127.18, 124.35, 52.29, 6.37. **HRMS** (ESI): calcd for C_9_H_10_O_2_NaSe [M + Na]^+^ 252.9744, found 252.9752.

(2-chlorophenyl)(methyl)selane

Following the general procedure, using (petroleum ether : EtOAc = 9 : 1) as the eluant afforded a yellow liquid (28.4 mg, 69% yield). **^1^H NMR** (400 MHz, Chloroform-*d*) δ 7.37 (d, *J* = 7.8 Hz, 1H), 7.30-7.15 (m, 2H), 7.17 (t, *J* = 7.4 Hz, 1H), 2.38 (s, 3H). **^13^C NMR** (101 MHz, Chloroform-*d*) δ 133.90, 132.79, 129.34, 128.56, 127.36, 126.57, 6.41. **HRMS** (ESI): calcd for C_7_H_7_ClSe [M + Na]^+^ 205.9402, found 205.9393.

[1,1'-biphenyl]-2-yl(methyl)selane

Following the general procedure, using (petroleum ether : EtOAc = 9 : 1) as the eluant afforded a yellow solid (30.7 mg, 62% yield). Mp = 61-62℃. **^1^H NMR** (400 MHz, Chloroform-*d*) δ 7.50-7.42 (m, 6H), 7.37-7.27 (m, 3H), 2.26 (s, 3H). **^13^C NMR** (101 MHz, Chloroform-*d*) δ 143.02, 141.66, 132.10, 129.89, 129.07, 128.40, 128.27, 128.11, 127.72, 125.66, 7.12. **HRMS** (ESI): calcd for C_13_H_13_Se [M + H]^+^ 249.0182, found 249.0173.

methyl(2-phenoxyphenyl)selane

Following the general procedure, using (petroleum ether : EtOAc = 9 : 1) as the eluant afforded a yellow solid (31.1 mg, 59% yield). Mp = 77-78℃. **^1^H NMR** (400 MHz, Chloroform-*d*) δ 7.48-7.35 (m, 3H), 7.22-7.11 (m, 3H), 7.04-7.02 (m, 2H), 6.91-6.89 (m, 1H), 2.34 (s, 3H). **^13^C NMR** (101 MHz, Chloroform-*d*) δ 157.16, 154.77, 129.81, 129.40, 126.94, 124.78, 124.63, 123.27, 118.96, 118.26, 5.32. **HRMS** (API): calcd for C_13_H_12_OSe [M]^+^ 264.0053, found 264.0054.

2-(methylselanyl)benzaldehyde

Following the general procedure, using (petroleum ether : EtOAc = 9 : 1) as the eluant afforded a yellow solid (25.6 mg, 64% yield). Mp = 49-50℃. **^1^H NMR** (400 MHz, Chloroform-*d*) δ 10.18 (s, 1H), 7.85-7.83 (m, 1H), 7.56-7.48 (m, 2H), 7.41-7.36 (m, 1H), 2.33 (s, 3H). **^13^C NMR** (101 MHz, Chloroform-*d*) δ 192.57, 135.65, 134.24, 133.86, 127.84, 124.86, 5.86. **HRMS** (ESI): calcd for C_8_H_9_OSe [M + H]^+^ 200.9819, found 200.9816.

methyl 3-methoxy-2-(methylselanyl)benzoate

Following the general procedure, using (petroleum ether : EtOAc = 9 : 1) as the eluant afforded a yellow liquid (31.2 mg, 60% yield). **^1^H NMR** (400 MHz, Chloroform-*d*) δ 7.35-7.30 (m, 1H), 7.25 (d, *J* = 6.9 Hz, 1H), 6.99 (d, *J* = 8.0 Hz, 1H), 3.95 (d, *J* = 2.2 Hz, 6H), 2.29 (s, 3H). **^13^C NMR** (101 MHz, Chloroform-*d*) δ 168.67, 159.73, 138.00, 128.68, 121.12, 119.53, 113.31, 56.20, 52.45, 8.87. **HRMS** (ESI): calcd for C_10_H_12_O_3_NaSe [M + Na]^+^ 282.9849, found 282.9857.

(4-iodo-2-methylphenyl)(methyl)selane

Following the general procedure, using (petroleum ether : EtOAc = 9 : 1) as the eluant afforded a yellow liquid (44.9 mg, 72% yield). **^1^H NMR** (400 MHz, Chloroform-*d*) δ 7.51-7.46 (m, 2H), 7.03 (d, *J* = 8.2 Hz, 1H), 2.34-2.33 (m, 6H). **^13^C NMR** (101 MHz, Chloroform-*d*) δ 140.04, 138.31, 135.47, 133.13, 129.96, 90.71, 21.45, 6.44. **HRMS** (ESI): calcd for C_8_H_9_SeI [M ]^+^ 311.8914, found 311.8918.

(4-isopropylphenyl)(methyl)selane

Following the general procedure, using (petroleum ether : EtOAc = 9 : 1) as the eluant afforded a yellow liquid (33.4 mg, 78% yield). **^1^H NMR** (400 MHz, Chloroform-*d*) δ 7.41 (d, *J* = 8.2 Hz, 2H), 7.17 (d, *J* = 7.8 Hz, 2H), 2.91 (p, *J* = 6.8 Hz, 1H), 2.37 (s, 3H), 1.27 (d, *J* = 6.9 Hz, 6H). **^13^C NMR** (101 MHz, Chloroform-*d*) δ 147.27, 131.00, 128.36, 127.30, 33.78, 23.99, 7.60. **HRMS** (ESI): calcd for C_10_H_14_Se [M ]^+^ 214.0261, found 214.0255.

ethyl 5-(methylselanyl)benzofuran-2-carboxylate

Following the general procedure, using (petroleum ether : EtOAc = 9 : 1) as the eluant afforded a yellow liquid (36.3 mg, 64% yield). **^1^H NMR** (400 MHz, Chloroform-*d*) δ 7.80 (s, 1H), 7.59-7.49 (m, 3H), 4.48 (q, *J* = 7.1 Hz, 2H), 2.43 (s, 3H), 1.46 (t, *J* = 7.1 Hz, 3H). **^13^C NMR** (101 MHz, Chloroform-*d*) δ 159.47, 154.77, 146.31, 131.16, 128.16, 126.24, 125.35, 113.07, 112.96, 61.67, 14.37, 8.60. **HRMS** (ESI): calcd for C_12_H_12_O_3_NaSe [M + Na]^+^ 306.9849, found 306.9856.

methyl(3,4,5-trimethoxyphenyl)selane

Following the general procedure, using (petroleum ether : EtOAc = 9 : 1) as the eluant afforded a yellow liquid (37.7 mg, 72% yield). **^1^H NMR** (400 MHz, Chloroform-*d*) δ 6.73 (s, 2H), 3.91 (s, 6H), 3.86 (s, 3H), 2.41 (s, 3H). **^13^C NMR** (101 MHz, Chloroform-*d*) δ 146.39, 133.67, 129.29, 126.54, 108.48, 60.94, 56.29, 12.89. **HRMS** (ESI): calcd for C_10_H_14_O_3_Se [M]^+^ 262.0108, found 262.0103.

methyl(naphthalen-1-yl)selane

Following the general procedure, using (petroleum ether : EtOAc = 9 : 1) as the eluant afforded a yellow liquid (37.3 mg, 84% yield). **^1^H NMR** (400 MHz, Chloroform-*d*) δ 8.29 (d, *J* = 8.3 Hz, 1H), 7.88 (d, *J* = 7.7 Hz, 1H), 7.79 (d, *J* = 8.2 Hz, 1H), 7.68 (d, *J* = 7.2 Hz, 1H), 7.62-7.53 (m, 2H), 7.45-7.41 (m, 1H), 2.45 (s, 3H). **^13^C NMR** (101 MHz, Chloroform-*d*) δ 133.88, 133.42, 131.19, 128.81, 128.69, 127.29, 126.76, 126.50, 126.26, 125.95, 7.64. **HRMS** (ESI): calcd for C_11_H_10_Se [M ]^+^ 221.9948, found 221.9939.

3-(methylselanyl)pyridine

Following the general procedure, using (petroleum ether : EtOAc = 9 : 1) as the eluant afforded a yellow liquid (22.5 mg, 65% yield). **^1^H NMR** (400 MHz, Chloroform-*d*) δ 8.69 (s, 1H), 8.47 (d, *J* = 4.0 Hz, 1H), 7.77 (d, *J* = 9.5 Hz, 1H), 7.22 (dd, *J* = 7.8, 4.8 Hz, 1H), 2.41 (s, 3H). **^13^C NMR** (101 MHz, Chloroform-*d*) δ 151.31, 147.45, 138.33, 124.01, 7.53. **HRMS** (ESI): calcd for C_6_H_8_NSe [M + H]^+^ 173.9822, found 173.9828.

2-bromo-3-(methylselanyl)pyridine

Following the general procedure, using (petroleum ether : EtOAc = 9 : 1) as the eluant afforded a yellow liquid (33.1 mg, 66% yield). **^1^H NMR** (400 MHz, Chloroform-*d*) δ 8.18 (d, *J* = 4.6 Hz, 1H), 7.49 (d, *J* = 9.2 Hz, 1H), 7.23 (dd, *J* = 7.8, 4.7 Hz, 1H), 2.36 (s, 3H). **^13^C NMR** (101 MHz, Chloroform-*d*) δ 146.13, 142.75, 136.19, 134.51, 123.41, 7.17. **HRMS** (ESI): calcd for C_6_H_7_NSeBr [M + H]^+^ 251.8927, found 251.8927.

5-(methylselanyl)isoquinoline

Following the general procedure, using (petroleum ether : EtOAc = 9 : 1) as the eluant afforded a yellow liquid (32.1 mg, 72% yield). **^1^H NMR** (400 MHz, Chloroform-*d*) δ 9.27 (s, 1H), 8.64 (s, 1H), 8.04 (d, *J* = 6.0 Hz, 1H), 7.88 (dd, *J* = 14.2, 7.7 Hz, 2H), 7.55 (t, *J* = 7.7 Hz, 1H), 2.45 (s, 3H). **^13^C NMR** (101 MHz, Chloroform-*d*) δ 153.10, 143.73, 136.29, 132.90, 130.53, 127.41, 126.61, 7.68. **HRMS** (ESI): calcd for C_10_H_10_NSe [M + H]^+^ 223.9978, found 223.9980.

3-(methylselanyl)quinoline

Following the general procedure, using (petroleum ether : EtOAc = 9 : 1) as the eluant afforded a yellow liquid (33.4 mg, 75% yield). **^1^H NMR** (400 MHz, Chloroform-*d*) δ 8.96 (s, 1H), 8.19 (s, 1H), 8.10 (d, *J* = 8.4 Hz, 1H), 7.76 (d, *J* = 8.1 Hz, 1H), 7.71 (t, *J* = 7.7 Hz, 1H), 7.57 (t, *J* = 7.5 Hz, 1H), 2.50 (s, 3H). **^13^C NMR** (101 MHz, Chloroform-*d*) δ 152.38, 146.58, 136.78, 129.45, 129.20, 128.78, 127.19, 126.94, 126.01, 7.73. **HRMS** (ESI): calcd for C_10_H_10_NSe [M + H]^+^ 223.9978, found 223.9981.

2-(methylselanyl)benzo[d]thiazole

Following the general procedure, using (petroleum ether : EtOAc = 9 : 1) as the eluant afforded a yellow solid (35.2 mg, 77% yield). Mp = 43-44℃. **^1^H NMR** (400 MHz, Chloroform-*d*) δ 7.96 (d, *J* = 8.1 Hz, 1H), 7.83 (d, *J* = 8.0 Hz, 1H), 7.45 (t, *J* = 7.7 Hz, 1H), 7.34 (t, *J* = 7.6 Hz, 1H), 2.73 (s, 3H).

**^13^C NMR** (101 MHz, Chloroform-*d*) δ 159.99, 154.15, 136.40, 126.07, 124.25, 121.66, 120.99, 8.09. **HRMS** (ESI): calcd for C_8_H_8_NSSe [M + H]^+^ 229.9543, found 229.9551.

ethyl 4-(methylselanyl)benzoate

Following the general procedure, using (petroleum ether : EtOAc = 9 : 1) as the eluant afforded a yellow solid (41.0 mg, 84% yield). Mp = 46-47℃. **^1^H NMR** (400 MHz, Chloroform-*d*) δ 7.94 (d, *J* = 6.3 Hz, 2H), 7.44 (d, *J* = 6.3 Hz, 2H), 4.39 (q, *J* = 7.1 Hz, 2H), 2.43 (s, 3H), 1.42 (t, *J* = 7.1 Hz, 3H). **^13^C NMR** (101 MHz, Chloroform-*d*) δ 166.51, 139.46, 129.95, 128.63, 127.80, 61.00, 14.41, 6.58. **HRMS** (ESI): calcd for C_10_H_13_O_2_Se [M + H]^+^ 245.0081, found 245.0088.

2-(methylselanyl)-6-(trifluoromethoxy)benzo[d]thiazole

Following the general procedure, using (petroleum ether : EtOAc = 9 : 1) as the eluant afforded a yellow solid (47.6 mg, 76% yield). Mp = 37-38℃. **^1^H NMR** (400 MHz, Chloroform-*d*) δ 7.93 (d, *J* = 8.9 Hz, 1H), 7.70 (s, 1H), 7.33 (d, *J* = 8.8 Hz, 1H), 2.74 (s, 3H). **^13^C NMR** (101 MHz, Chloroform-*d*) δ 161.38, 152.66, 145.76, 137.20, 122.20, 121.87, 119.90, 119.31, 113.70, 8.18. **^19^F NMR** (375 MHz, Chloroform-*d*) δ -58.04 (3F); **HRMS** (ESI): calcd for C_9_H_7_NOSSeF_3_ [M + H]^+^ 313.9366, found 313.9370.

4-(3-oxobutyl)phenyl 4-(methylselanyl)benzoate

Following the general procedure, using (petroleum ether : EtOAc = 9 : 1) as the eluant afforded a yellow solid (59.4 mg, 82% yield). Mp = 104-105℃. **^1^H NMR** (400 MHz, Chloroform-*d*) δ 8.08 (d, *J* = 8.4 Hz, 2H), 7.51 (d, *J* = 8.4 Hz, 2H), 7.30-7.26 (m, 2H), 7.15 (d, *J* = 8.4 Hz, 2H), 2.96 (t, *J* = 7.5 Hz, 2H), 2.82 (t, *J* = 7.5 Hz, 2H), 2.47 (s, 3H), 2.20 (s, 3H). **^13^C NMR** (101 MHz, Chloroform-*d*) δ 207.89, 165.22, 149.25, 140.83, 138.72, 130.51, 129.44, 128.67, 126.73, 121.74, 45.22, 30.23, 29.14, 6.57. **HRMS** (ESI): calcd for C_18_H_18_O_3_NaSe [M + Na]^+^ 385.0319, found 385.0323.

5-isopropyl-2-methylphenyl 4-(methylselanyl)benzoate

Following the general procedure, using (petroleum ether : EtOAc = 9 : 1) as the eluant afforded a yellow solid (59.2 mg, 85% yield). Mp = 48-49℃. **^1^H NMR** (400 MHz, Chloroform-*d*) δ 8.12 (d, *J* = 8.5 Hz, 2H), 7.53 (d, *J* = 8.5 Hz, 2H), 7.23 (d, *J* = 7.8 Hz, 1H), 7.10 (d, *J* = 7.8 Hz, 1H), 7.04 (s, 1H), 2.95 (p, *J* = 6.9 Hz, 1H), 2.47 (s, 3H), 2.22 (s, 3H), 1.30 (d, *J* = 6.9 Hz, 6H). **^13^C NMR** (101 MHz, Chloroform-*d*) δ 164.81, 149.50, 148.22, 140.71, 131.01, 130.52, 128.79, 127.44, 126.82, 124.27, 119.97, 33.69, 24.02, 15.93, 6.58. **HRMS** (ESI): calcd for C_18_H_20_O_2_NaSe [M + Na]^+^ 371.0526, found 371.0529.

(R)-2,5,7,8-tetramethyl-2-((4R,8R)-4,8,12-trimethyltridecyl)chroman-6-yl 4-(methylselanyl)benzoate

Following the general procedure, using (petroleum ether : EtOAc = 9 : 1) as the eluant afforded a yellow liquid (101.8 mg, 81% yield). **^1^H NMR** (400 MHz, Chloroform-*d*) δ 8.15 (d, *J* = 8.4 Hz, 2H), 7.54 (d, *J* = 8.4 Hz, 2H), 2.66 (t, *J* = 6.8 Hz, 2H), 2.47 (s, 3H), 2.17 (s, 3H), 2.10 (s, 3H), 2.06 (s, 3H), 1.92-1.79 (m, 2H), 1.67-1.06 (m, 24H), 0.93-0.89 (m, 12H). **^13^C NMR** (101 MHz, Chloroform-*d*) δ 165.07, 149.55, 140.67, 140.47, 130.54, 128.85, 126.99, 126.90, 125.21, 123.21, 117.55, 75.16, 40.51, 39.81, 39.46, 37.64, 37.55, 37.49, 37.38, 32.87, 32.80, 31.32, 31.09, 28.07, 24.90, 24.54, 24.29, 23.79, 22.82, 22.72, 21.14, 20.72, 19.85, 19.78, 19.70, 13.13, 12.28, 11.94, 6.60. **HRMS** (ESI): calcd for C_37_H_56_O_3_NaSe [M + Na]^+^ 651.3292, found 651.3298.

(3aR,5R,6S,6aR)-5-((R)-2,2-dimethyl-1,3-dioxolan-4-yl)-2,2-dimethyltetrahydrofuro[2,3-d][1,3]dioxol-6-yl 4-(methylselanyl)benzoate

Following the general procedure, using (petroleum ether : EtOAc = 9 : 1) as the eluant afforded a yellow solid (80.6 mg, 88% yield). Mp = 70-71℃. **^1^H NMR** (400 MHz, Chloroform-*d*) δ 7.93-7.89 (m, 2H), 7.47-7.44 (m, 2H), 5.99-5.97 (m, 1H), 5.53-5.51 (m, 1H), 4.67-4.65 (m, 1H), 4.40-4.36 (m, 2H), 4.16-4.11 (m, 2H), 2.44-2.43 (m, 3H), 1.60-1.58 (m, 3H), 1.46-1.44 (m, 3H), 1.36-1.35 (m, 3H), 1.31-1.29 (m, 3H). **^13^C NMR** (101 MHz, Chloroform-*d*) δ 165.07, 140.70, 130.04, 128.69, 126.71, 112.42, 109.45, 105.20, 83.44, 80.02, 76.62, 72.64, 67.30, 26.89, 26.80, 26.27, 25.28, 6.54. **HRMS** (ESI): calcd for C_20_H_26_O_7_NaSe [M + Na]^+^ 481.0741, found 481.0738.

4-chloro-3,5-dimethylphenyl 4-(methylselanyl)benzoate

Following the general procedure, using (petroleum ether : EtOAc = 9 : 1) as the eluant afforded a yellow solid (55.9 mg, 79% yield). Mp = 98-99℃. **^1^H NMR** (400 MHz, Chloroform-*d*) δ 8.06 (d, *J* = 8.4 Hz, 2H), 7.51 (d, *J* = 8.4 Hz, 2H), 6.99 (s, 2H), 2.47 (s, 3H), 2.44 (s, 6H). **^13^C NMR** (101 MHz, Chloroform-*d*) δ 165.13, 148.59, 141.02, 137.61, 131.83, 130.49, 128.69, 126.55, 121.57, 20.94, 6.55. **HRMS** (ESI): calcd for C_16_H_15_O_2_NaClSe [M + Na]^+^ 376.9823, found 376.9820.

2-isopropyl-5-methylcyclohexyl 4-(methylselanyl)benzoate

Following the general procedure, using (petroleum ether : EtOAc = 9 : 1) as the eluant afforded a yellow solid (56.6 mg, 80% yield). Mp = 44-45℃. **^1^H NMR** (400 MHz, Chloroform-*d*) δ 7.94 (d, *J* = 8.4 Hz, 2H), 7.45 (d, *J* = 8.4 Hz, 2H), 4.95 (td, *J* = 10.9, 4.4 Hz, 1H), 2.41 (s, 3H), 2.17-2.14 (m, 1H), 1.98 (pd, *J* = 7.0, 2.8 Hz, 1H), 1.77-1.74 (m, 2H), 1.60-1.54 (m, 2H), 1.22-1.08 (m, 2H), 0.97-0.94 (m, 6H), 0.83 (d, *J* = 7.0 Hz, 3H). **^13^C NMR** (101 MHz, Chloroform-*d*) δ 165.93, 139.29, 129.98, 128.74, 128.22, 74.84, 47.33, 41.04, 34.39, 31.50, 26.60, 23.74, 22.13, 20.83, 16.64, 6.61. **HRMS** (ESI): calcd for C_18_H_26_O_2_NaSe [M + Na]^+^ 377.0996, found 377.1002.

ethyl 4-((methyl-d3)selanyl)benzoate

Following the general procedure, using (petroleum ether : EtOAc = 9 : 1) as the eluant afforded a yellow solid (41.0 mg, 83% yield). Mp = 47-48℃. **^1^H NMR** (400 MHz, Chloroform-*d*) δ 7.94 (d, *J* = 8.4 Hz, 2H), 7.45 (d, *J* = 8.4 Hz, 2H), 4.40 (q, *J* = 7.1 Hz, 2H), 1.42 (t, *J* = 7.1 Hz, 3H). **^13^C NMR** (101 MHz, Chloroform-*d*) δ 166.49, 139.34, 129.94, 128.75, 127.89, 60.96, 14.38. **HRMS** (ESI): calcd for C_10_H_10_D_3_O_2_Se [M + H]^+^ 248.0269, found 248.0267.

(methyl-d3)(3-(4-((methyl-d3)selanyl)phenoxy)phenyl)selane

Following the general procedure, using (petroleum ether : EtOAc = 9 : 1) as the eluant afforded a yellow liquid (56.0 mg, 77% yield). **^1^H NMR** (400 MHz, Chloroform-*d*) δ 7.46 (d, *J* = 8.7 Hz, 2H), 7.24 (t, *J* = 7.9 Hz, 1H), 7.17 (d, *J* = 7.8 Hz, 1H), 7.08 (s, 1H), 6.96 (d, *J* = 8.7 Hz, 2H), 6.84 (d, *J* = 10.1 Hz, 1H). **^13^C NMR** (101 MHz, Chloroform-*d*) δ 132.84, 130.11, 124.91, 120.21, 119.85, 116.38.

**HRMS** (ESI): calcd for C_14_H_9_D_6_OSe_2_ [M + H]^+^ 364.9830, found 364.9829.

(oxybis(4,1-phenylene))bis(methylselane)

Following the general procedure, using (petroleum ether : EtOAc = 9 : 1) as the eluant afforded a yellow liquid (52.9 mg, 74% yield). **^1^H NMR** (400 MHz, Chloroform-*d*) δ 7.46 (d, *J* = 8.7 Hz, 2H), 7.24 (t, *J* = 7.9 Hz, 1H), 7.17 (d, *J* = 7.8 Hz, 1H), 7.09 (s, 1H), 6.96 (d, *J* = 8.7 Hz, 2H), 6.85 (d, *J* = 9.4 Hz, 1H), 2.39 (s, 6H). **^13^C NMR** (101 MHz, Chloroform-*d*) δ 157.56, 155.87, 133.68, 132.82, 130.13, 125.48, 124.87, 120.16, 119.86, 116.38, 8.24, 7.16. **HRMS** (ESI): calcd for C_14_H_15_OSe_2_ [M + H]^+^ 358.9453, found 358.9455.

bis(4-(methylselanyl)phenyl)methanone

Following the general procedure, using (petroleum ether : EtOAc = 9 : 1) as the eluant afforded a yellow solid (53.3 mg, 72% yield). Mp = 110-111℃. **^1^H NMR** (400 MHz, Chloroform-*d*) δ 7.71 (d, *J* = 8.4 Hz, 4H), 7.50 (d, *J* = 8.5 Hz, 4H), 2.46 (s, 6H). **^13^C NMR** (101 MHz, Chloroform-*d*) δ 139.17, 134.89, 130.44, 128.55, 6.54. **HRMS** (ESI): calcd for C_15_H_14_ONaSe_2_ [M + Na]^+^ 392.9273, found 392.9277.

methyl(3-(4-(methylselanyl)phenoxy)phenyl)selane

Following the general procedure, using (petroleum ether : EtOAc = 9 : 1) as the eluant afforded a yellow liquid (46.5 mg, 65% yield). **^1^H NMR** (400 MHz, Chloroform-*d*) δ 7.46 (d, *J* = 8.7 Hz, 2H), 7.24 (t, *J* = 7.9 Hz, 1H), 7.17 (d, *J* = 7.8 Hz, 1H), 7.09-7.08 (m, 1H), 6.96 (d, *J* = 8.7 Hz, 2H), 6.85 (dd, *J* = 7.6, 2.8 Hz, 1H), 2.38 (d, *J* = 4.7 Hz, 6H). **^13^C NMR** (101 MHz, Chloroform-*d*) δ 157.57, 132.86, 130.12, 124.92, 120.22, 119.85, 116.41, 8.21, 7.15. **HRMS** (ESI): calcd for C_14_H_14_OSe [M ]^+^ 357.9375, found 357.9373.

4,4,5,5-tetramethyl-2-(4-(methylselanyl)phenyl)-1,3,2-dioxaborolane

Following the general procedure, using (petroleum ether : EtOAc = 9 : 1) as the eluant afforded a yellow liquid (100 mmol, 24.143g, 81% yield). **^1^H NMR** (400 MHz, Chloroform-*d*) δ 7.71 (d, *J* = 8.0 Hz, 2H), 7.43 (d, *J* = 8.0 Hz, 2H), 2.40 (s, 3H), 1.38 (s, 12H). **^13^C NMR** (101 MHz, Chloroform-*d*) δ 136.56, 135.28, 134.81, 131.30, 128.89, 127.76, 83.85, 24.92, 6.67. **HRMS** (ESI): calcd for C_13_H_19_BO_2_Se [M ]^+^ 298.0643, found 298.0647.

[1,1'-biphenyl]-4-yl(methyl)selane

Following the general procedure, using (petroleum ether : EtOAc = 9 : 1) as the eluant afforded a yellow solid (42.1 mg, 85% yield). Mp = 87-88℃. **^1^H NMR** (400 MHz, Chloroform-*d*) δ 7.63-7.61 (m, 2H), 7.54 (brs, 4H), 7.48 (t, *J* = 7.5 Hz, 2H), 7.39 (t, *J* = 7.3 Hz, 1H), 2.44 (s, 3H). **^13^C NMR** (101 MHz, Chloroform-*d*) δ 140.64, 139.26, 131.05, 130.82, 128.90, 127.78, 127.39, 126.98, 7.36. **HRMS** (API): calcd for C_13_H_12_Se [M ]^+^ 248.0104, found 248.0108.

(4-iodophenyl)(methyl)selane

Following the general procedure, using (petroleum ether : EtOAc = 9 : 1) as the eluant afforded a yellow solid (35.7 mg, 60% yield). Mp = 47-48℃. **^1^H NMR** (400 MHz, Chloroform-*d*) δ 7.59 (d, *J* = 8.0 Hz, 2H), 7.18 (d, *J* = 7.9 Hz, 2H), 2.37 (s, 3H). **^13^C NMR** (101 MHz, Chloroform-*d*) δ 137.99, 132.16, 131.96, 91.14, 7.38. **HRMS** (ESI): calcd for C_7_H_7_ISe [M ]^+^ 297.8758, found 297.8760.

4-(methylselanyl)-N-phenylaniline

Following the general procedure, using (petroleum ether : EtOAc = 9 : 1) as the eluant afforded a yellow solid (31.6 mg, 60% yield). Mp = 75-76℃. **^1^H NMR** (400 MHz, Chloroform-*d*) δ 7.44 (s, 2H), 7.31 (d, *J* = 7.5 Hz, 2H), 7.10 (d, *J* = 7.9 Hz, 2H), 7.04-6.97 (m, 3H), 5.73 (s, 1H), 2.37 (s, 3H). **^13^C NMR** (101 MHz, Chloroform-*d*) δ 142.85, 142.30, 133.29, 129.47, 121.81, 121.39, 118.47, 118.14, 8.65. **HRMS** (ESI): calcd for C_13_H_14_NSe [M + H]^+^ 264.0291, found 264.0294.

(4-bromophenyl)(methyl)selane

Following the general procedure, using (petroleum ether : EtOAc = 9 : 1) as the eluant afforded a yellow solid (41.5 mg, 83% yield). Mp = 76-77℃. **^1^H NMR** (400 MHz, Chloroform-*d*) δ 7.40 (d, *J* = 8.1 Hz, 2H), 7.31 (d, *J* = 8.0 Hz, 2H), 2.37 (s, 3H). **^13^C NMR** (101 MHz, Chloroform-*d*) δ 132.10, 132.03, 130.91, 120.18, 7.55. **HRMS** (API): calcd for C_7_H_7_SeBr [M]^+^ 249.8896, found 249.8900.

(4-chlorophenyl)(methyl)selane

Following the general procedure, using (petroleum ether : EtOAc = 9 : 1) as the eluant afforded a yellow liquid (37.5 mg, 91% yield). **^1^H NMR** (400 MHz, Chloroform-*d*) δ 7.38 (d, *J* = 8.4 Hz, 2H), 7.26 (d, *J* = 8.4 Hz, 2H), 2.38 (s, 3H). **^13^C NMR** (101 MHz, Chloroform-*d*) δ 132.31, 131.83, 130.12, 129.19, 7.64. **HRMS** (ESI): calcd for C_7_H_7_SeI [M ]^+^ 297.8758, found 297.8760.

**^1^H, ^13^C and ^19^F NMR spectra of products**

**^1^H NMR (400 MHz, Chloroform-*d*) spectrum of 3a**

**
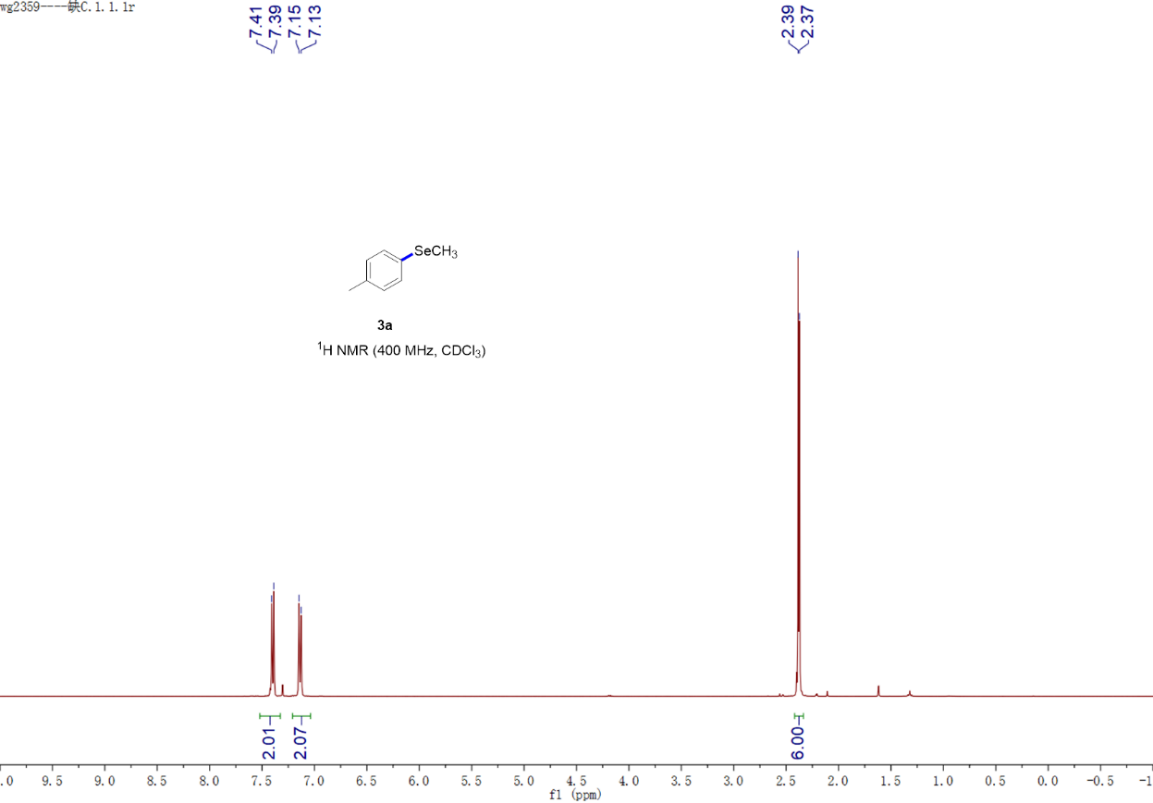
**

**^13^C{^1^H} NMR (100 MHz, Chloroform-*d*) spectrum of 3a**

**
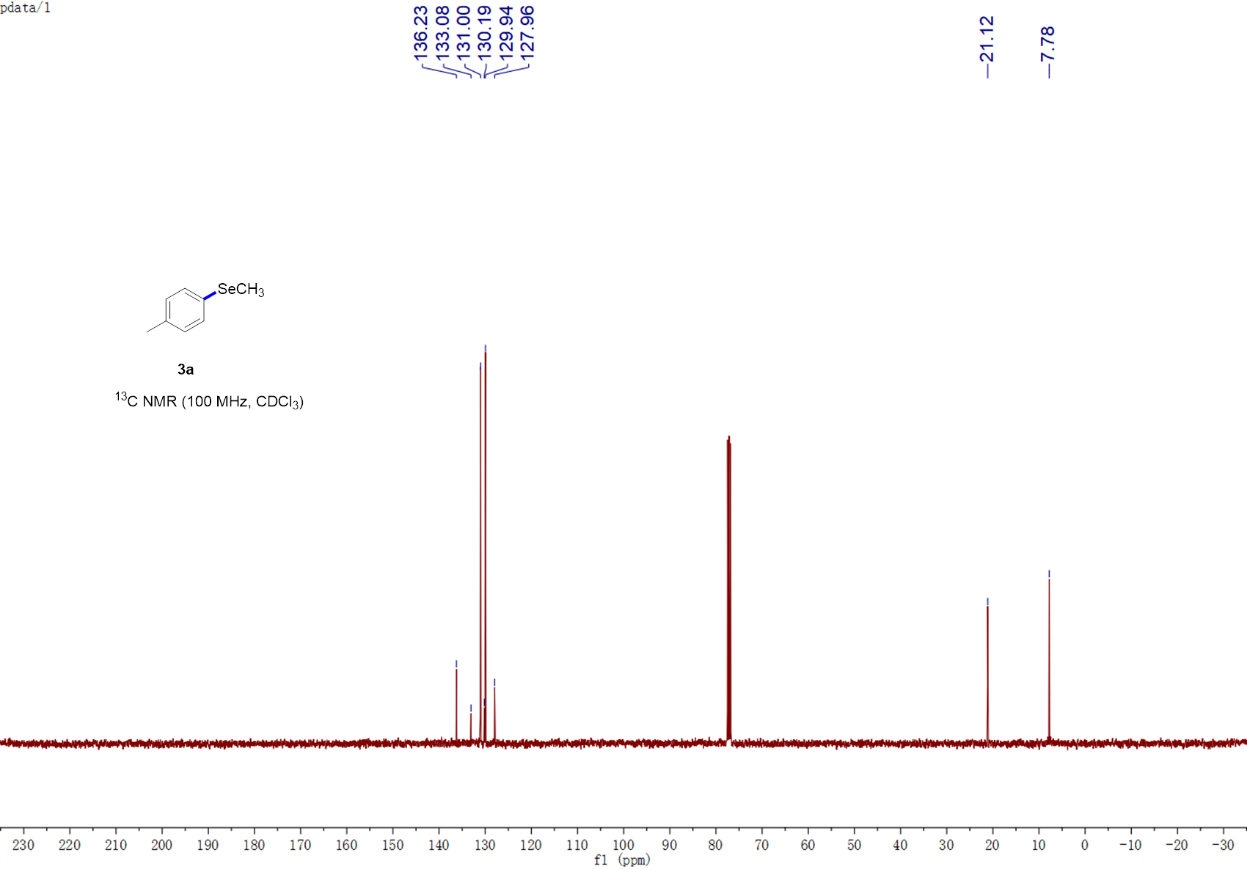
**

**^1^H NMR (400 MHz, Chloroform-*d*) spectrum of 3b**

**
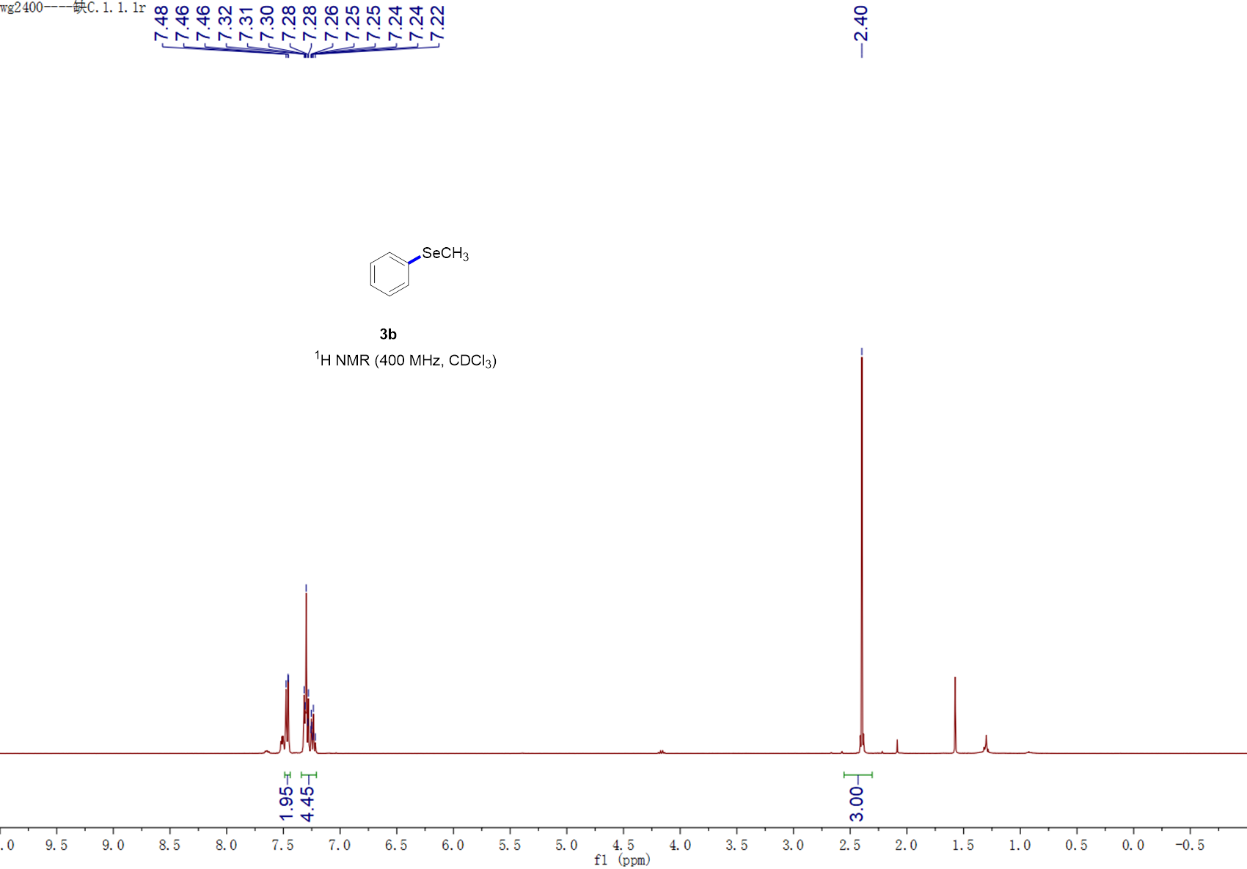
**

**^13^C{^1^H} NMR (100 MHz, Chloroform-*d*) spectrum of 3b**

**
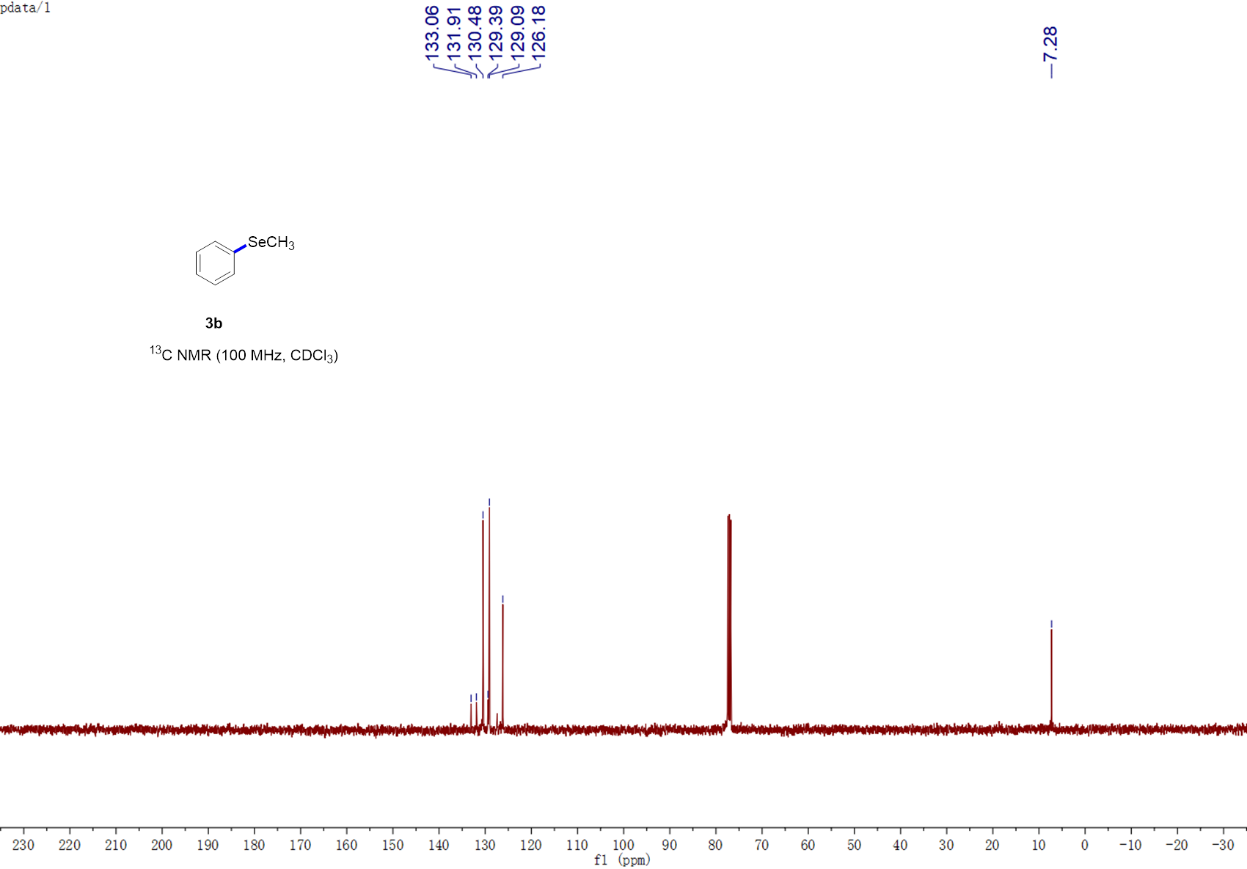
**

**^1^H NMR (400 MHz, Chloroform-*d*) spectrum of 3c**

**
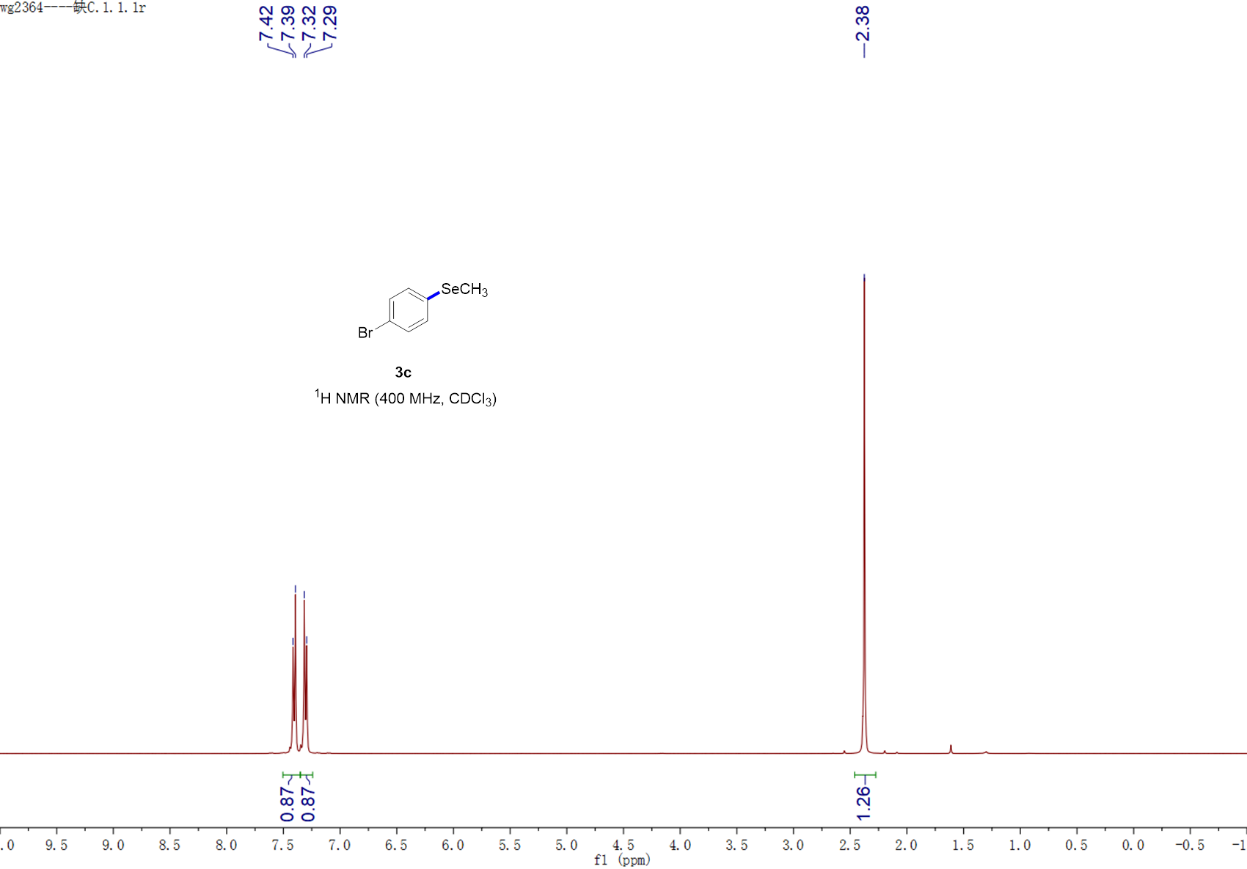
**

**^13^C{^1^H} NMR (100 MHz, Chloroform-*d*) spectrum of 3c**

**
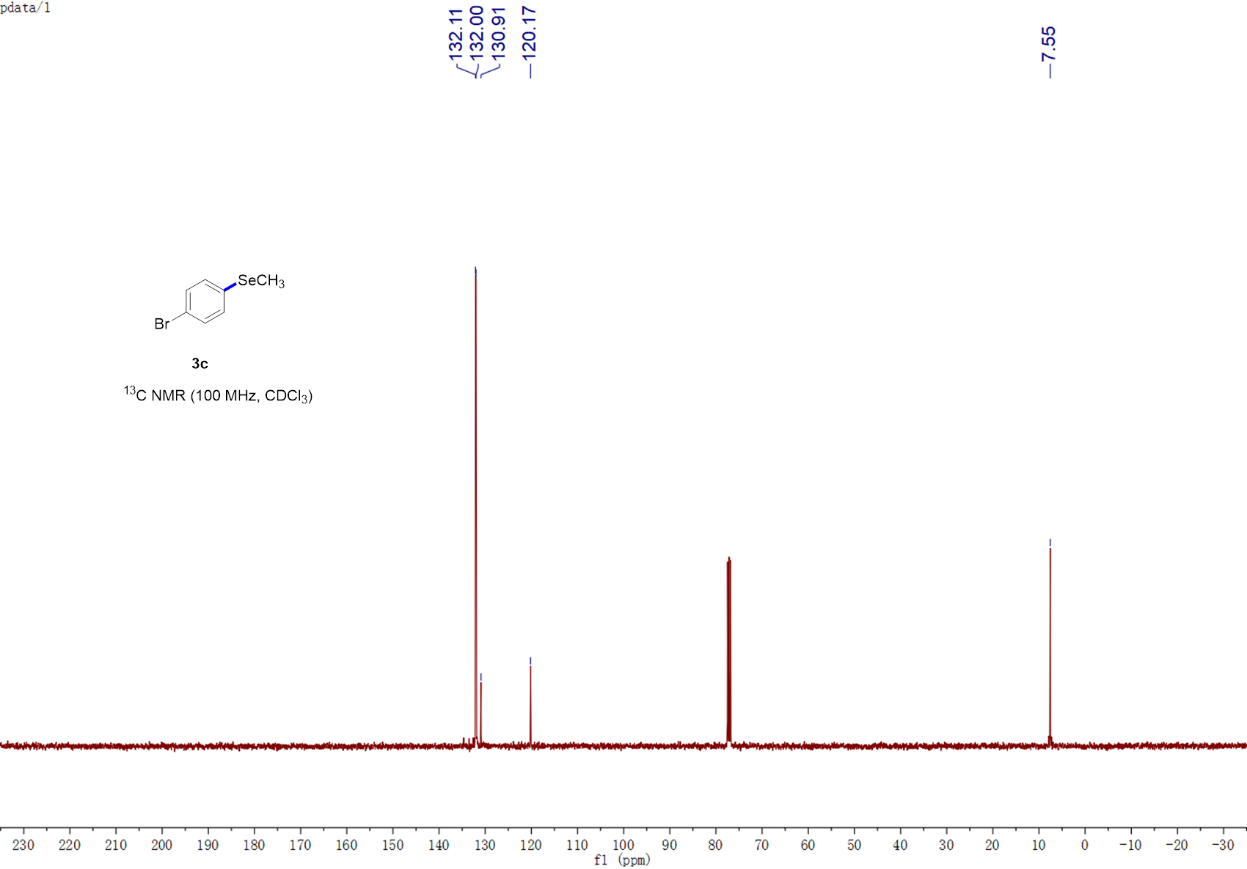
**

**^1^H NMR (400 MHz, Chloroform-*d*) spectrum of 3d**

**
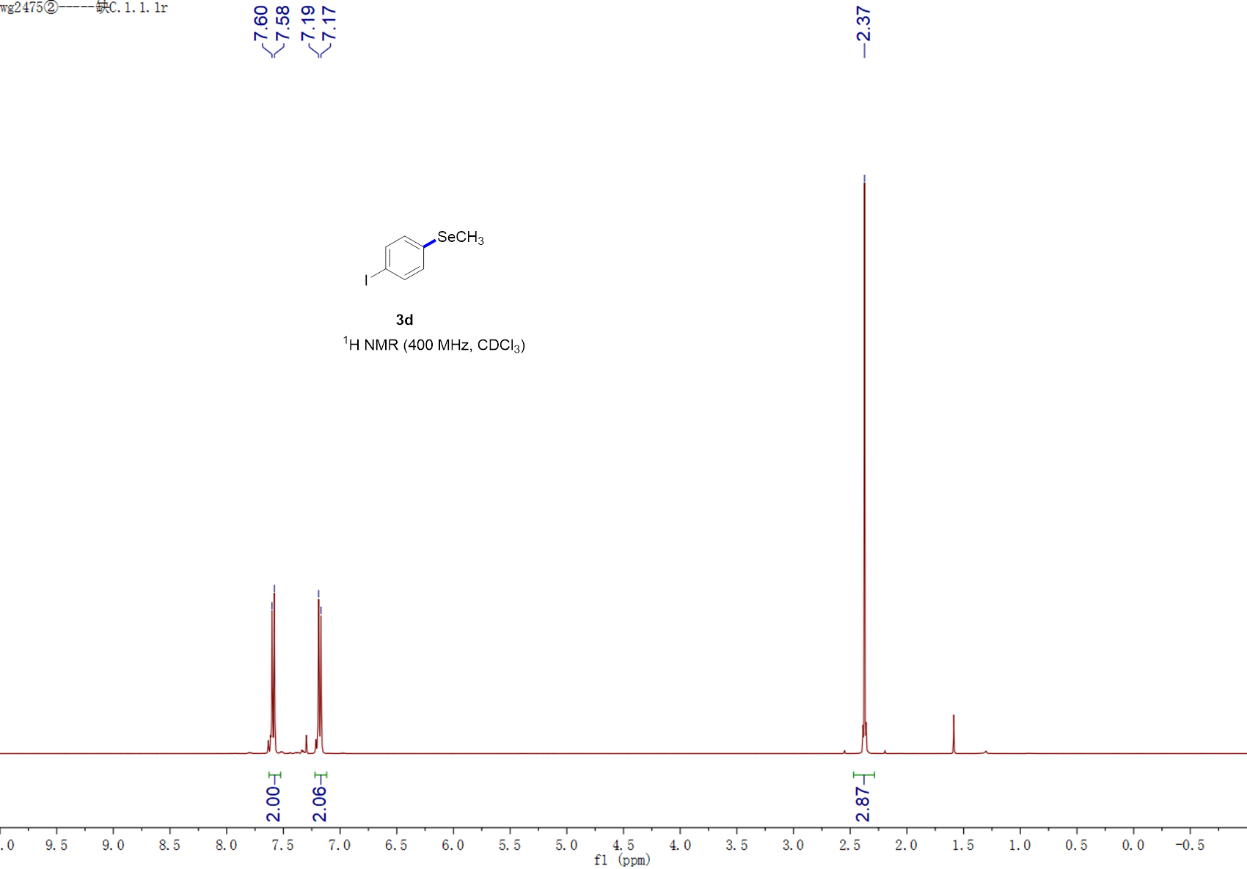
**

**^13^C{^1^H} NMR (100 MHz, Chloroform-*d*) spectrum of 3d**

**
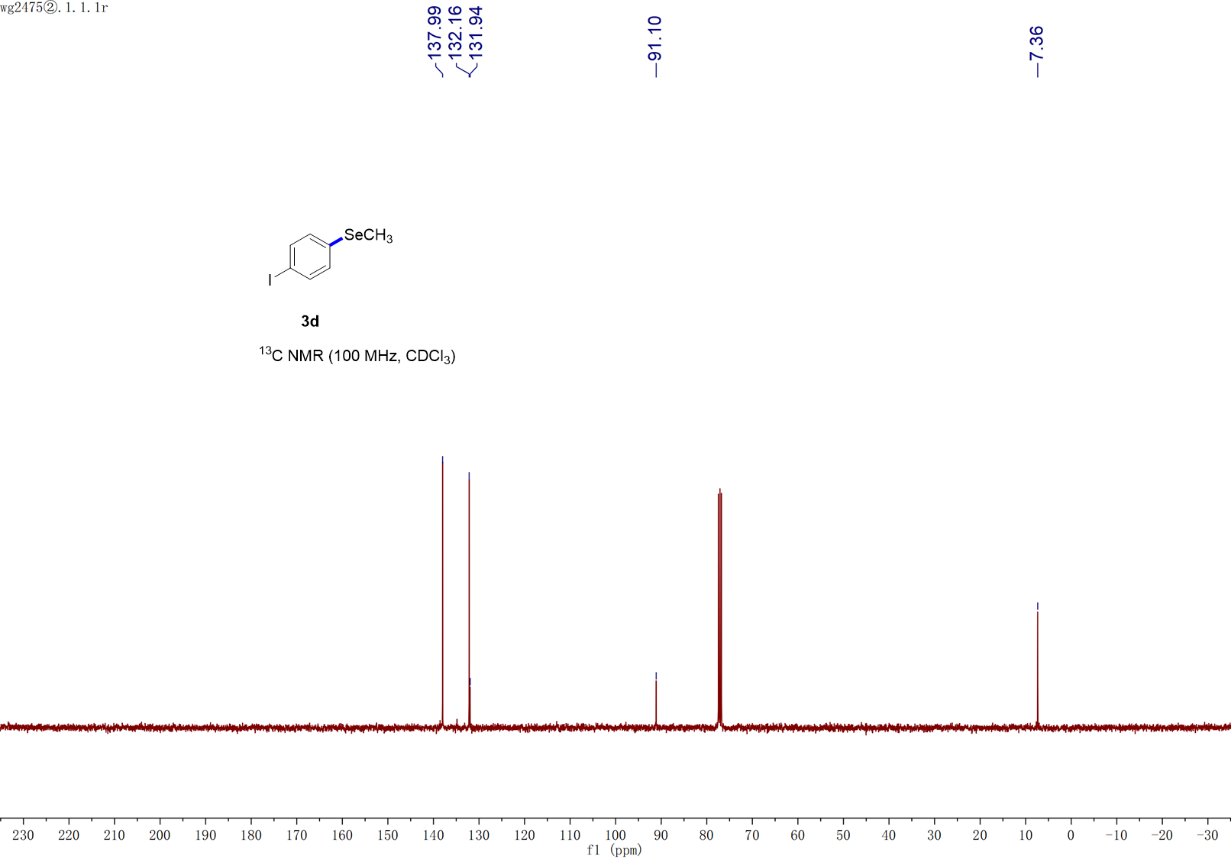
**

**^1^H NMR (400 MHz, Chloroform-*d*) spectrum of 3e**

**
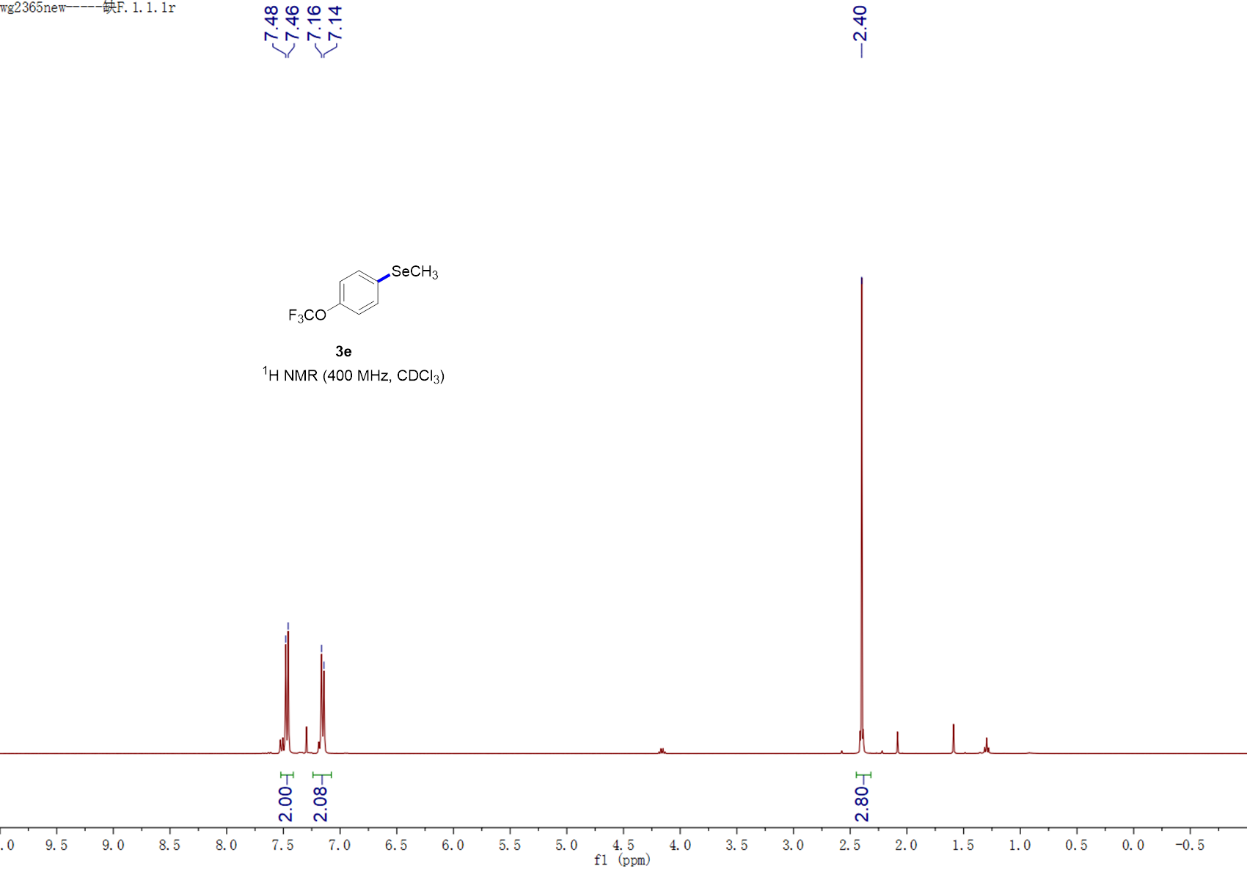
**

**^13^C{^1^H} NMR (100 MHz, Chloroform-*d*) spectrum of 3e**

**
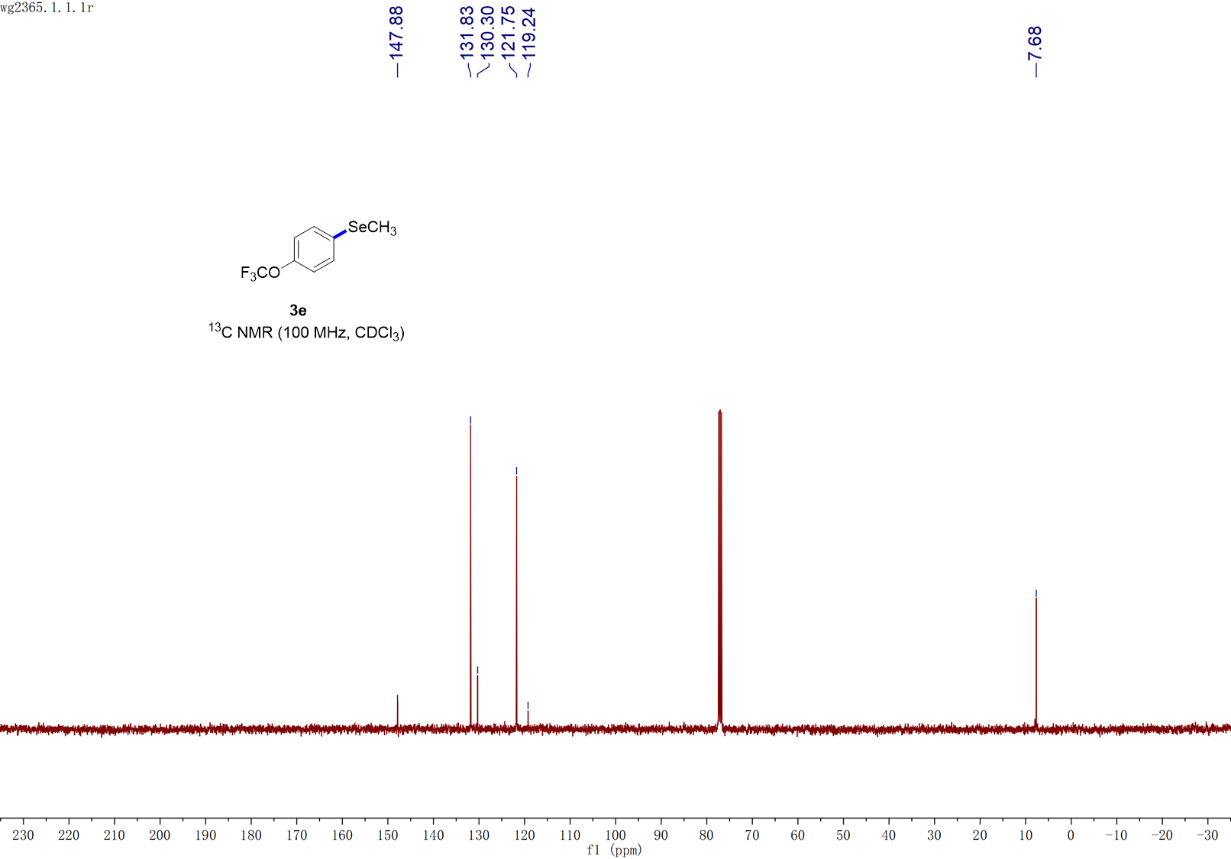
**

**^19^F NMR (375 MHz, Chloroform-*d*) spectrum of 3e**

**
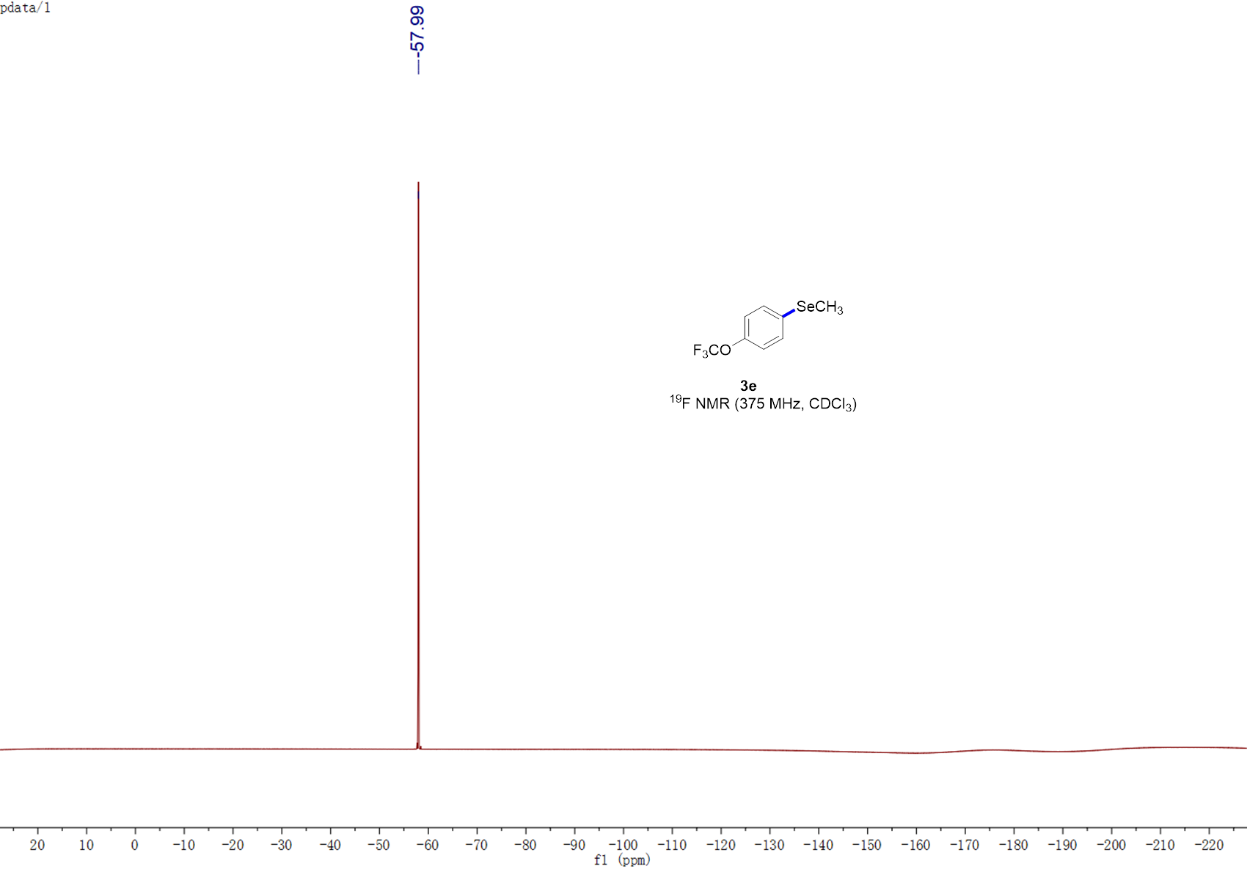
**

**^1^H NMR (400 MHz, Chloroform-*d*) spectrum of 3f**

**
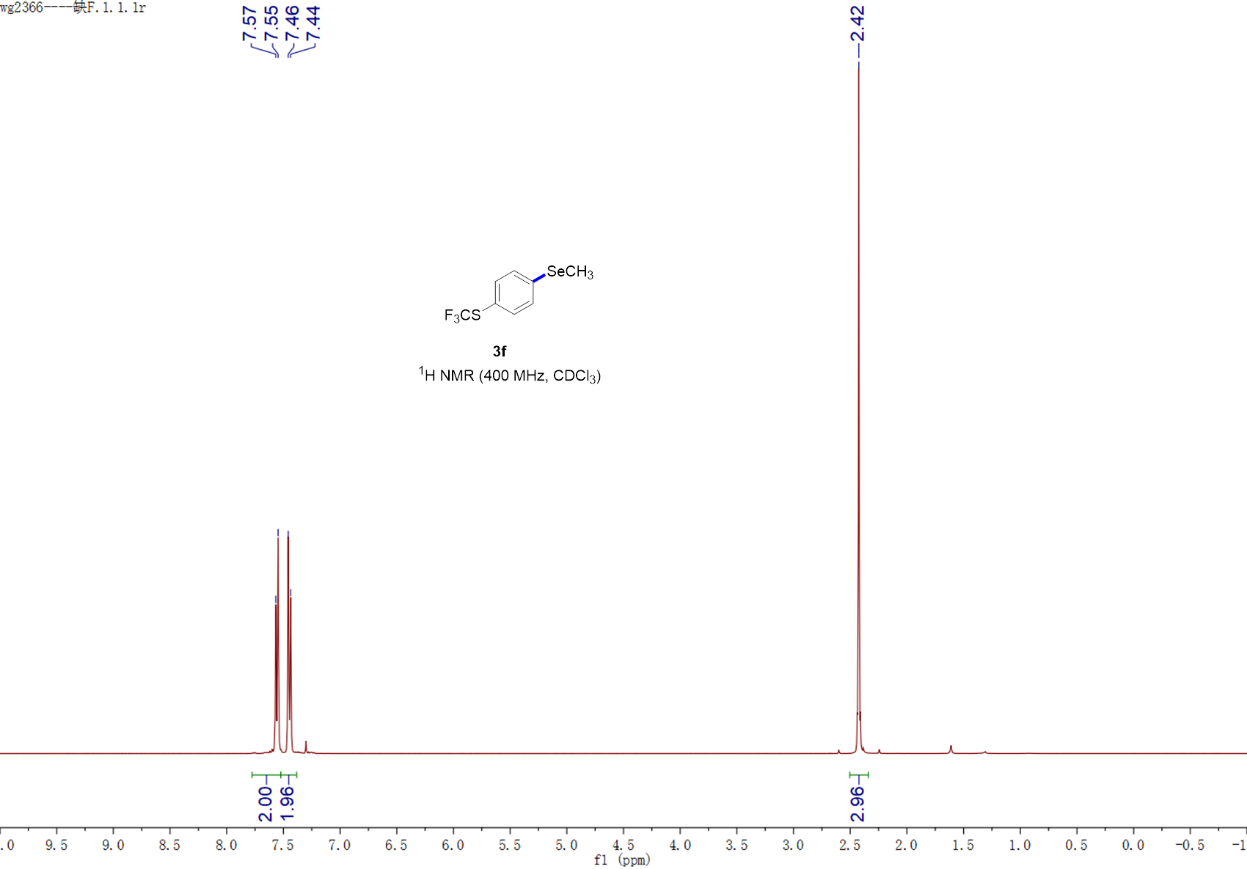
**

**^13^C{^1^H} NMR (100 MHz, Chloroform-*d*) spectrum of 3f**

**
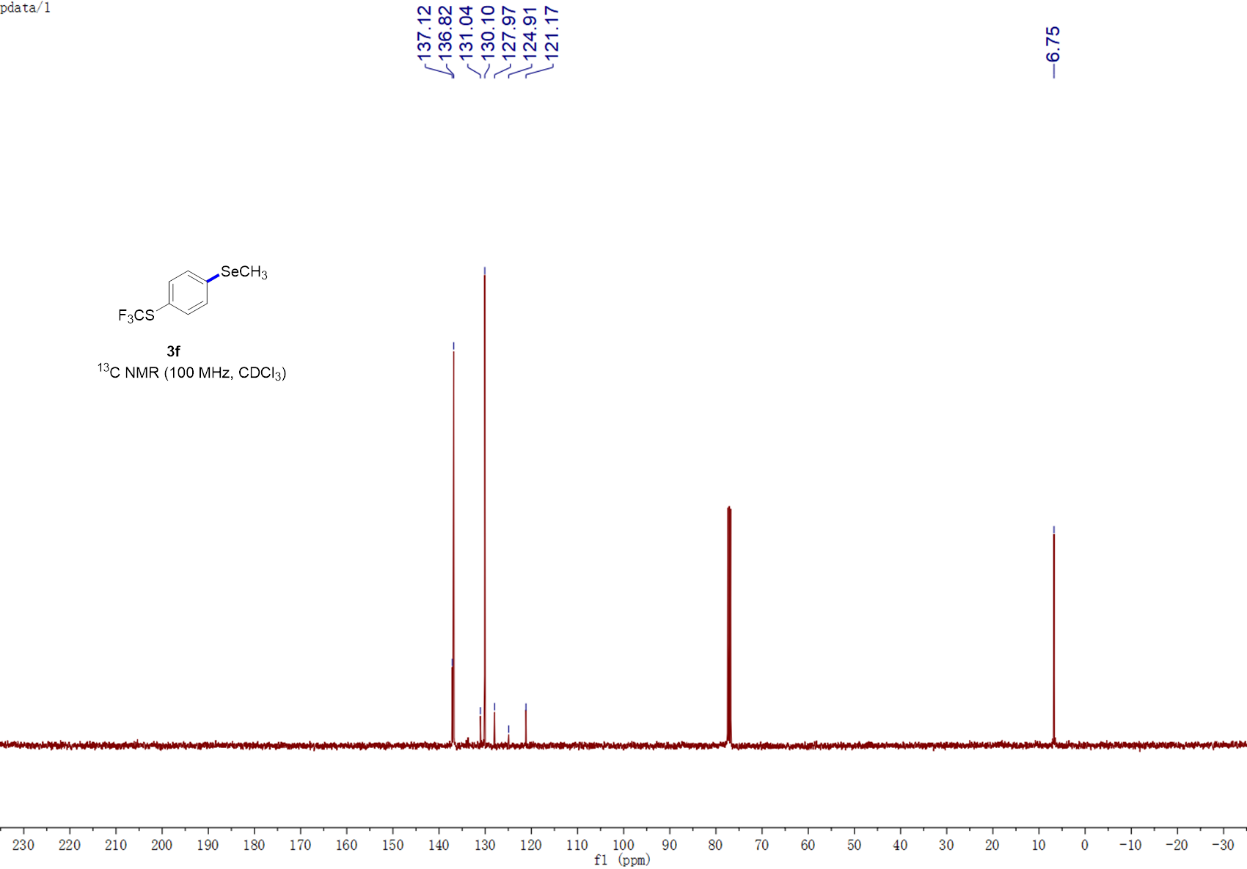
**

**^19^F NMR (375 MHz, Chloroform-*d*) spectrum of 3f**

**
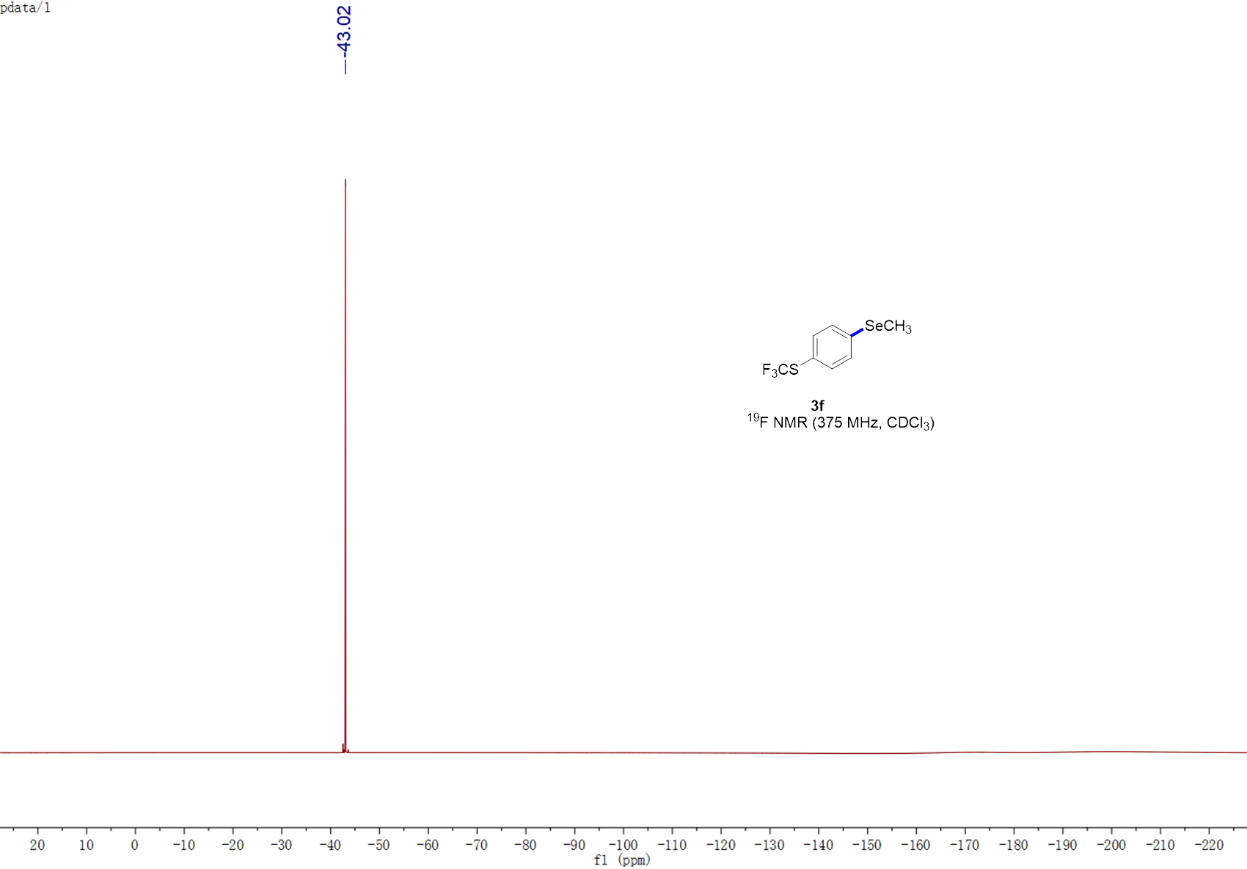
**

**^1^H NMR (400 MHz, Chloroform-*d*) spectrum of 3g**

**
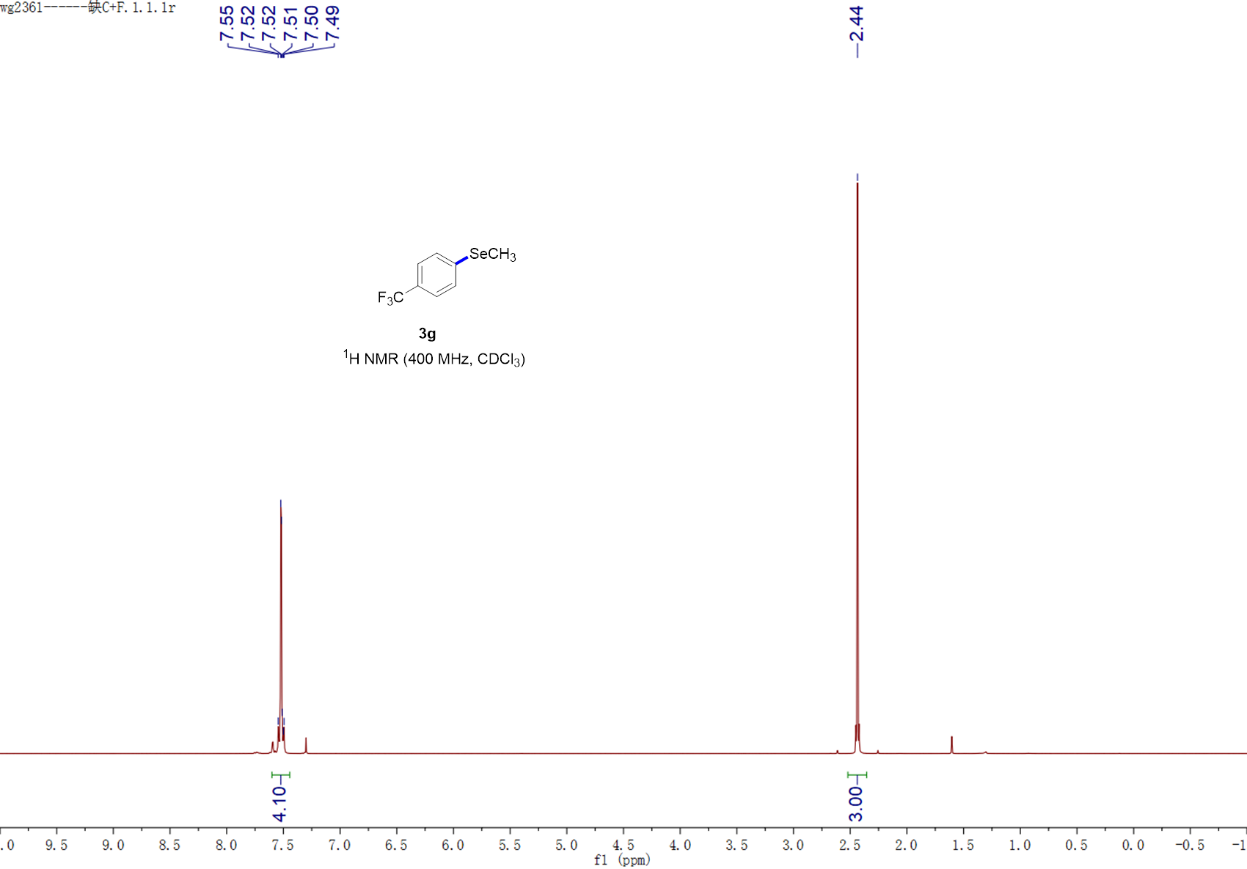
**

**^13^C{^1^H} NMR (100 MHz, Chloroform-*d*) spectrum of 3g**

**
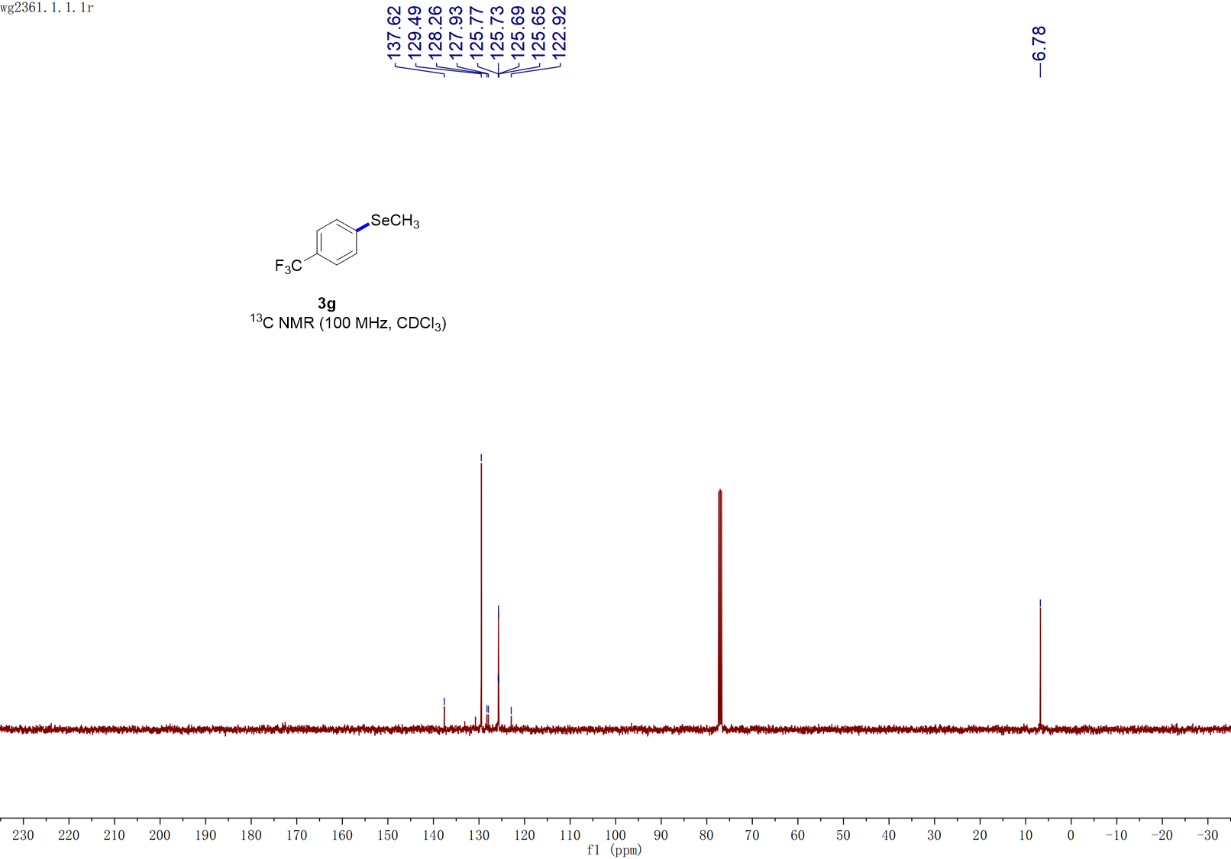
**

**^19^F NMR (375 MHz, Chloroform-*d*) spectrum of 3g**

**
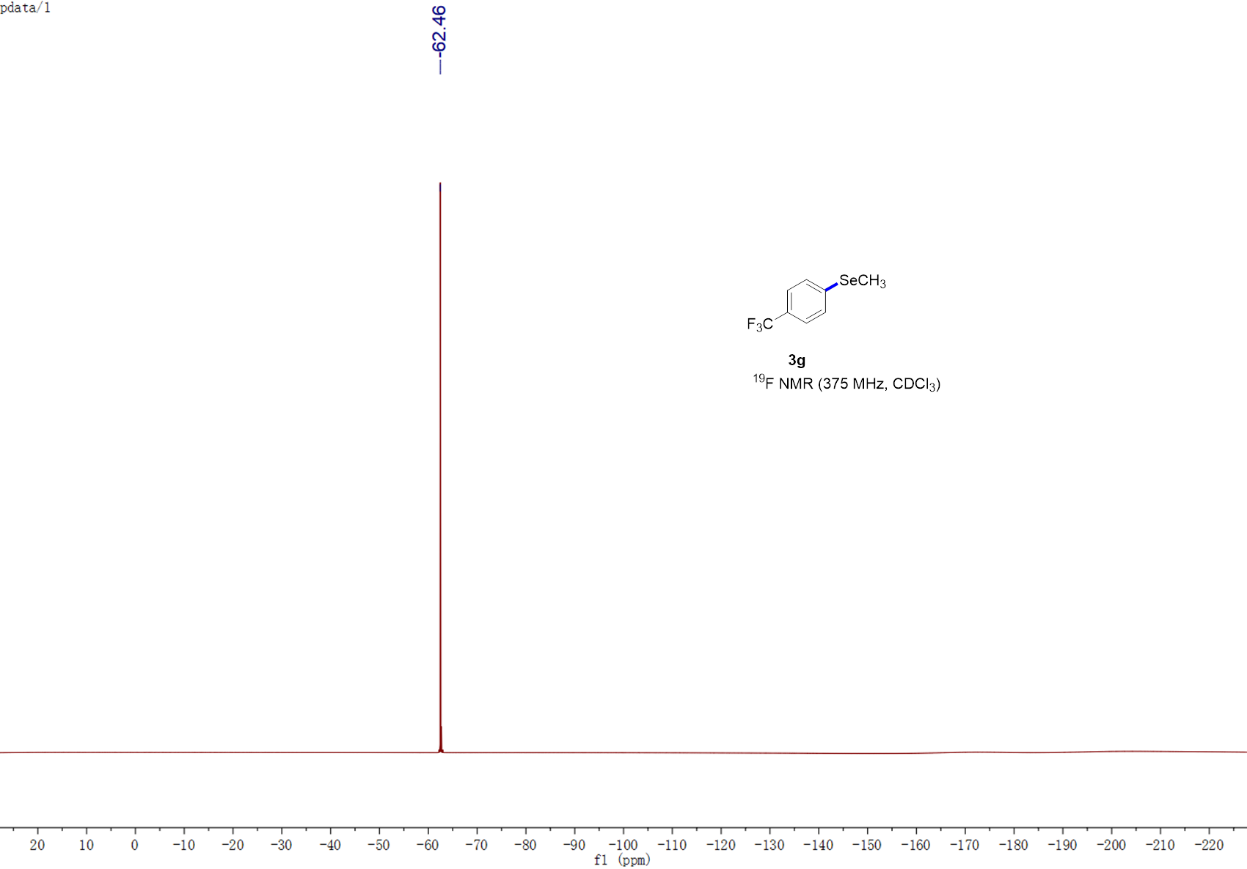
**

**^1^H NMR (400 MHz, Chloroform-*d*) spectrum of 3h**

**
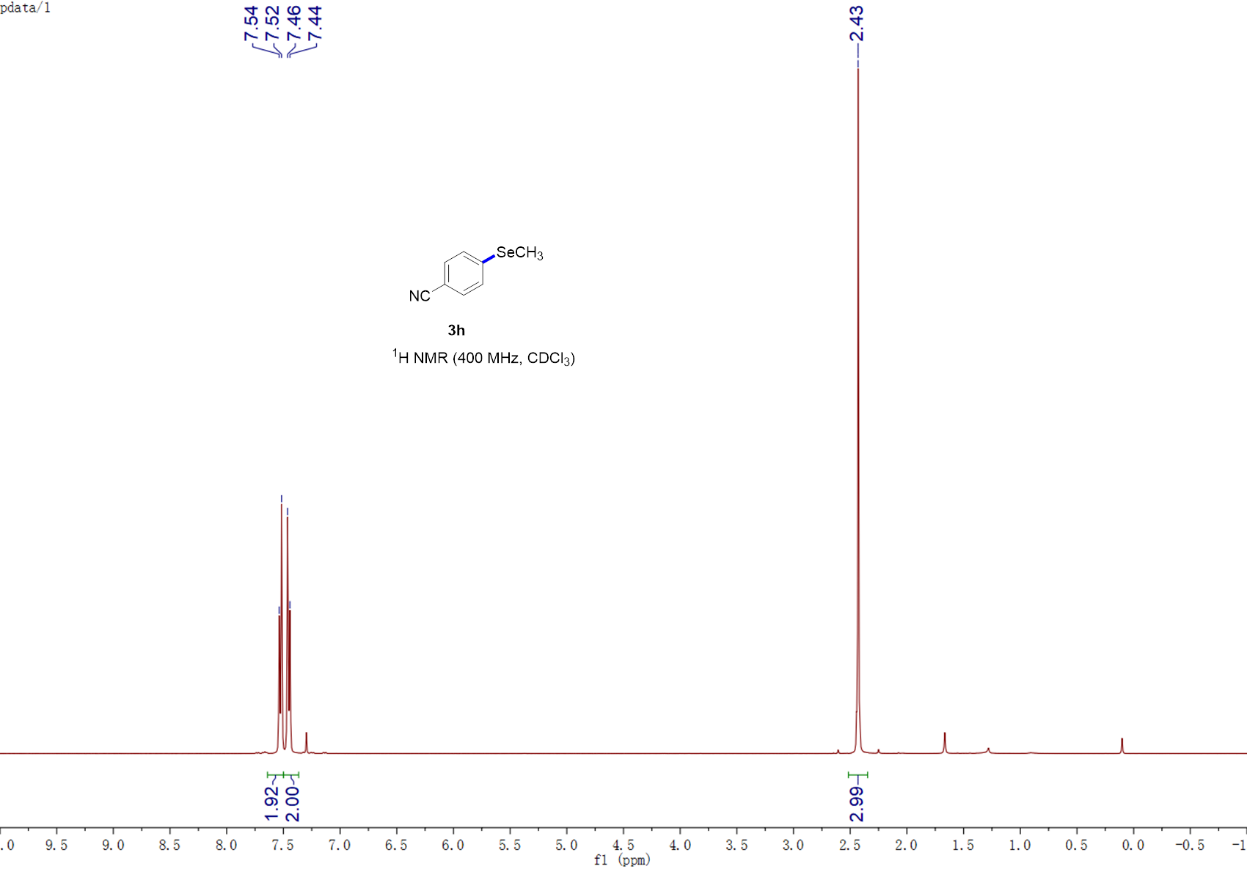
**

**^13^C{^1^H} NMR (100 MHz, Chloroform-*d*) spectrum of 3h**

**
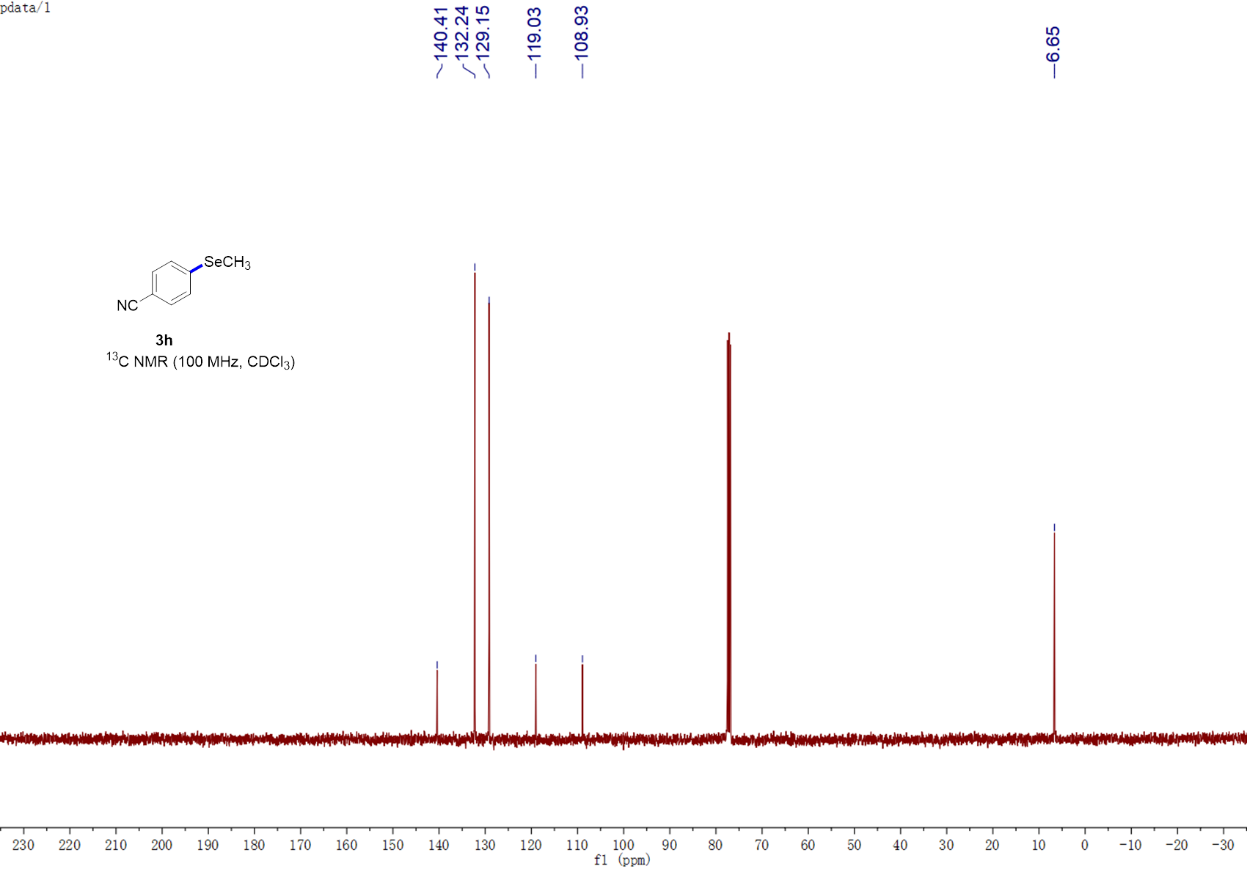
**

**^1^H NMR (400 MHz, Chloroform-*d*) spectrum of 3i**

**
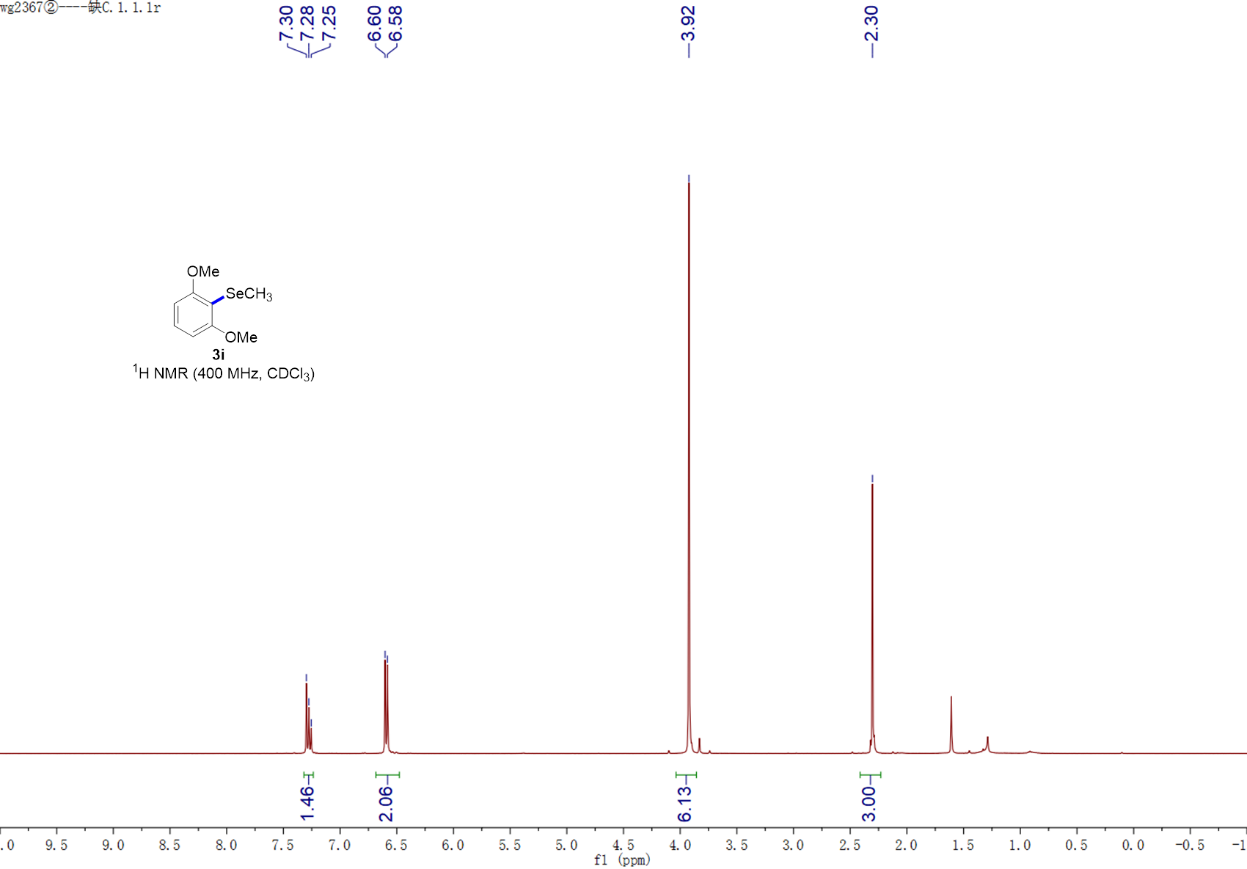
**

**^13^C{^1^H} NMR (100 MHz, Chloroform-*d*) spectrum of 3i**

**
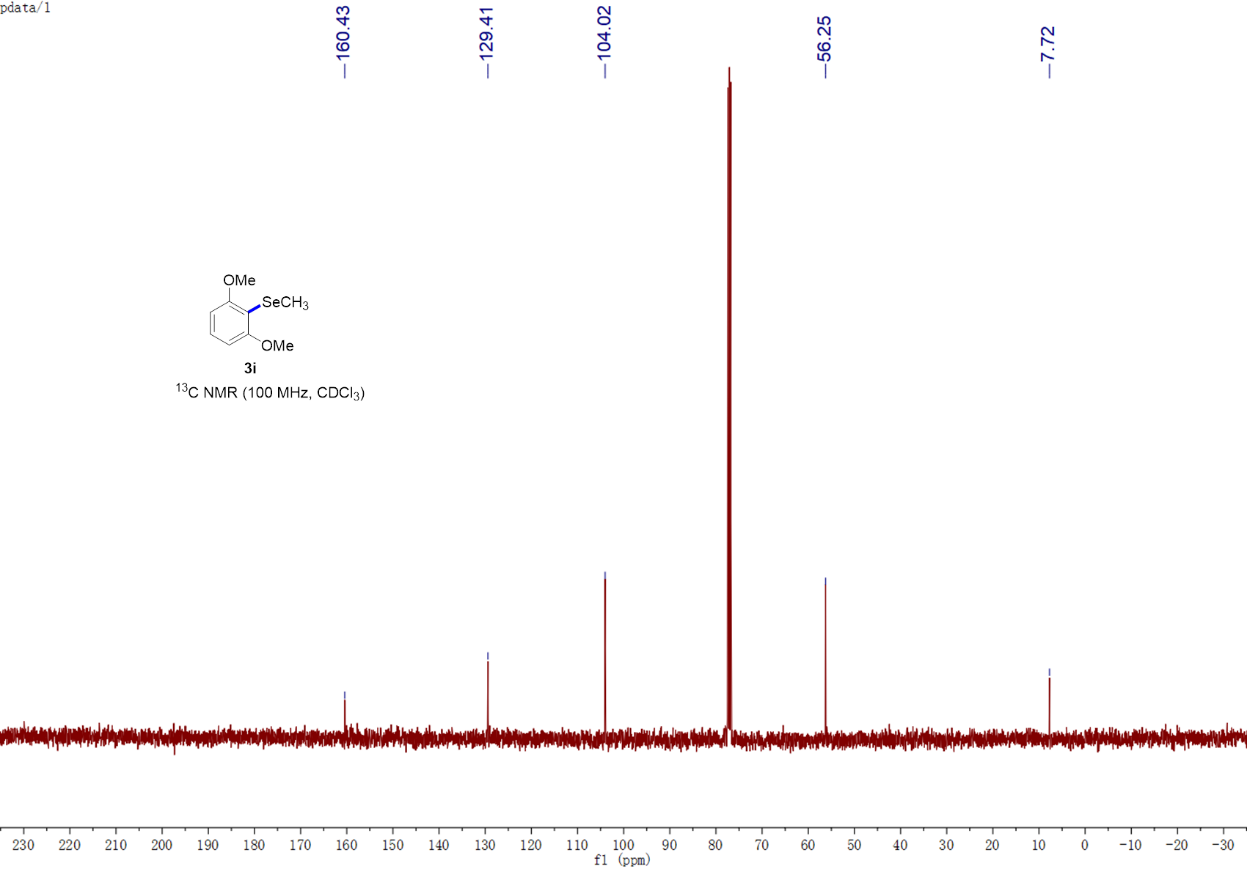
**

**^1^H NMR (400 MHz, Chloroform-*d*) spectrum of 3j**

**
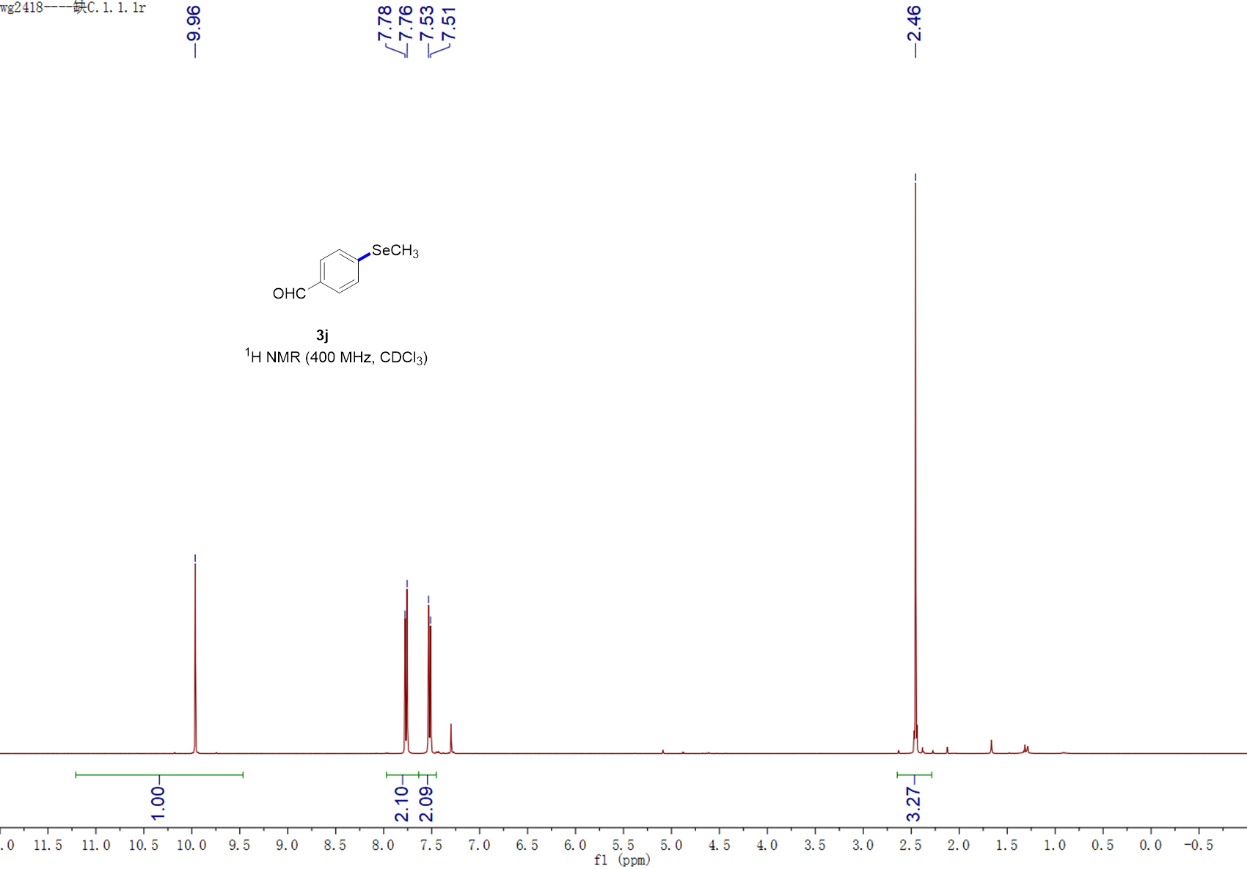
**

**^13^C{^1^H} NMR (100 MHz, Chloroform-*d*) spectrum of 3j**

**
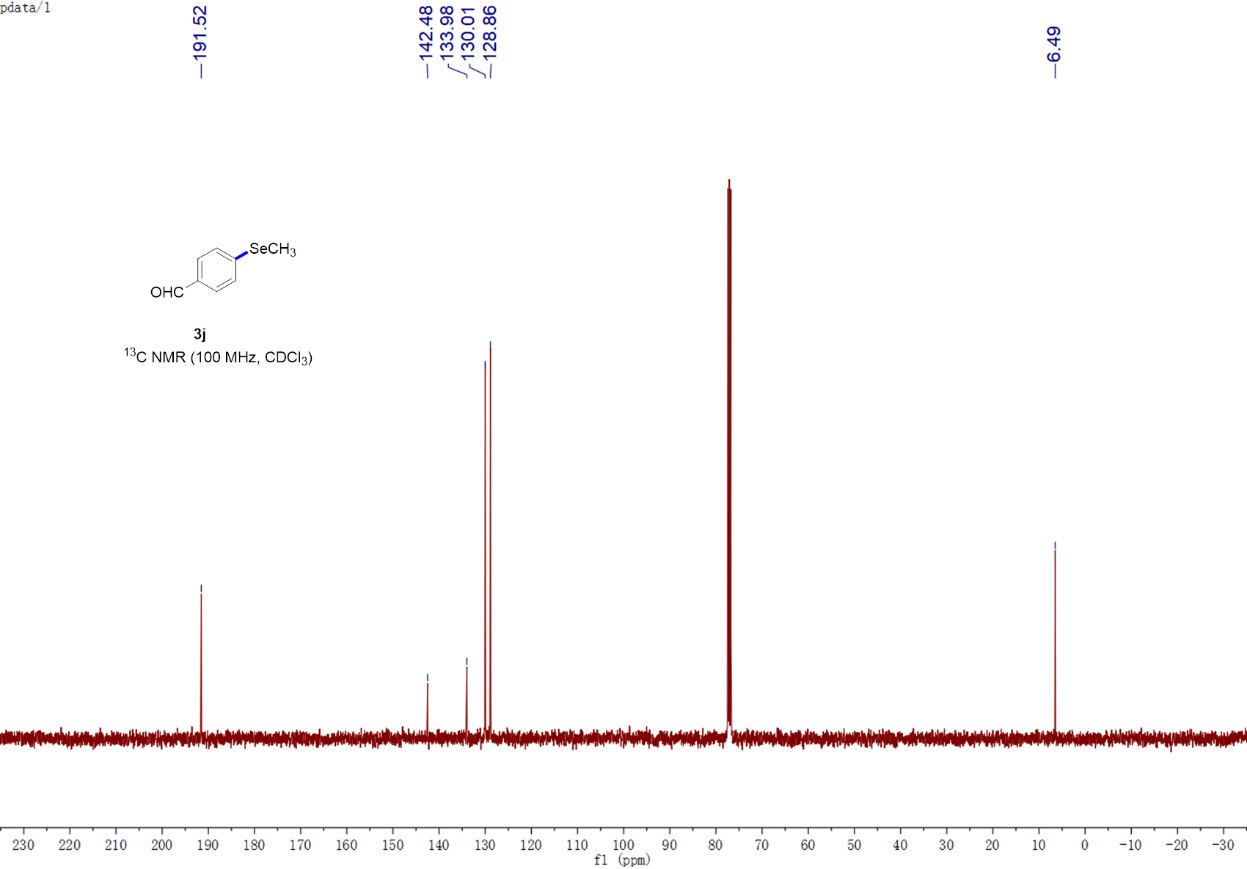
**

**^1^H NMR (400 MHz, Chloroform-*d*) spectrum of 3k**

**
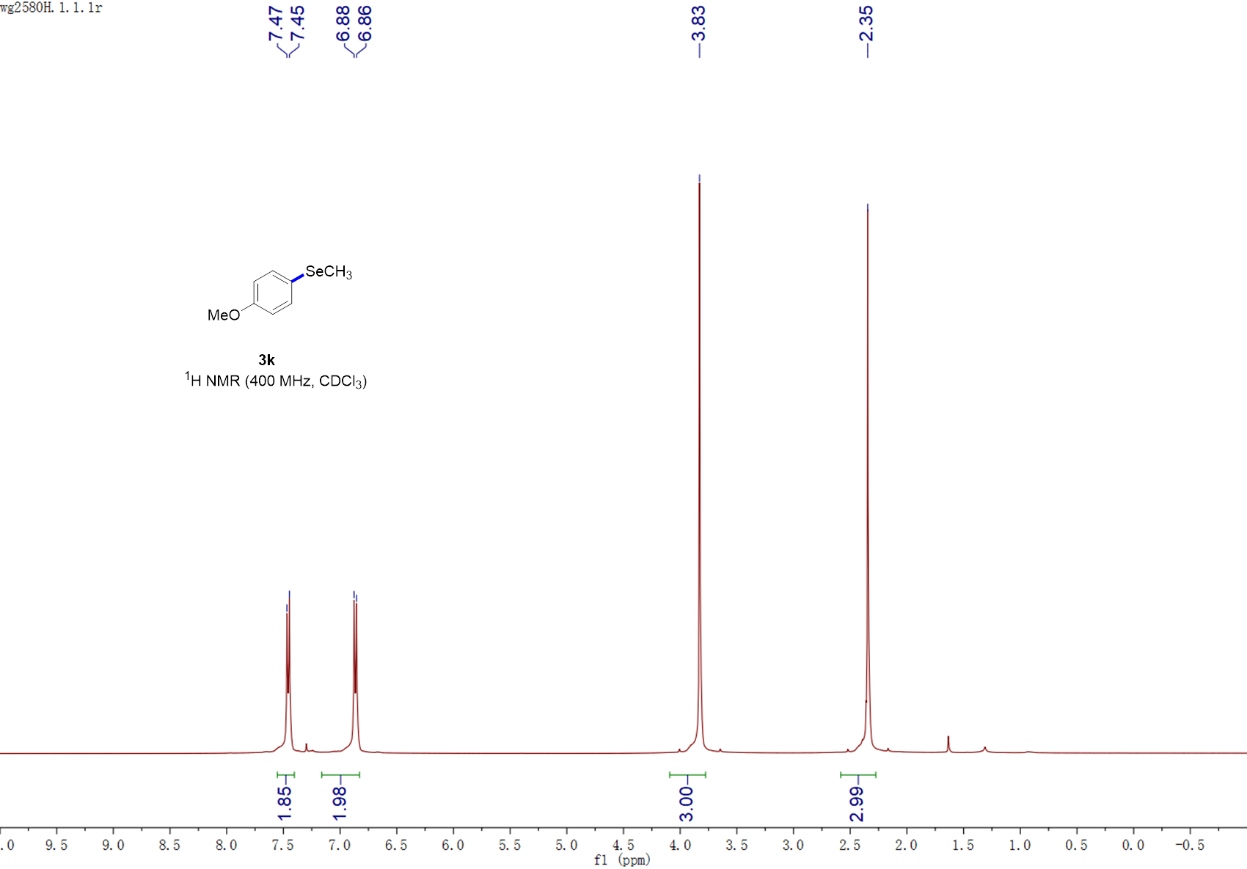
**

**^13^C{^1^H} NMR (100 MHz, Chloroform-*d*) spectrum of 3k**

**
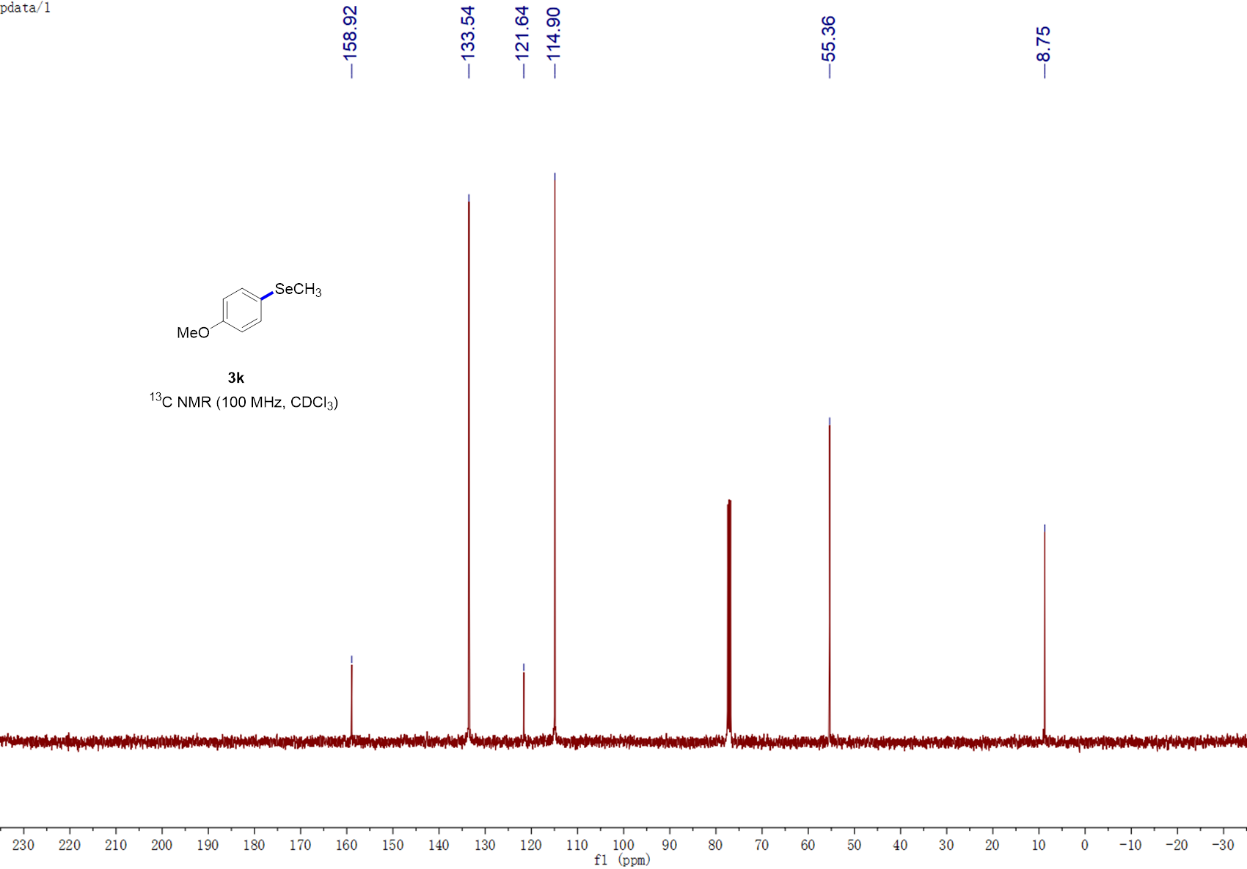
**

**^1^H NMR (400 MHz, Chloroform-*d*) spectrum of 3l**

**
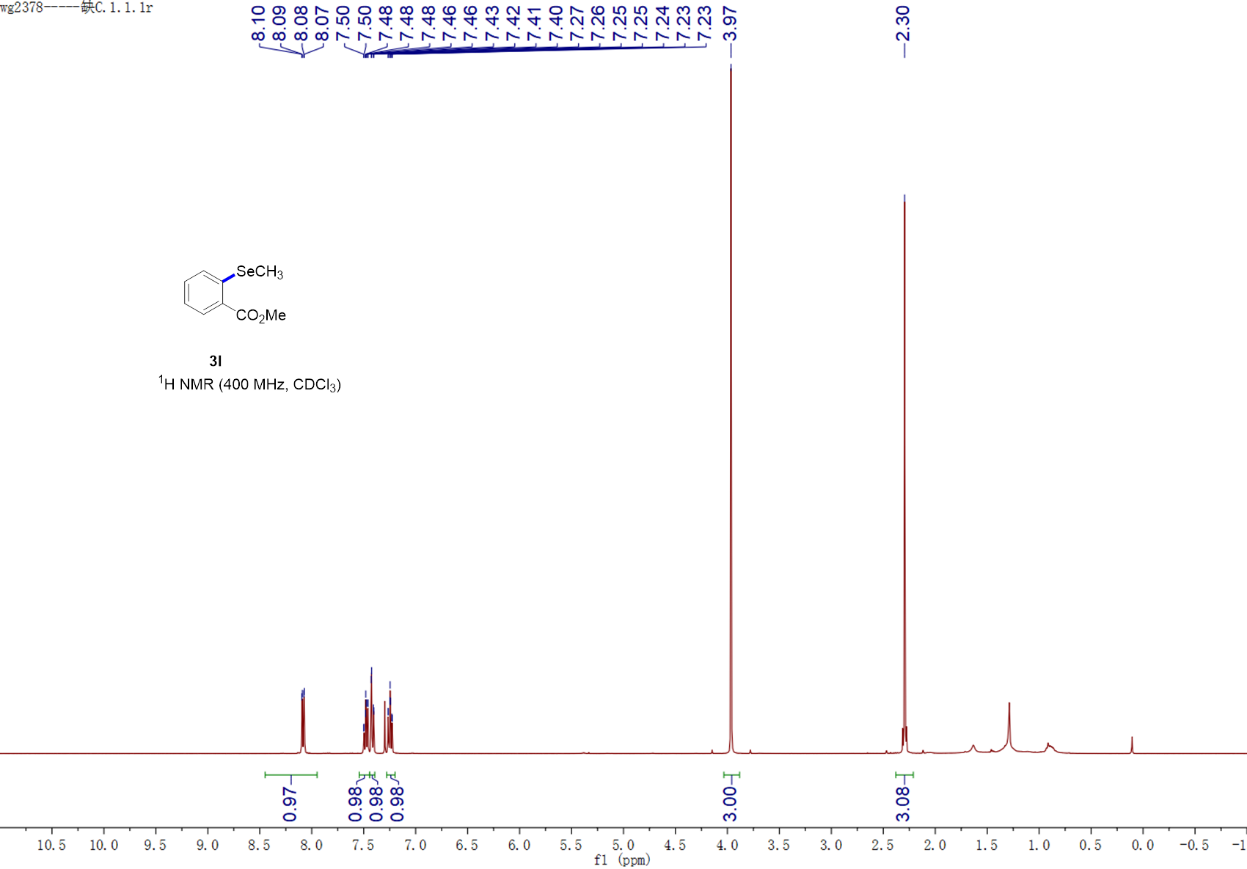
**

**^13^C{^1^H} NMR (100 MHz, Chloroform-*d*) spectrum of 3l**

**
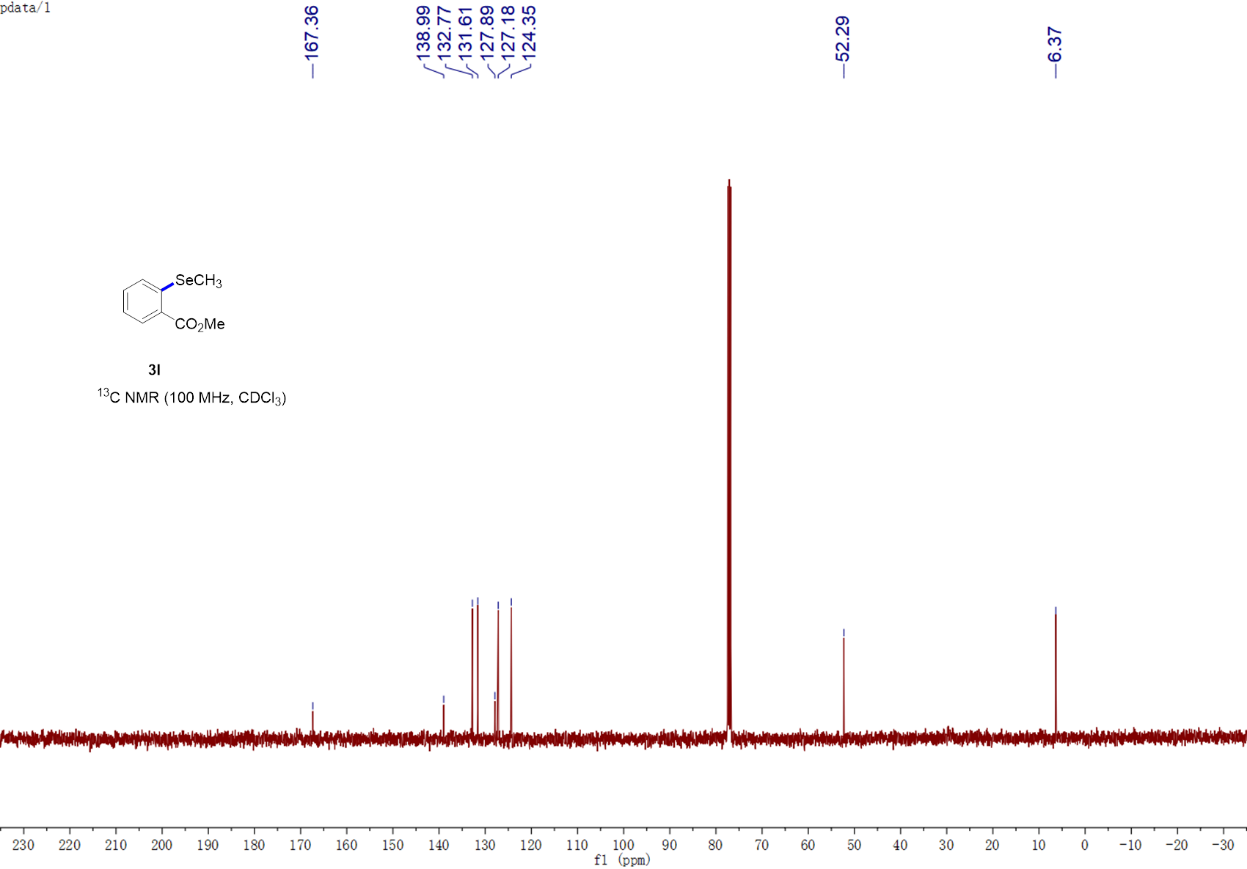
**

**^1^H NMR (400 MHz, Chloroform-*d*) spectrum of 3m**

**
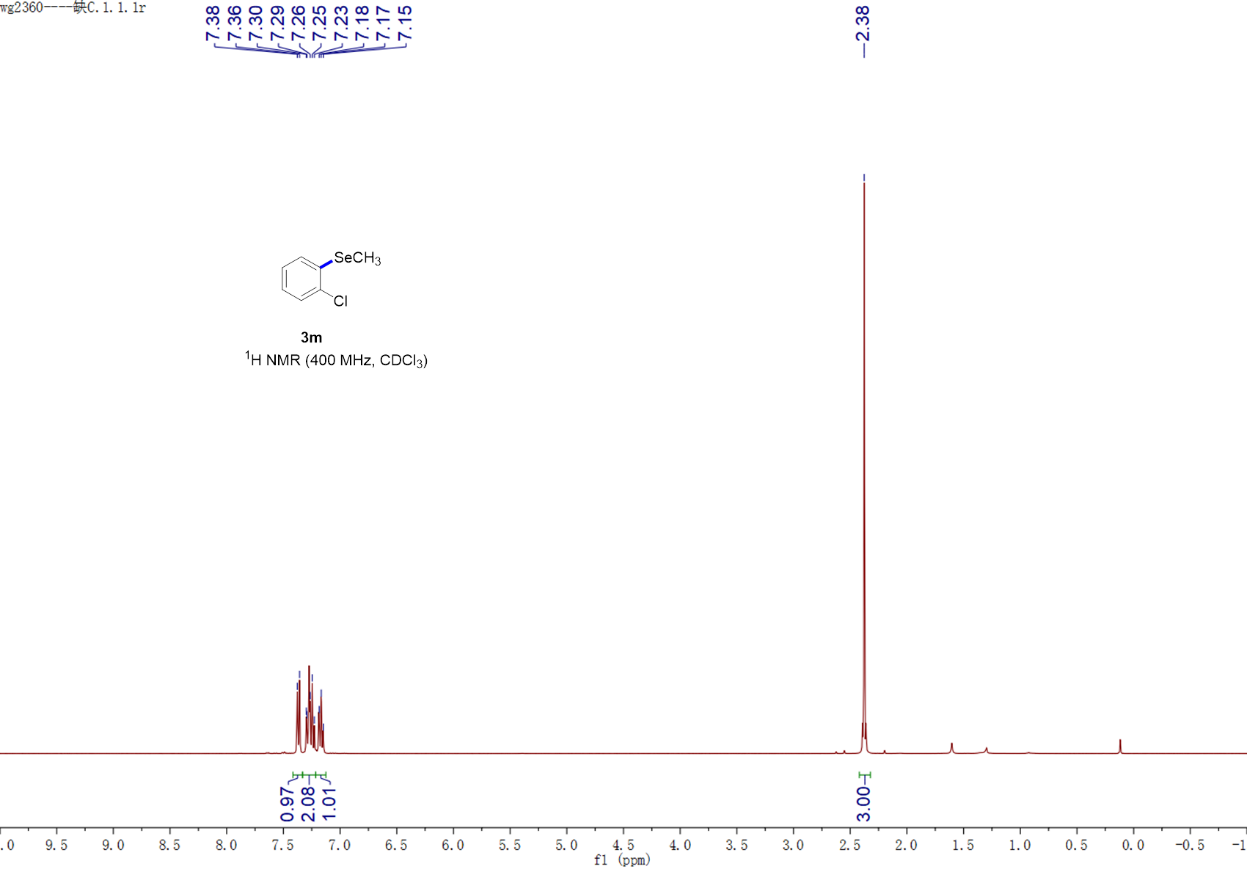
**

**^13^C{^1^H} NMR (100 MHz, Chloroform-*d*) spectrum of 3m**

**
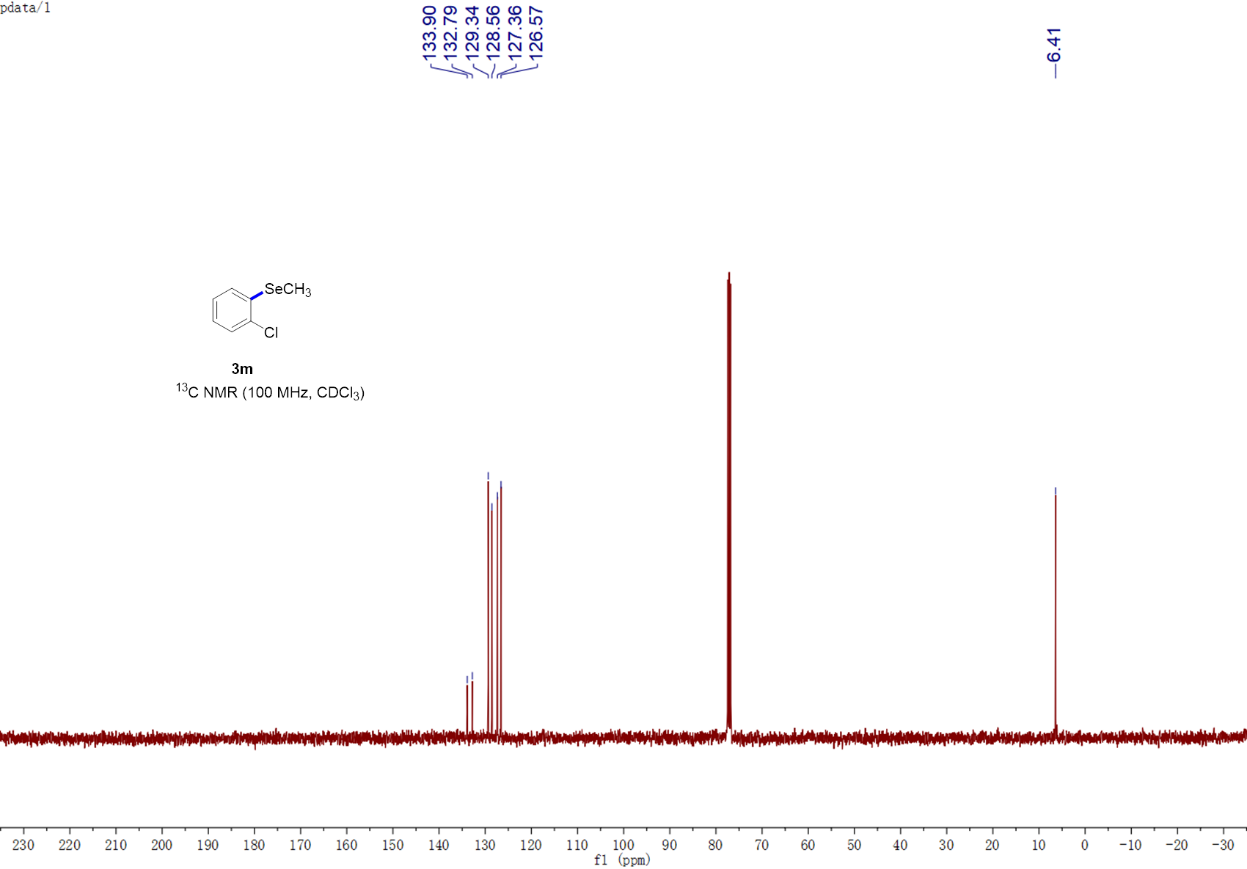
**

**^1^H NMR (400 MHz, Chloroform-*d*) spectrum of 3n**

**
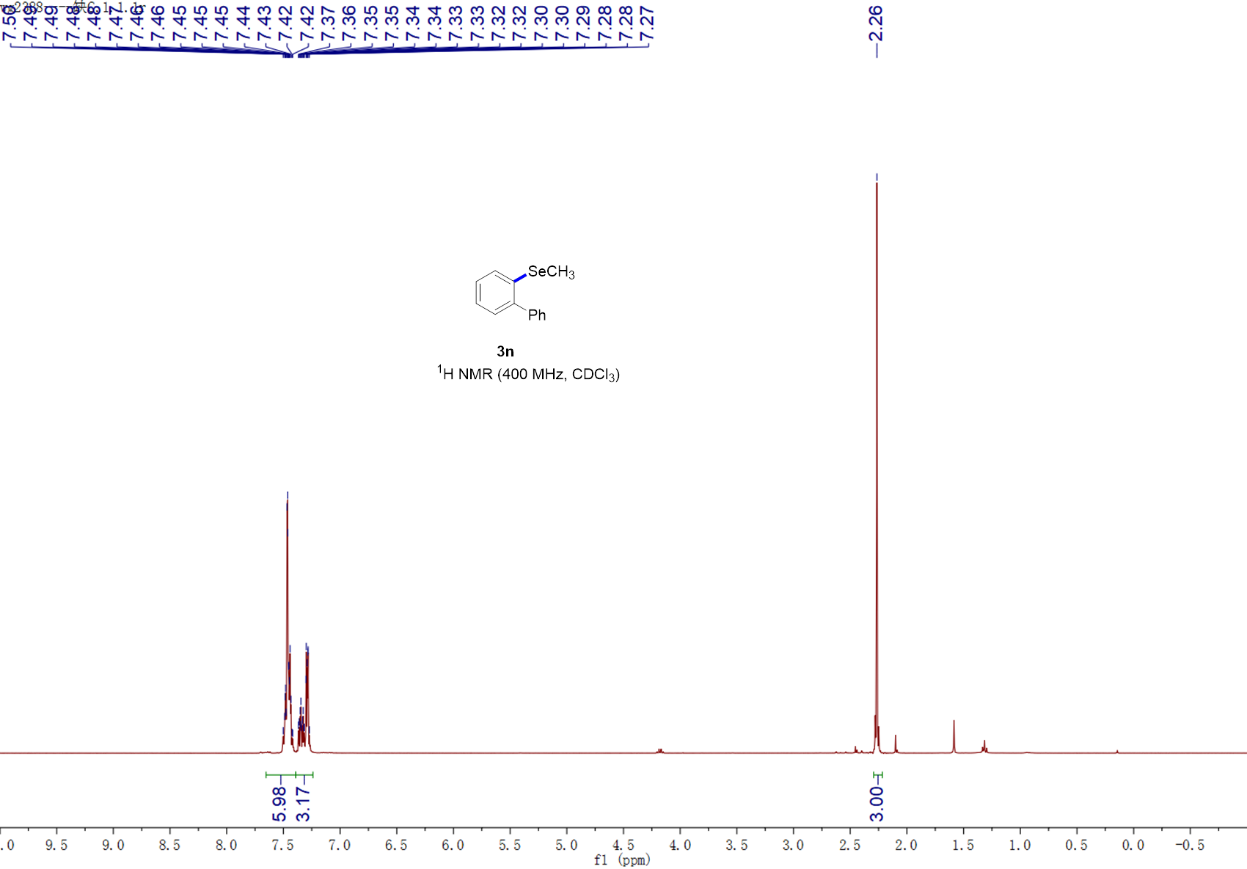
**

**^13^C{^1^H} NMR (100 MHz, Chloroform-*d*) spectrum of 3n**

**
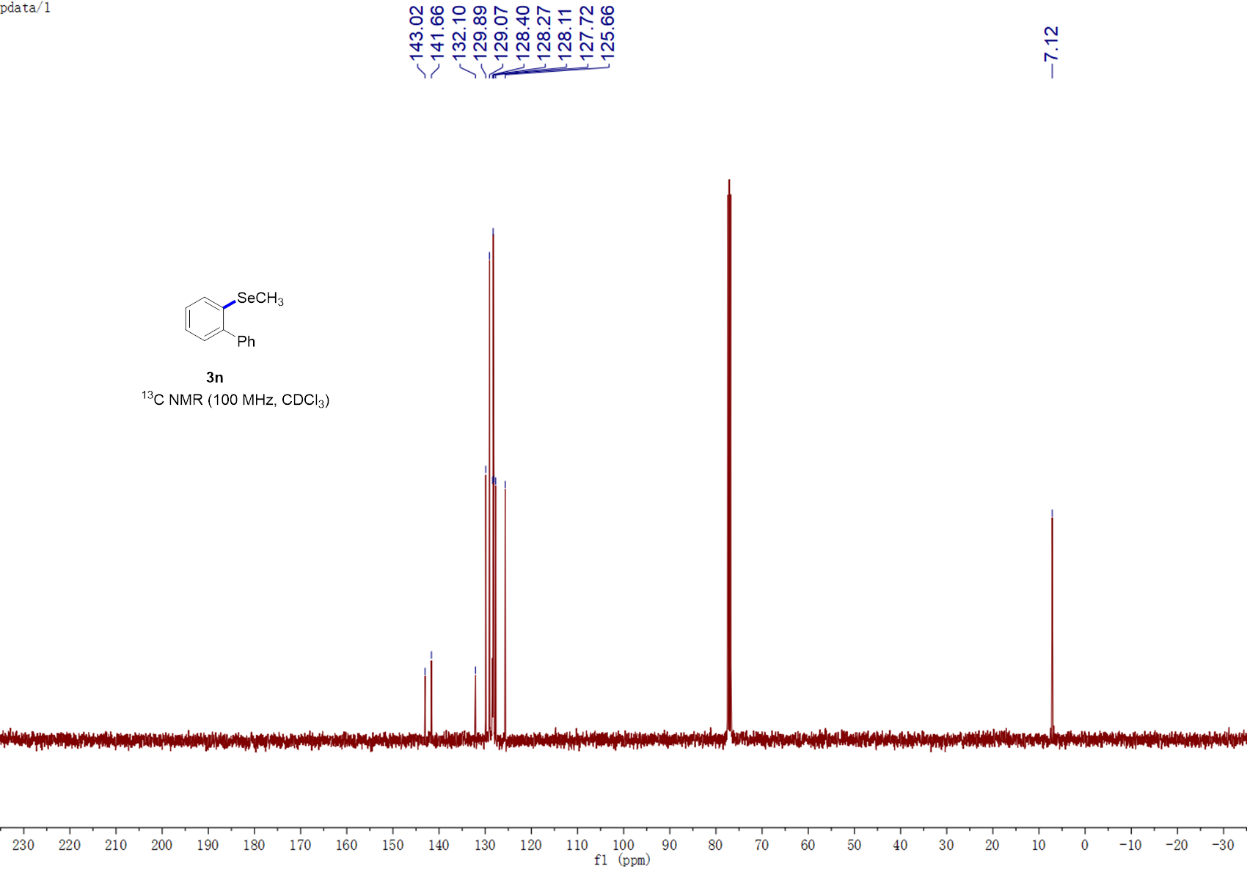
**

**^1^H NMR (400 MHz, Chloroform-*d*) spectrum of 3o**

**
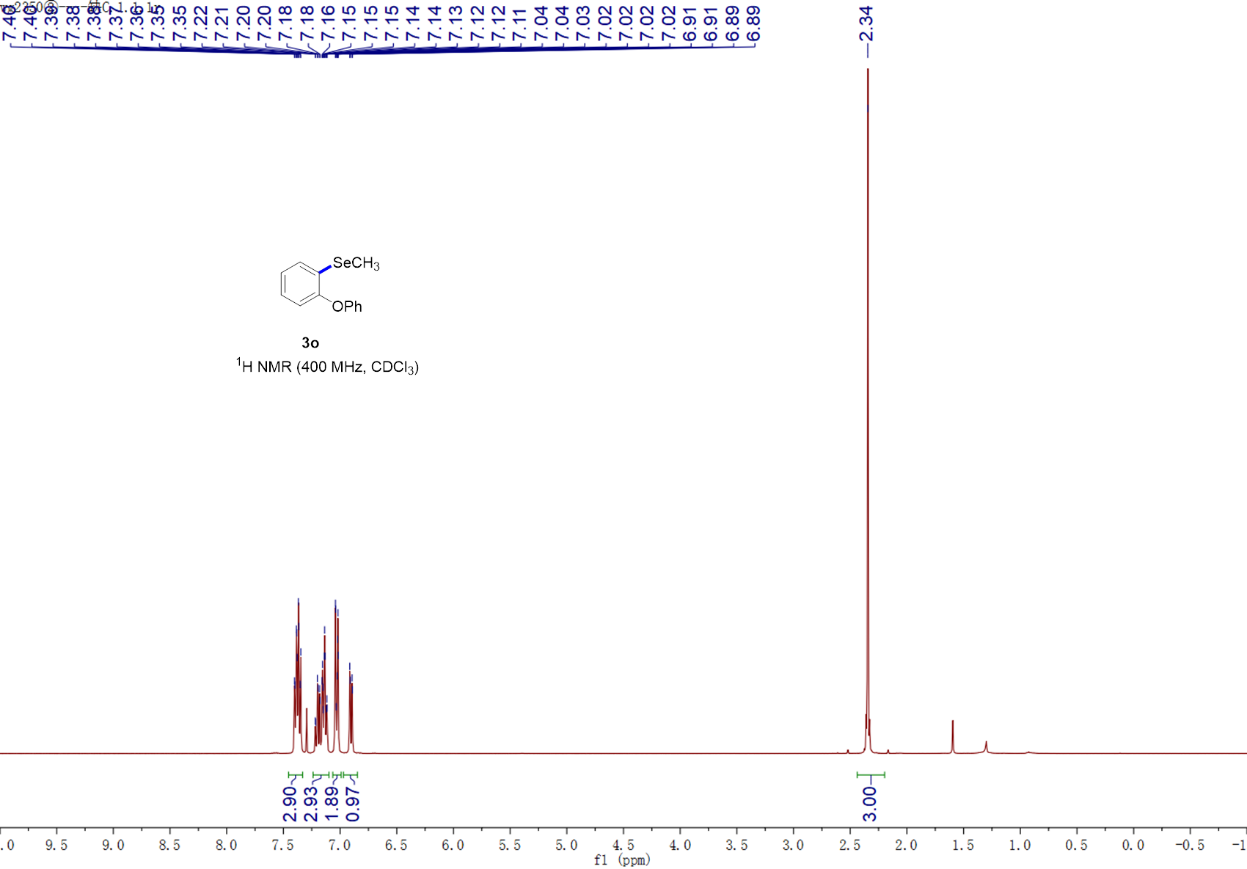
**

**^13^C{^1^H} NMR (100 MHz, Chloroform-*d*) spectrum of 3o**

**
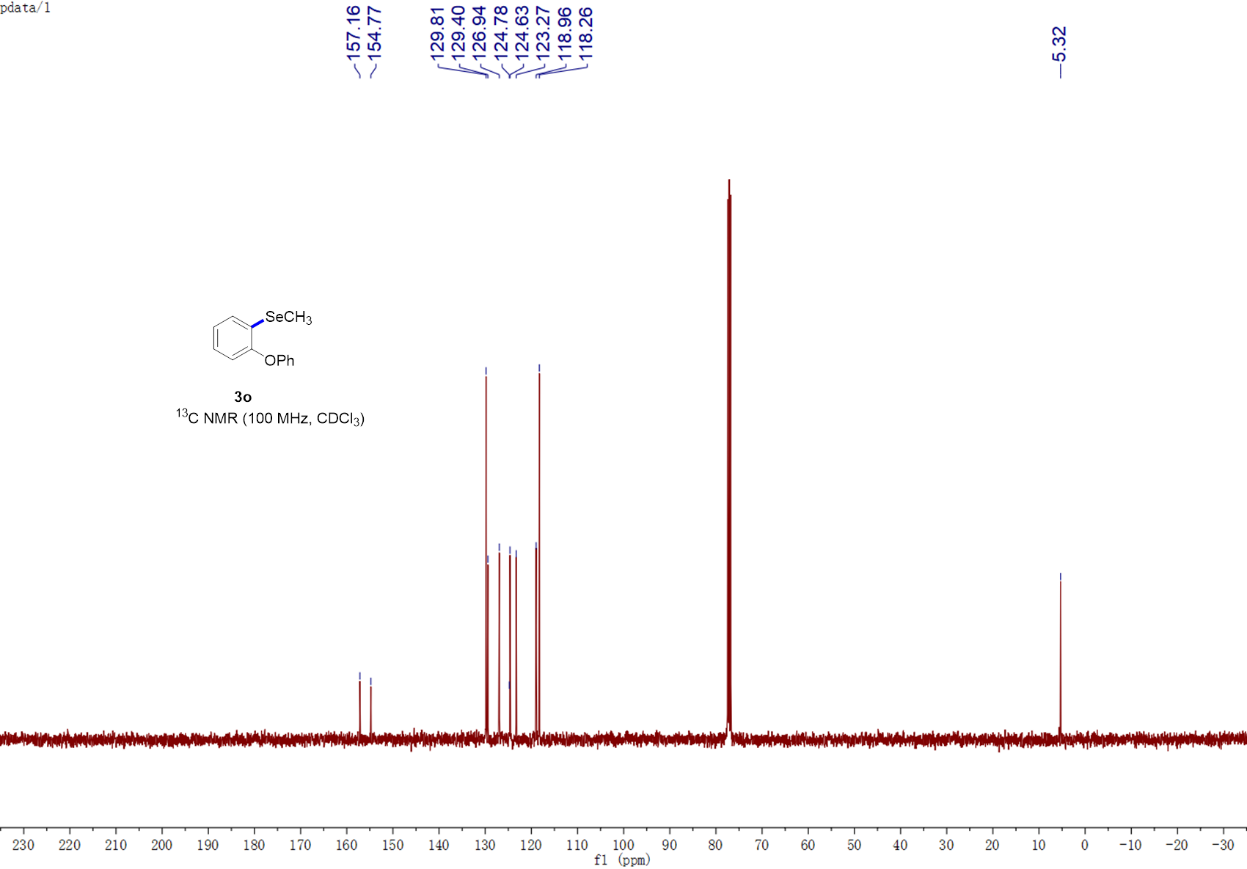
**

**^1^H NMR (400 MHz, Chloroform-*d*) spectrum of 3p**

**
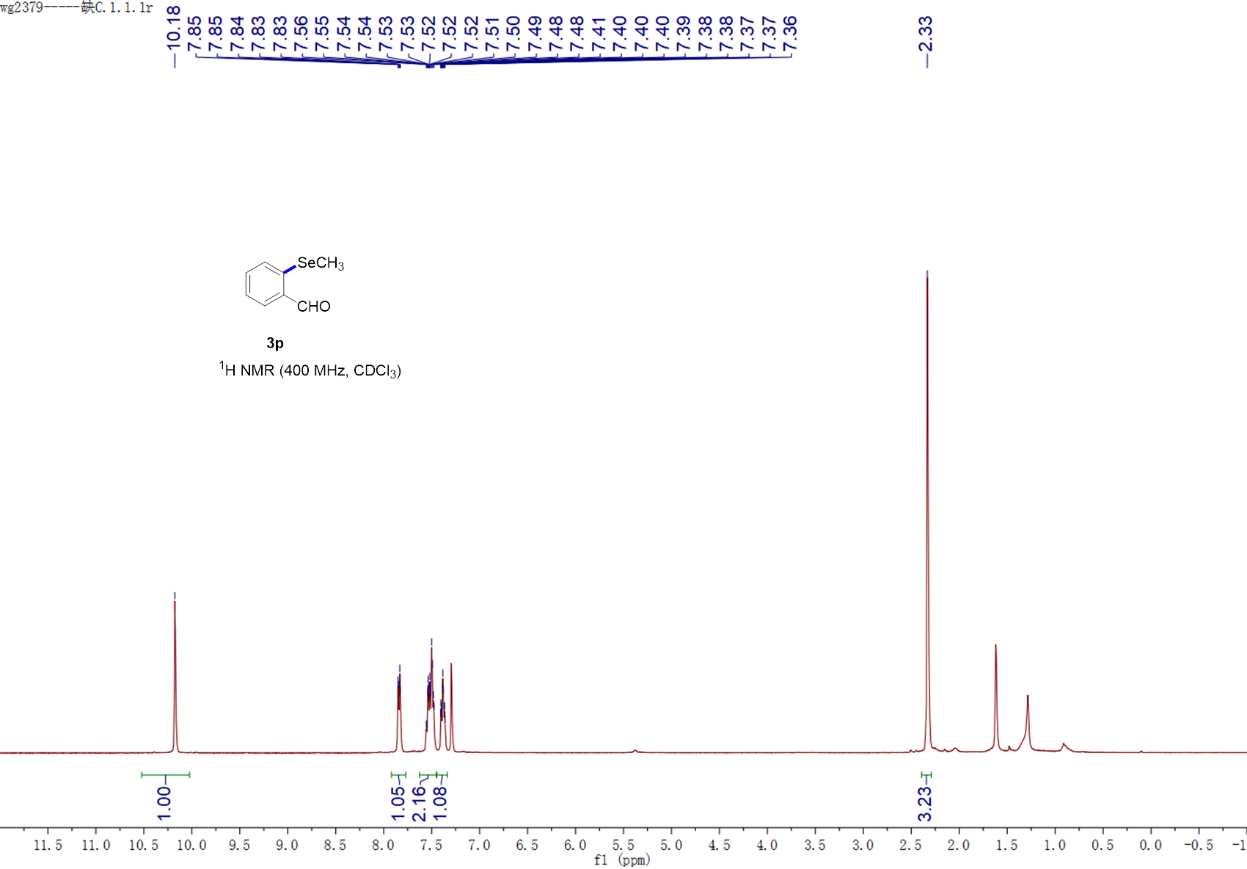
**

**^13^C{^1^H} NMR (100 MHz, Chloroform-*d*) spectrum of 3p**

**
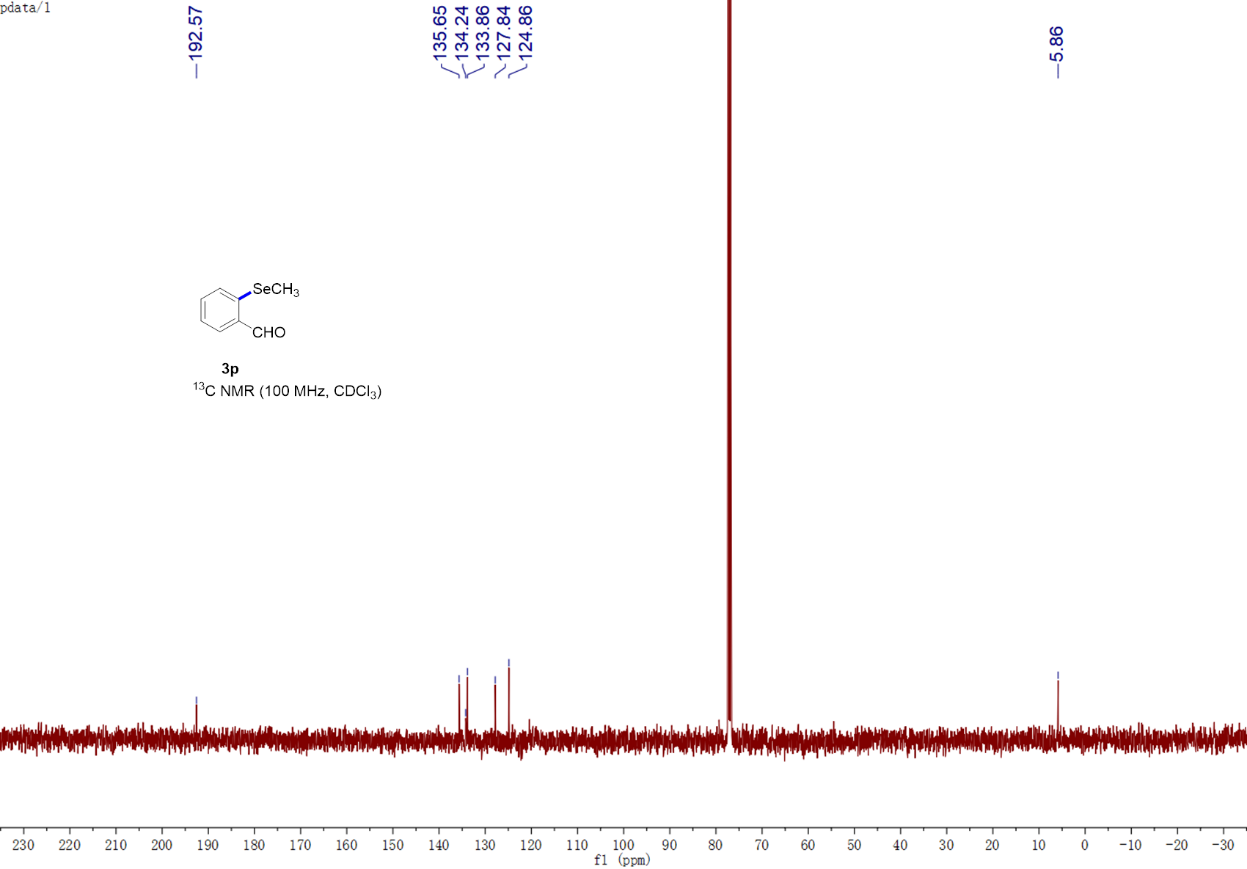
**

**^1^H NMR (400 MHz, Chloroform-*d*) spectrum of 3q**

**
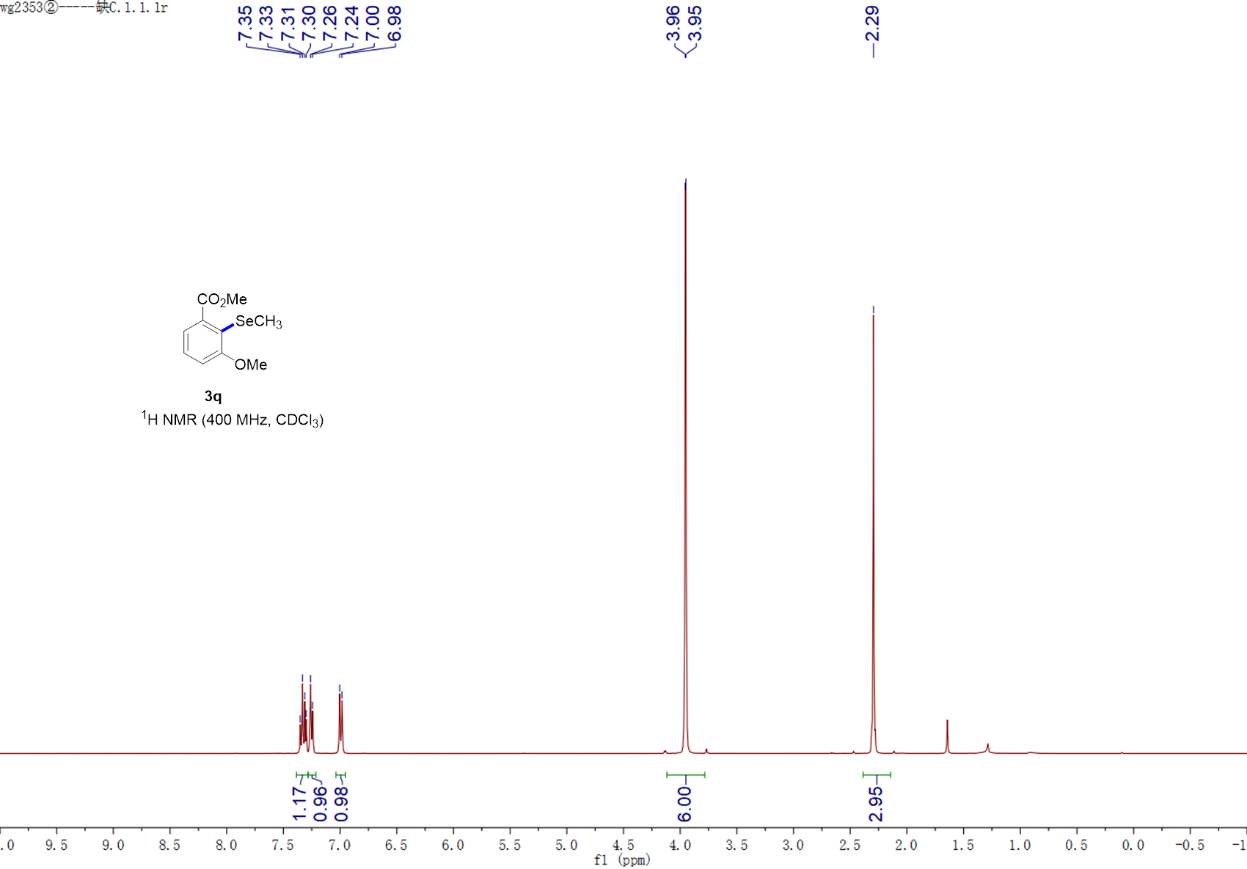
**

**^13^C{^1^H} NMR (100 MHz, Chloroform-*d*) spectrum of 3q**

**
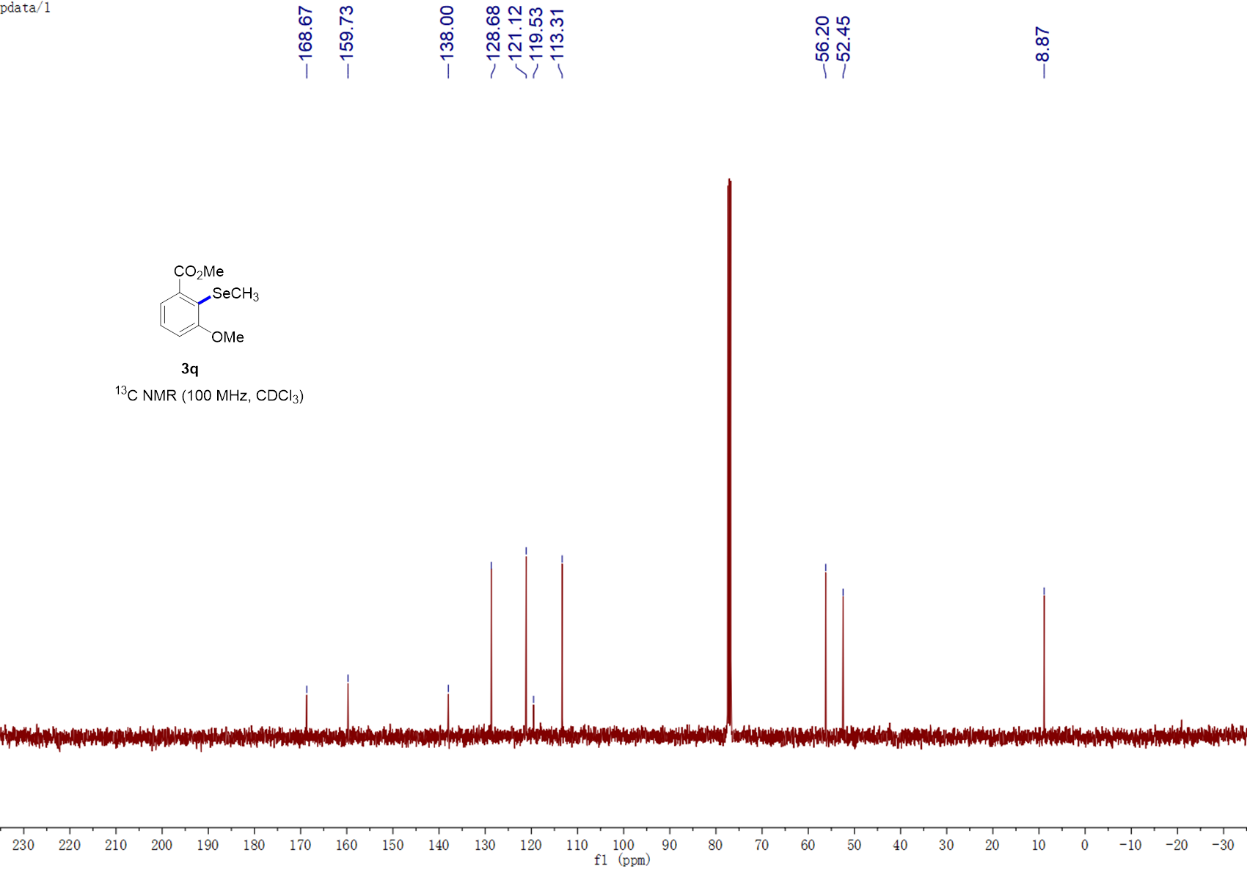
**

**^1^H NMR (400 MHz, Chloroform-*d*) spectrum of 3r**

**
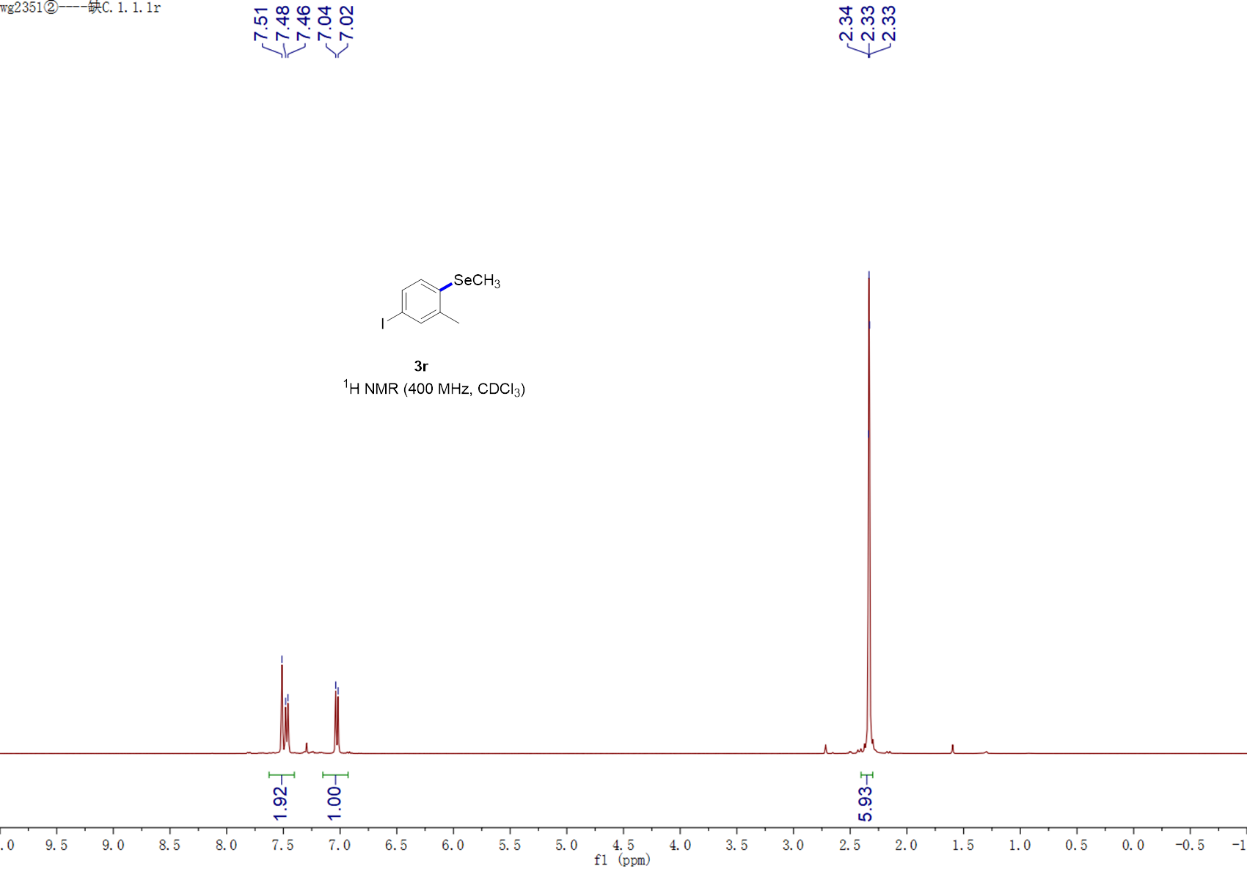
**

**^13^C{^1^H} NMR (100 MHz, Chloroform-*d*) spectrum of 3r**

**
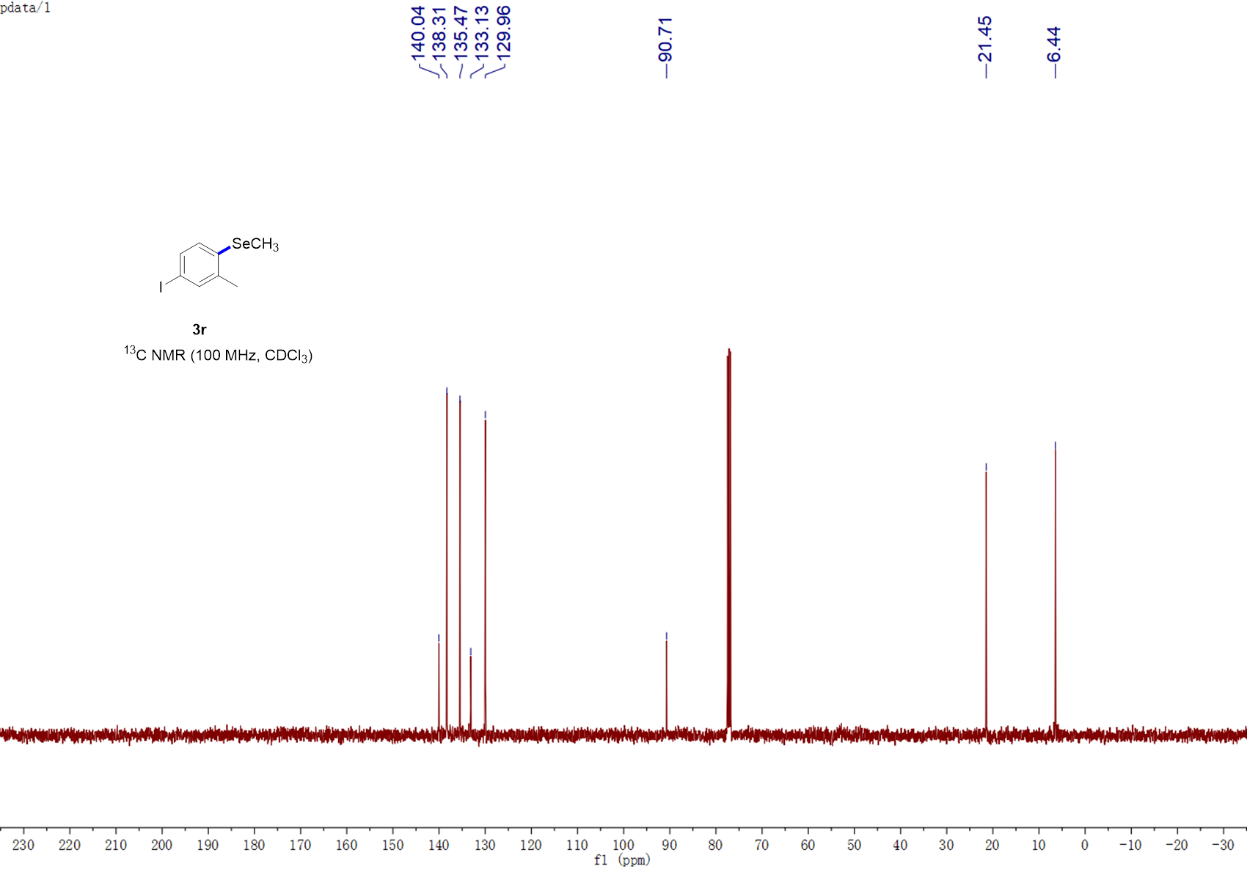
**

**^1^H NMR (400 MHz, Chloroform-*d*) spectrum of 3s**

**
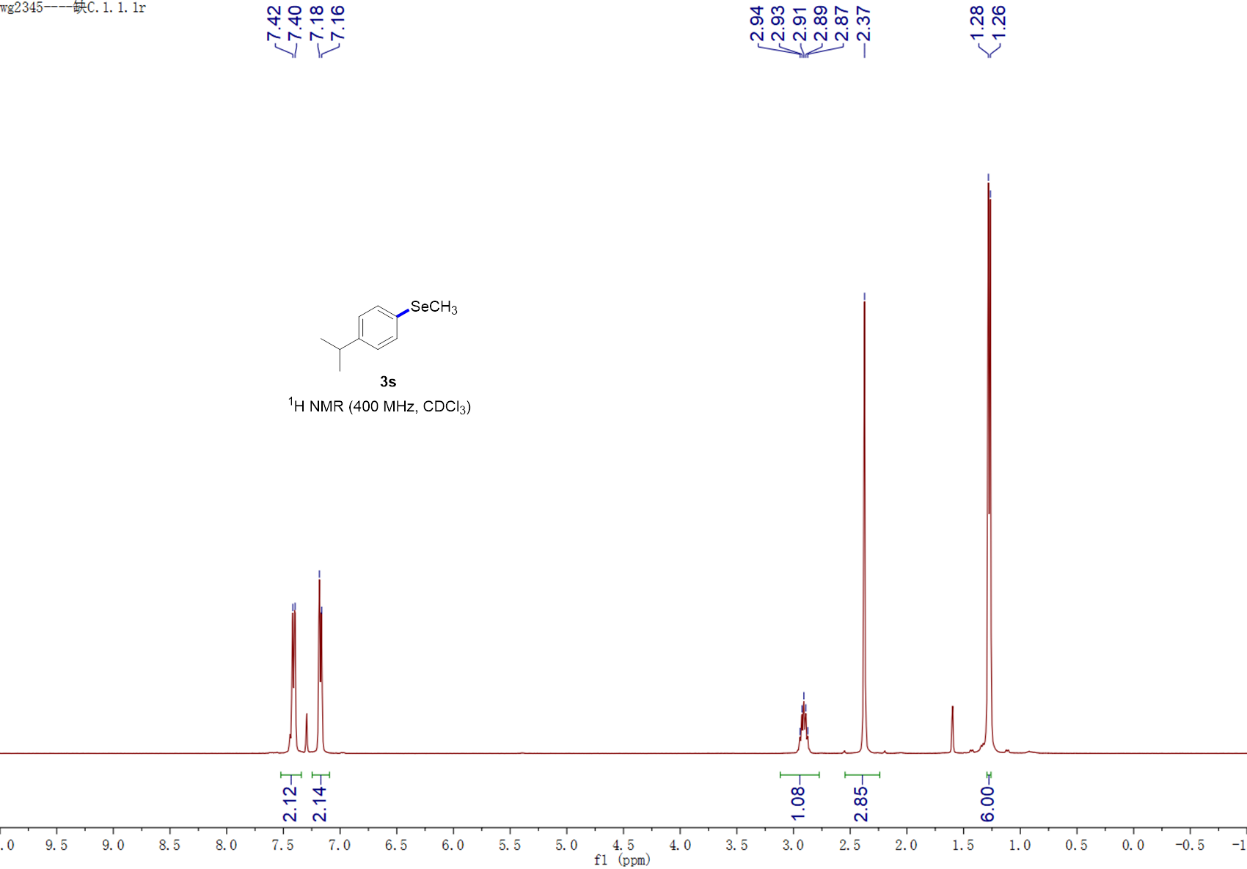
**

**^13^C{^1^H} NMR (100 MHz, Chloroform-*d*) spectrum of 3s**

**
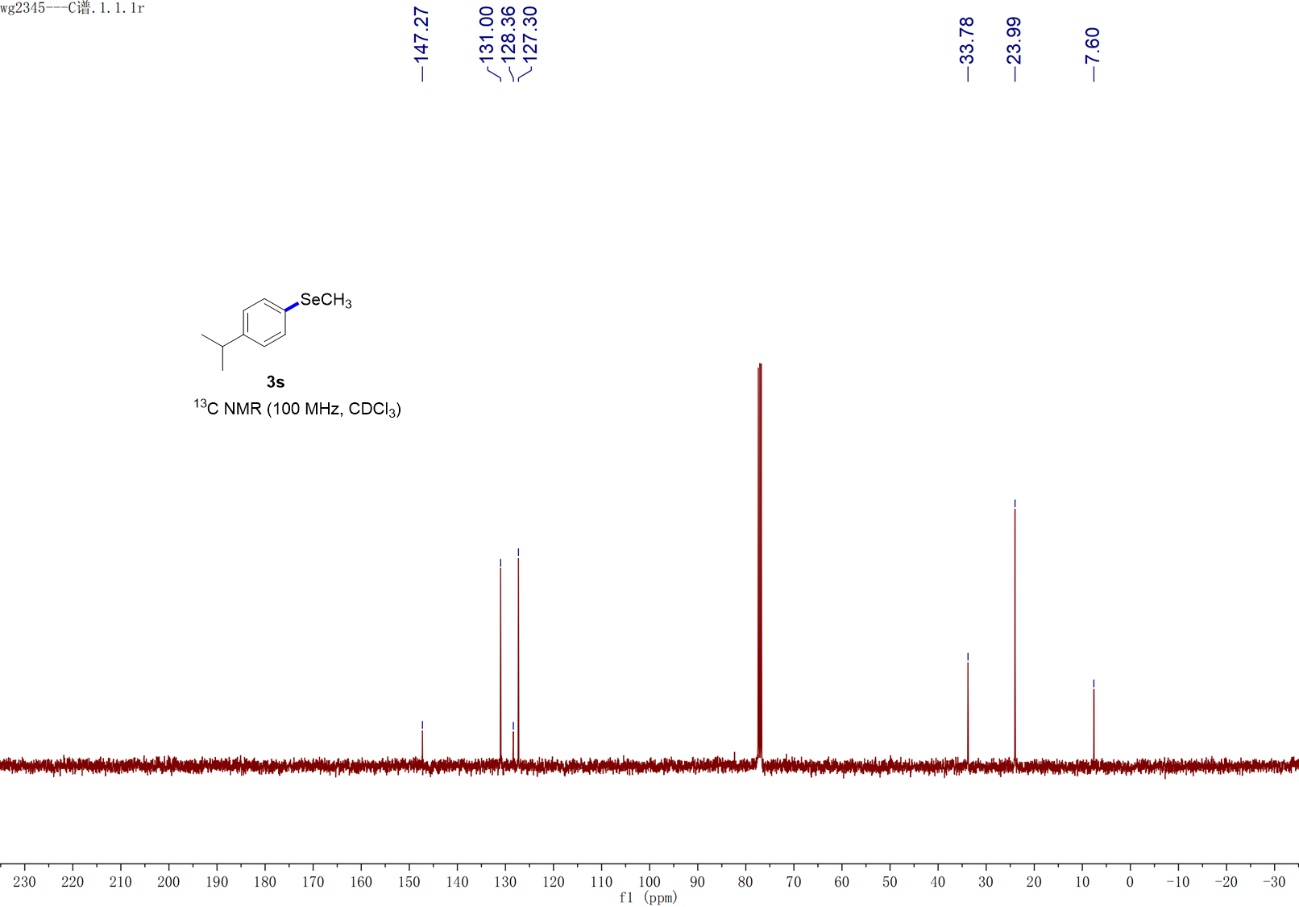
**

**^1^H NMR (400 MHz, Chloroform-*d*) spectrum of 3t**

**
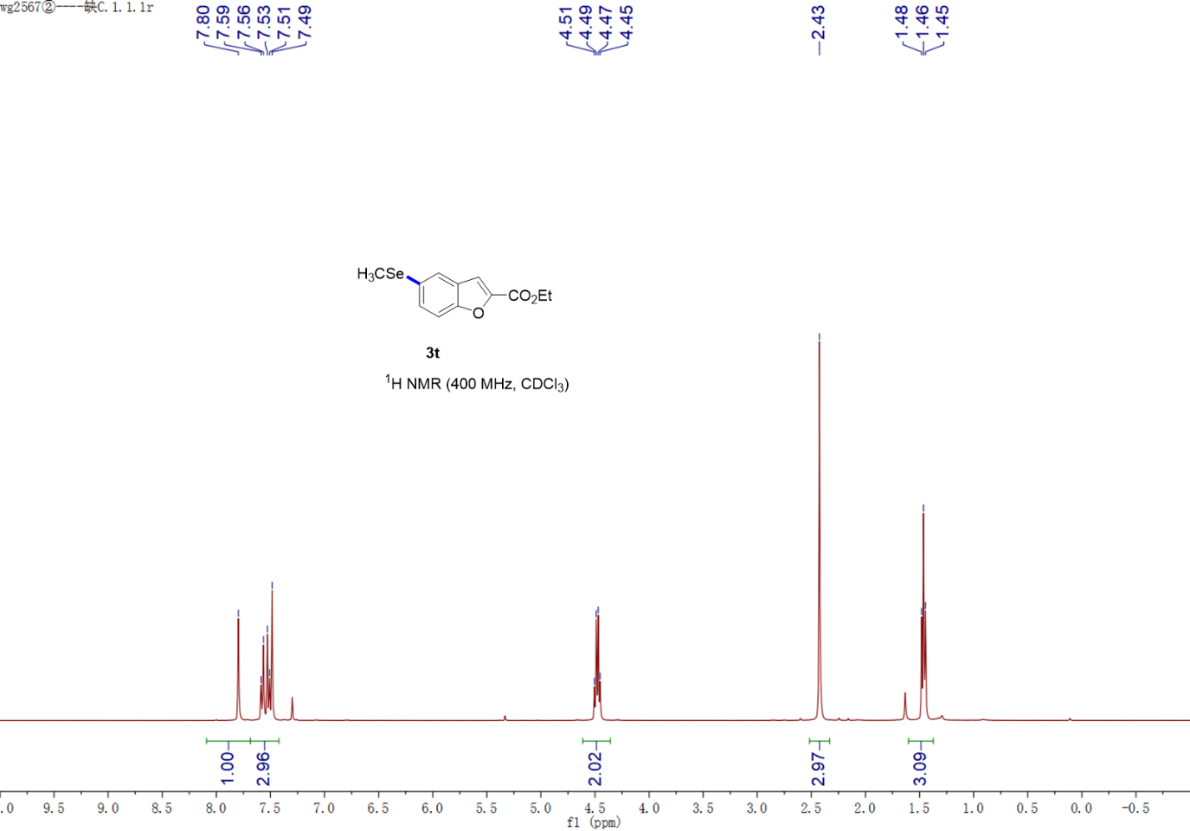
**

**^13^C{^1^H} NMR (100 MHz, Chloroform-*d*) spectrum of 3t**

**
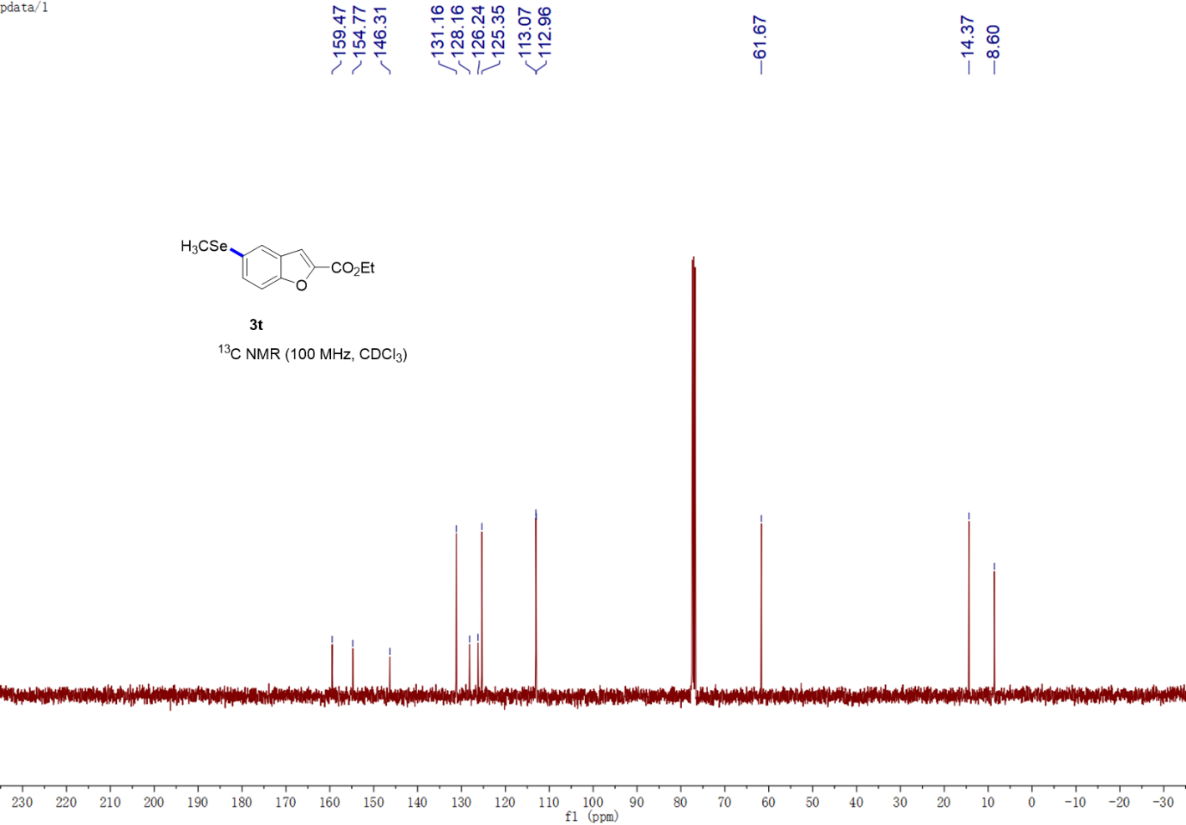
**

**^1^H NMR (400 MHz, Chloroform-*d*) spectrum of 3u**

**
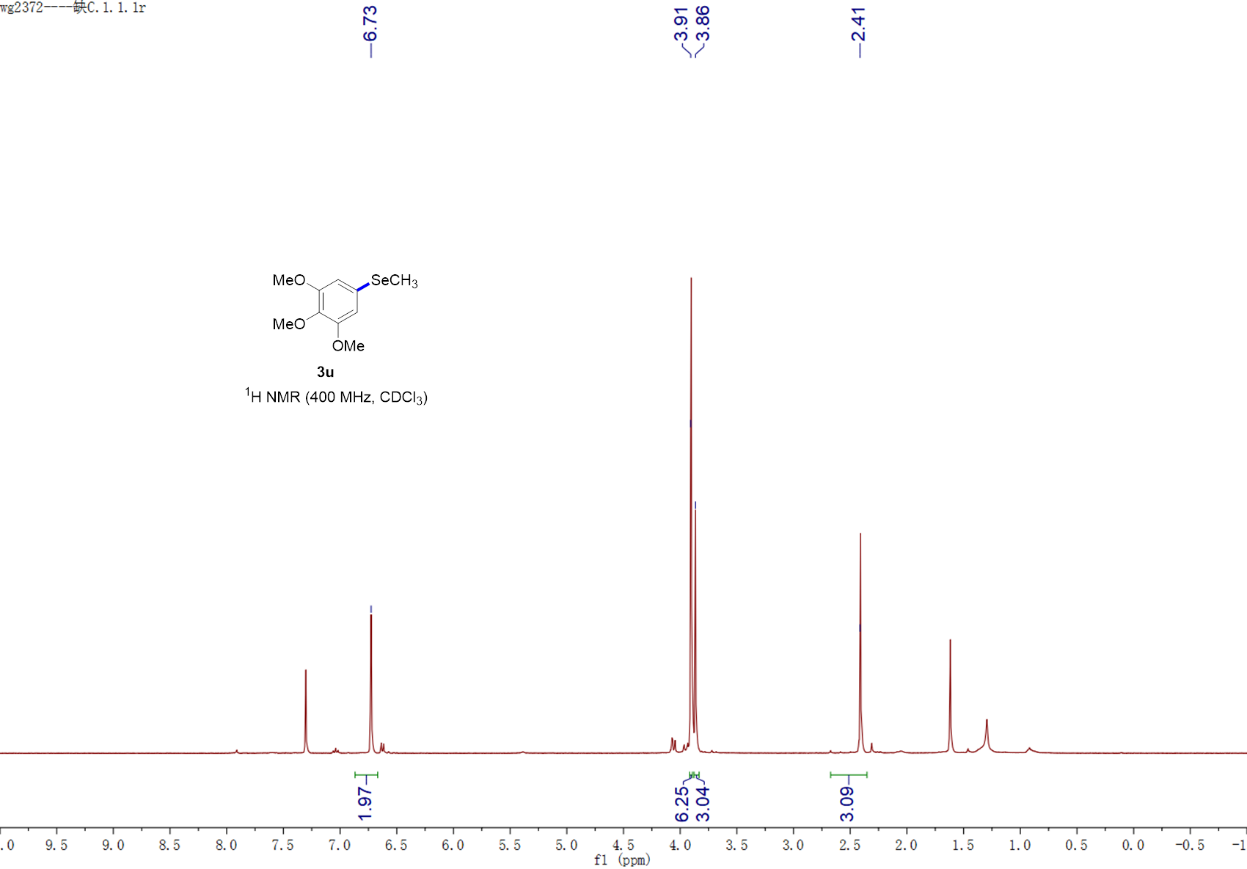
**

**^13^C{^1^H} NMR (100 MHz, Chloroform-*d*) spectrum of 3u**

**
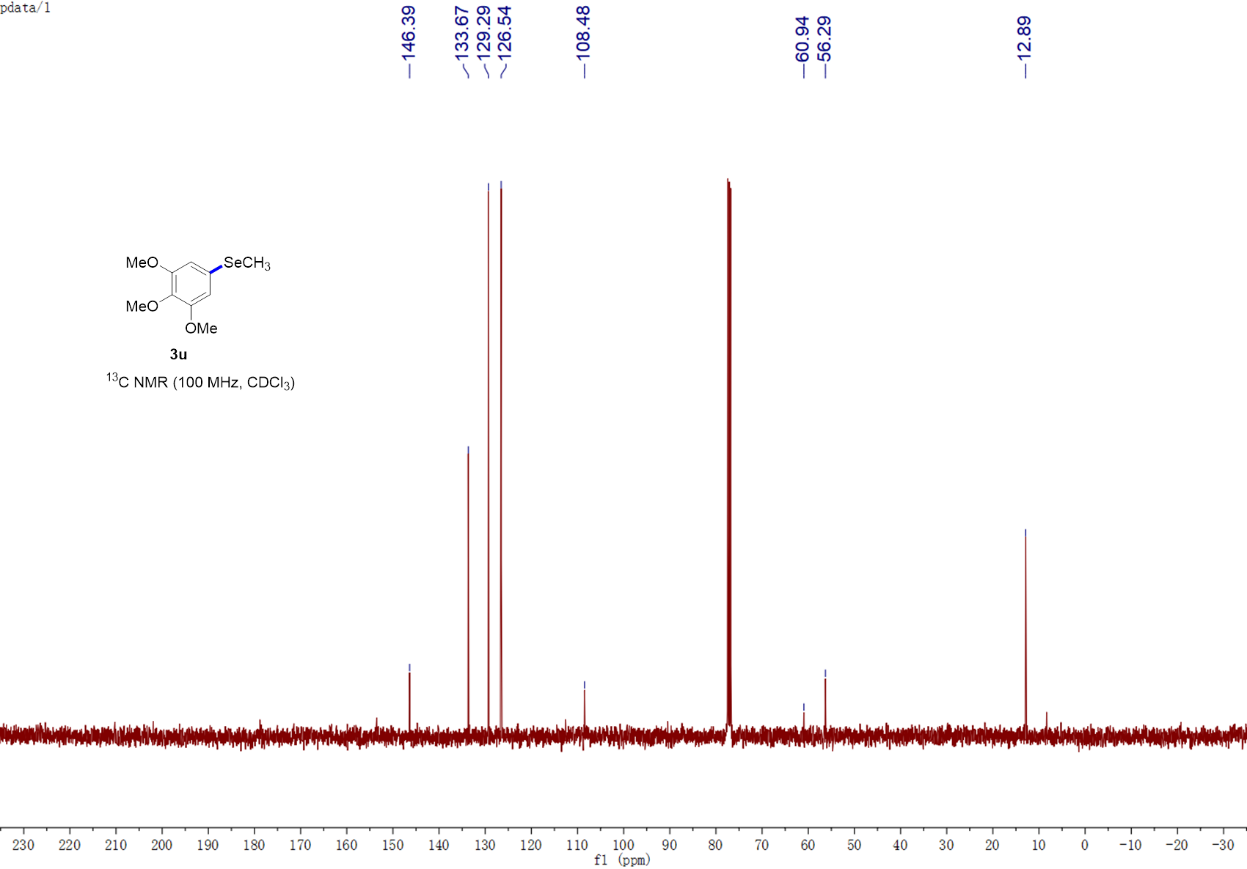
**

**^1^H NMR (400 MHz, Chloroform-*d*) spectrum of 3v**

**
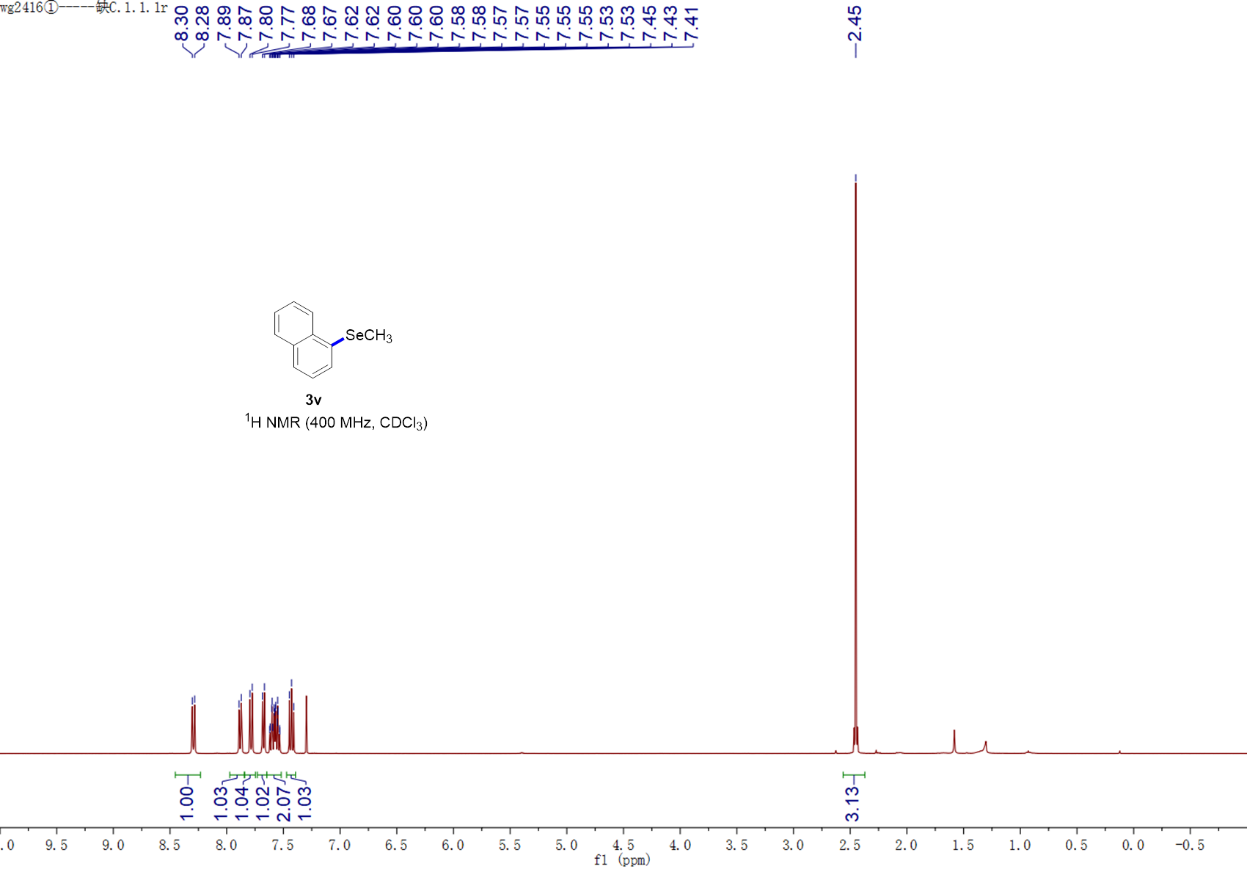
**

**^13^C{^1^H} NMR (100 MHz, Chloroform-*d*) spectrum of 3v**

**
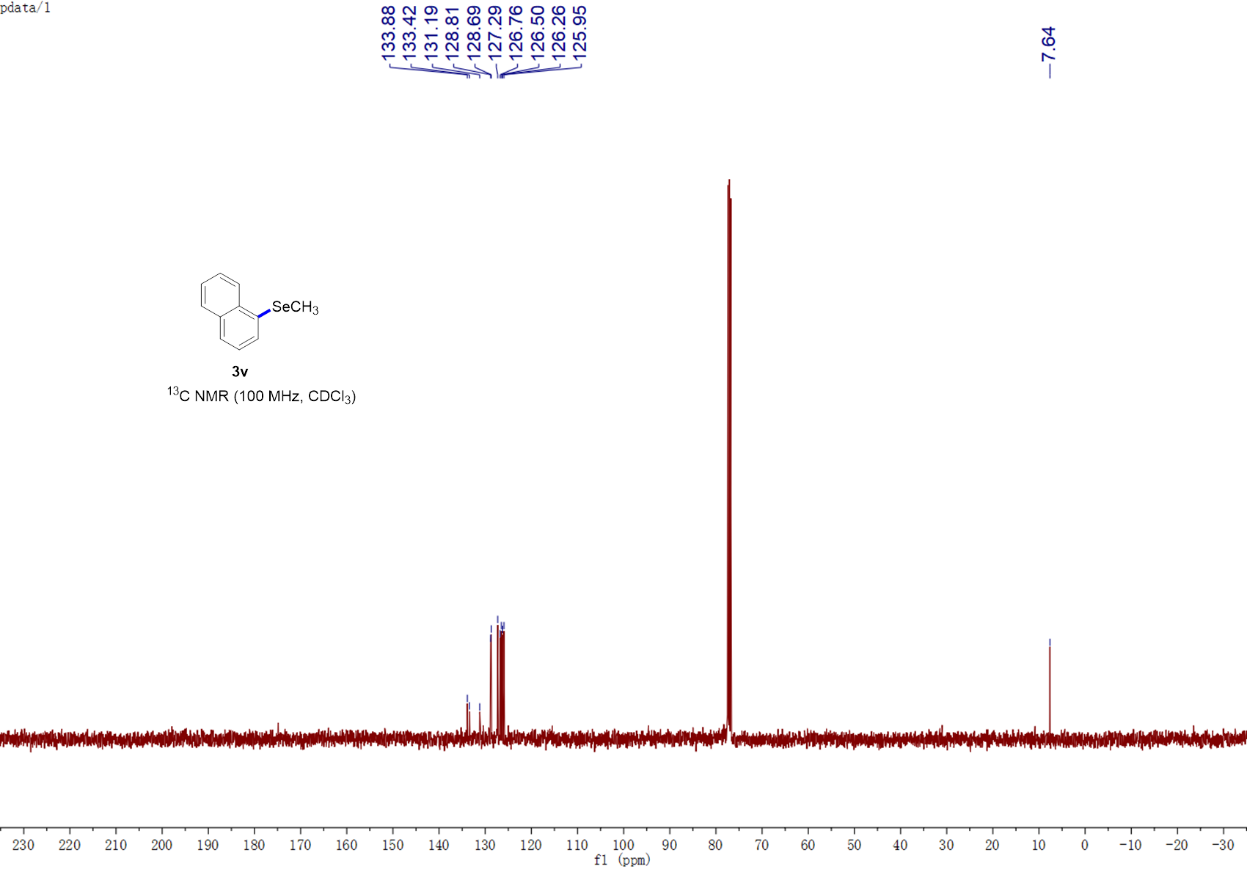
**

**^1^H NMR (400 MHz, Chloroform-*d*) spectrum of 3w**

**
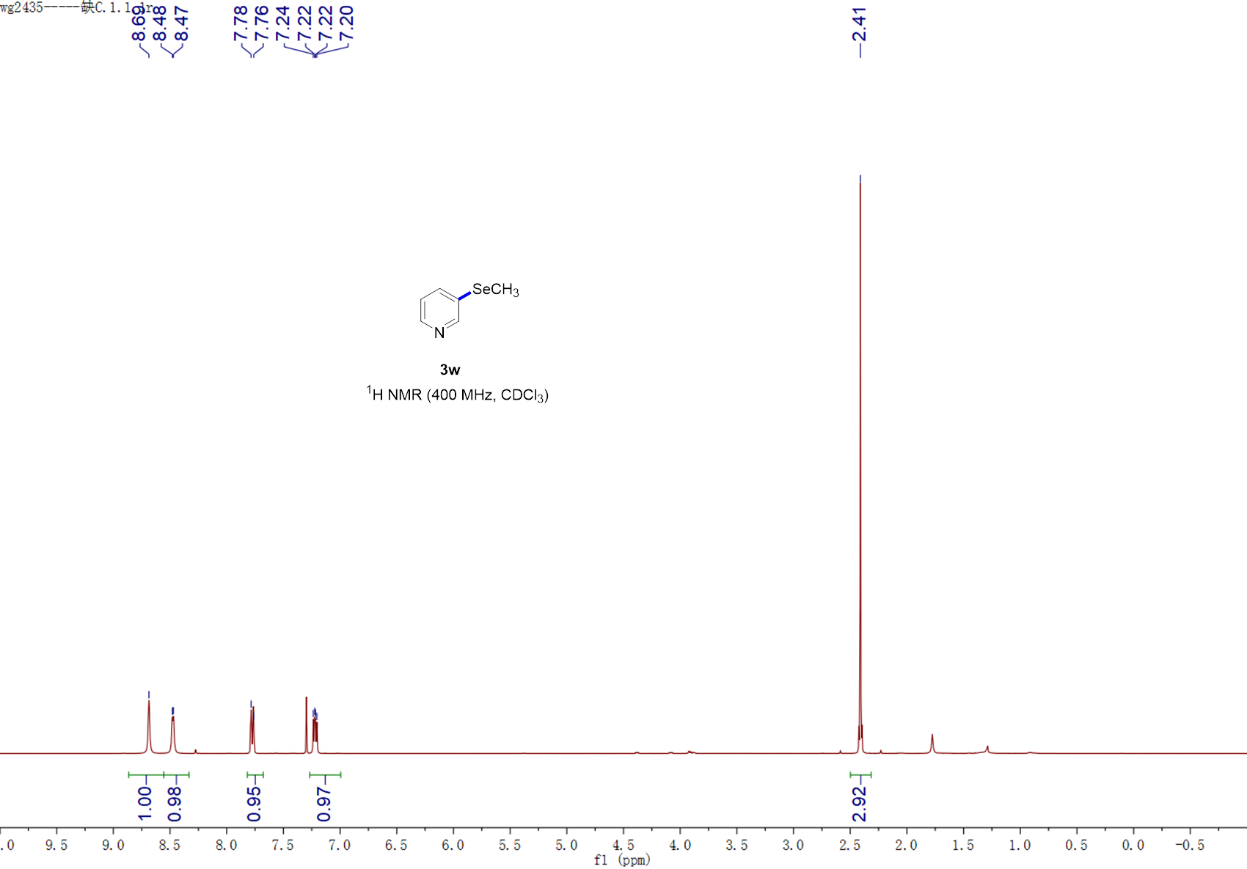
**

**^13^C{^1^H} NMR (100 MHz, Chloroform-*d*) spectrum of 3w**

**
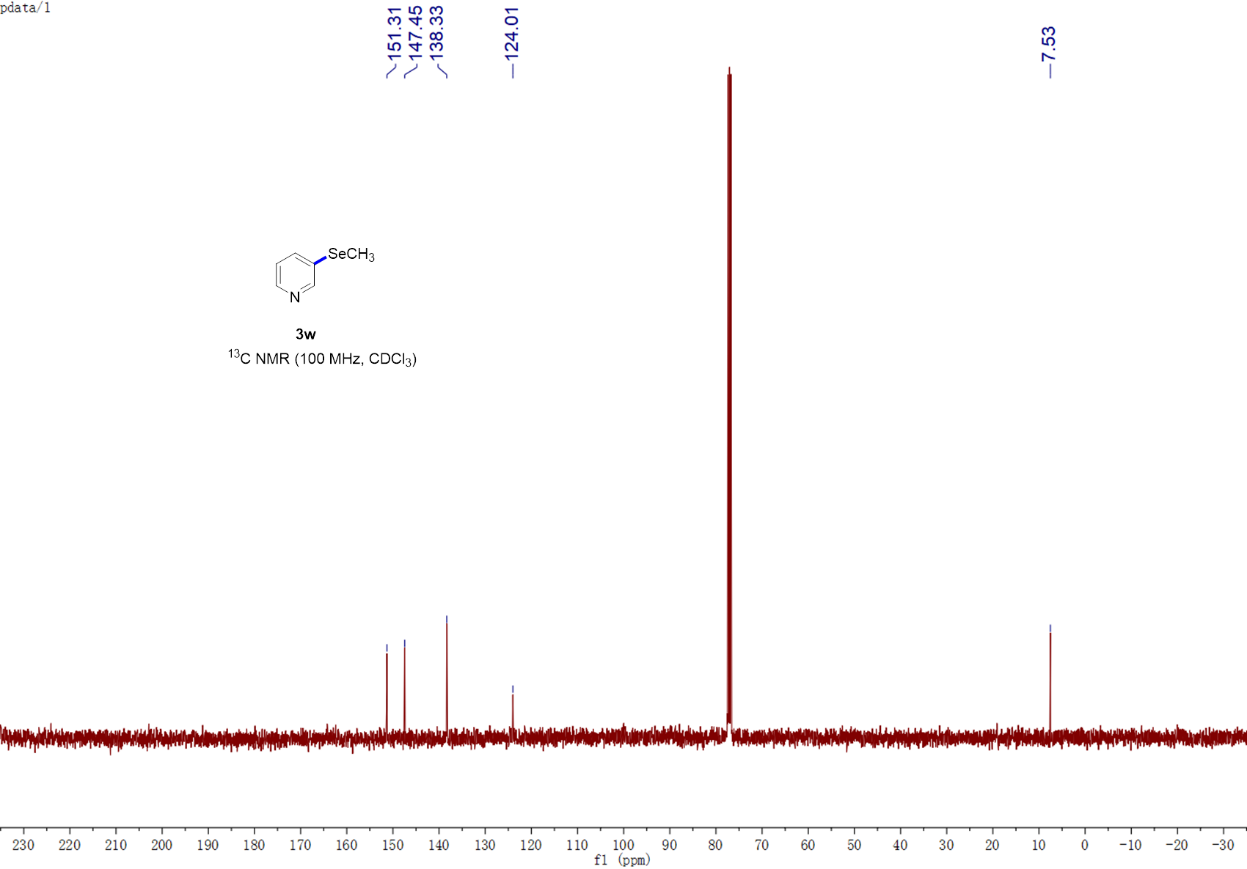
**

**^1^H NMR (400 MHz, Chloroform-*d*) spectrum of 3x**

**
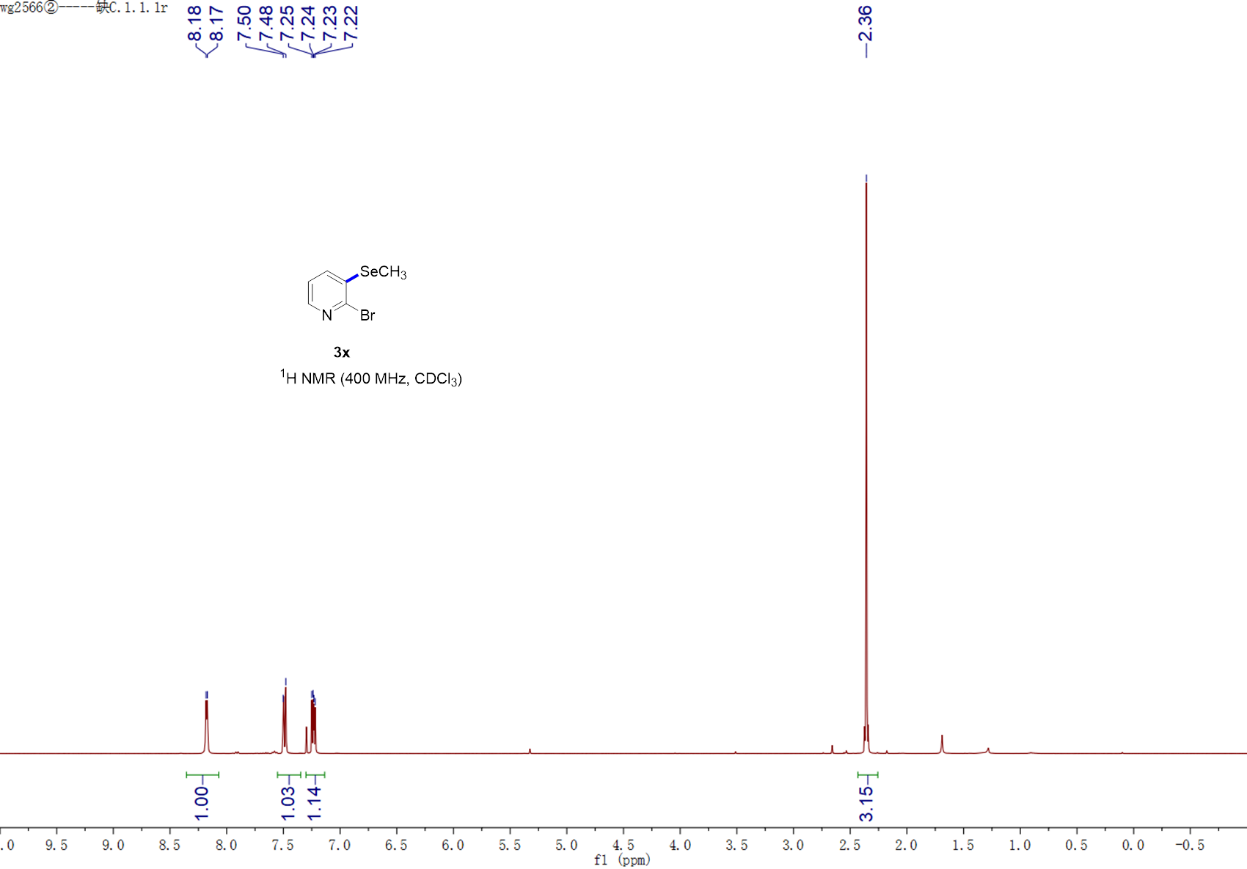
**

**^13^C{^1^H} NMR (100 MHz, Chloroform-*d*) spectrum of 3x**

**
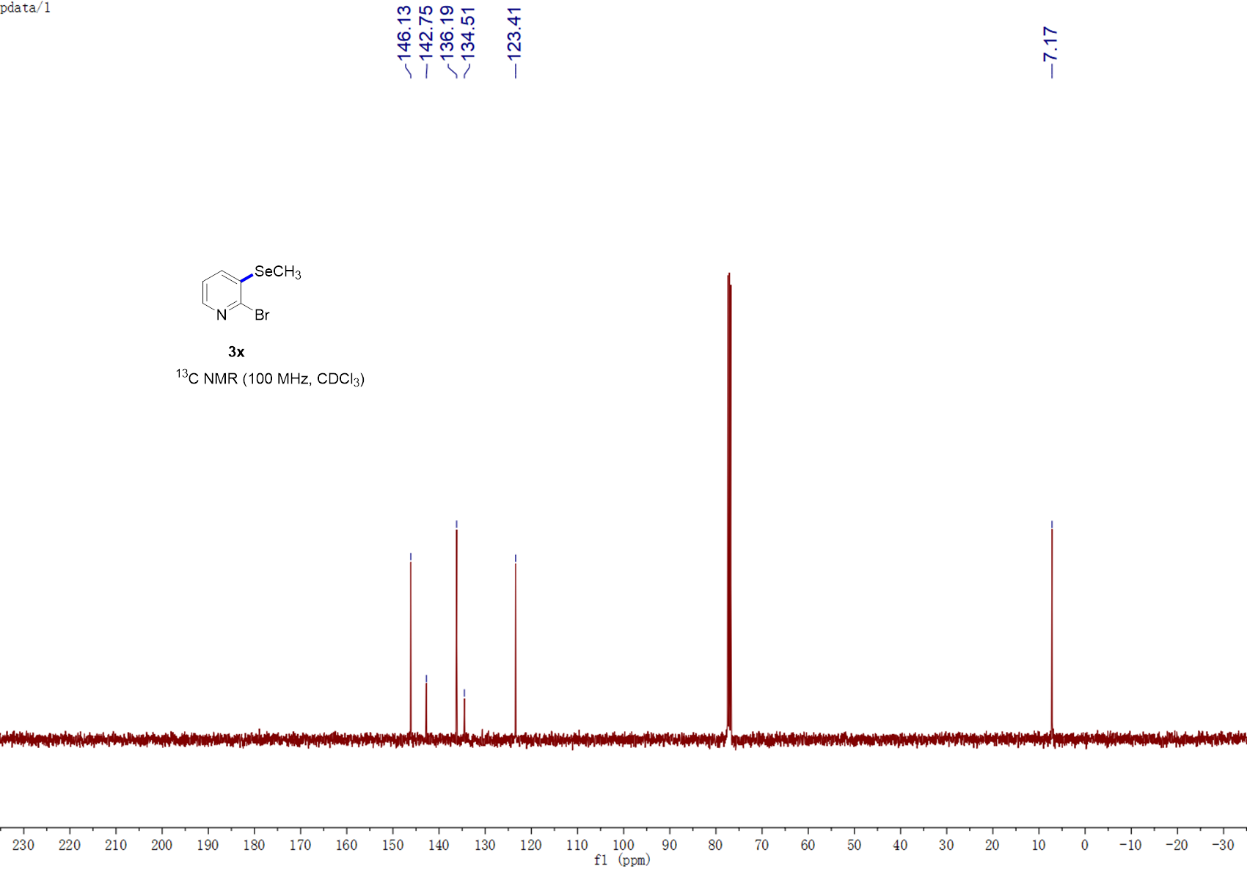
**

**^1^H NMR (400 MHz, Chloroform-*d*) spectrum of 3y**

**
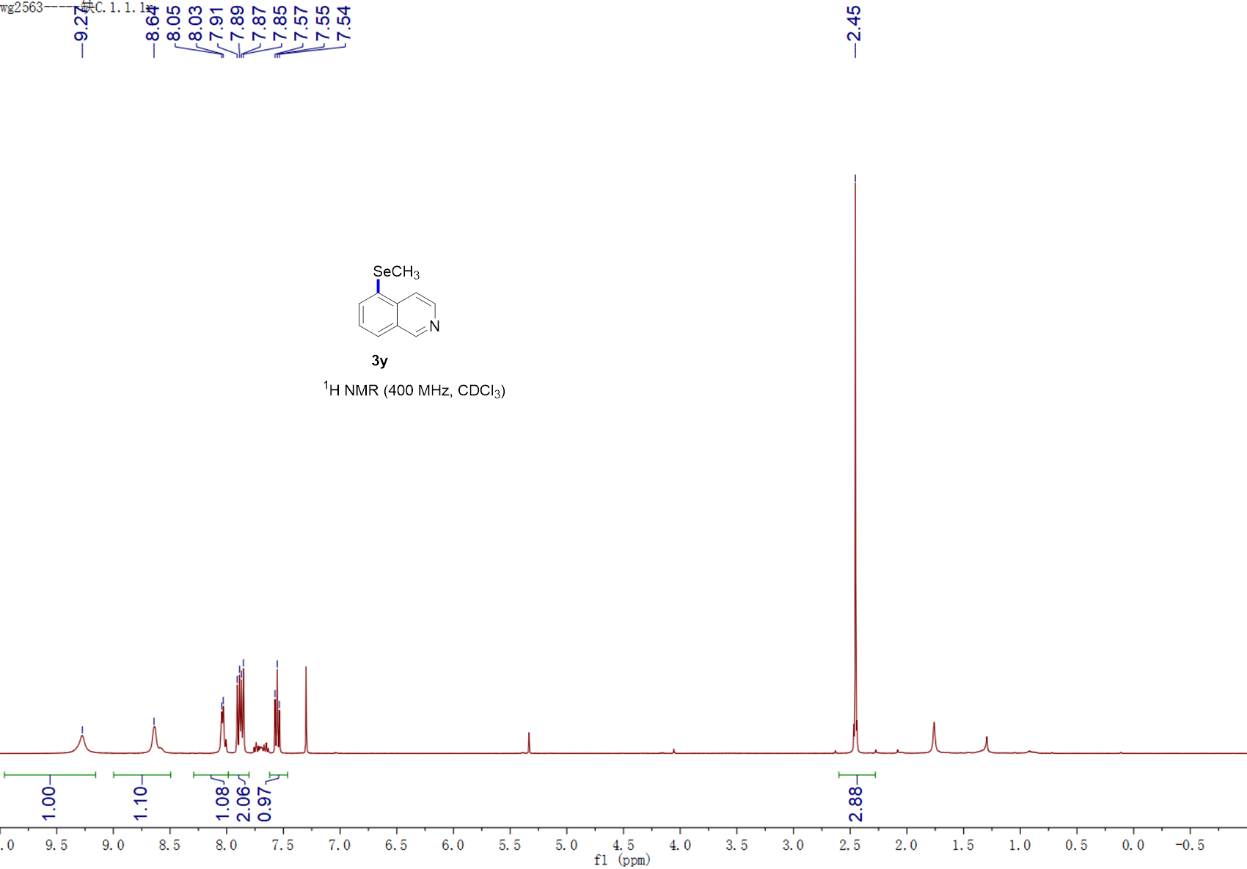
**

**^13^C{^1^H} NMR (100 MHz, Chloroform-*d*) spectrum of 3y**

**
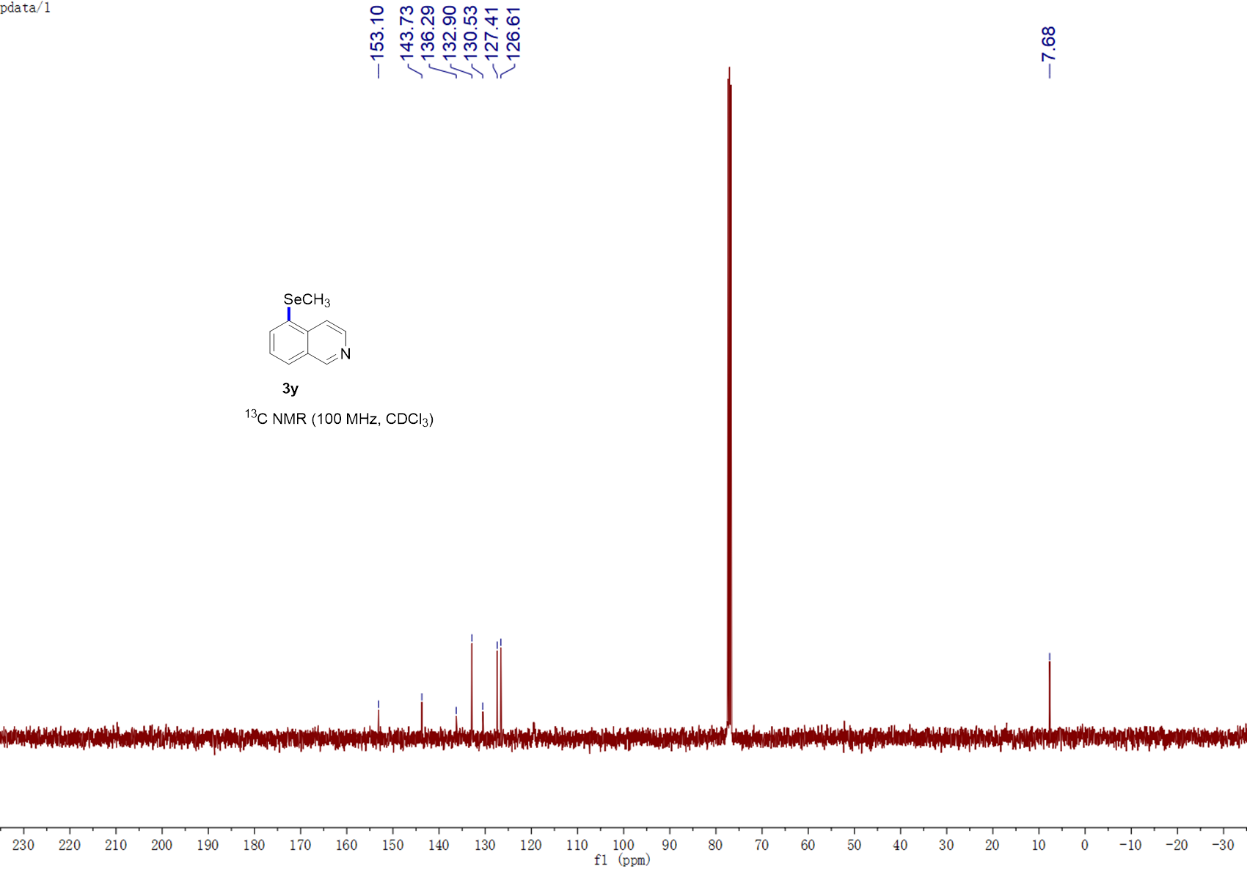
**

**^1^H NMR (400 MHz, Chloroform-*d*) spectrum of 3z**

**
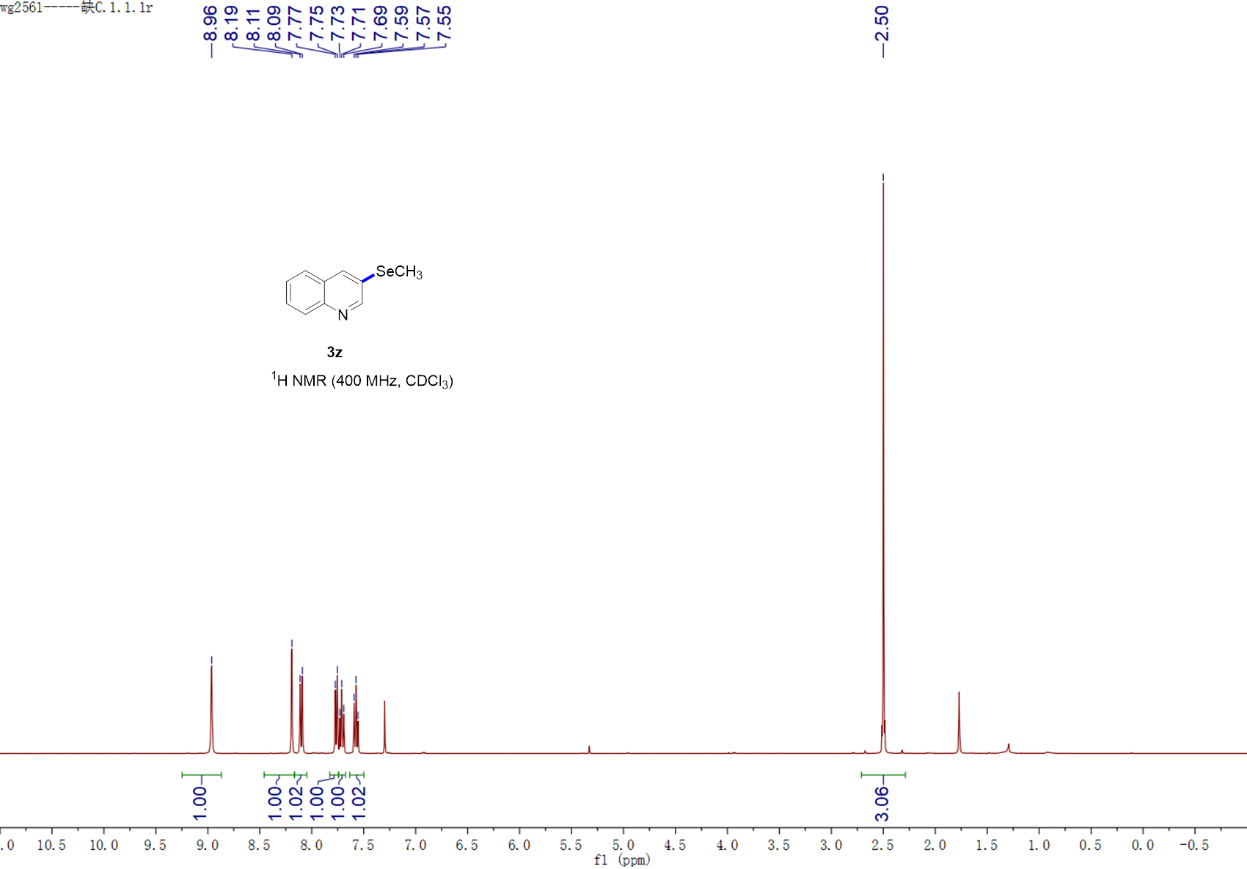
**

**^13^C{^1^H} NMR (100 MHz, Chloroform-*d*) spectrum of 3z**

**
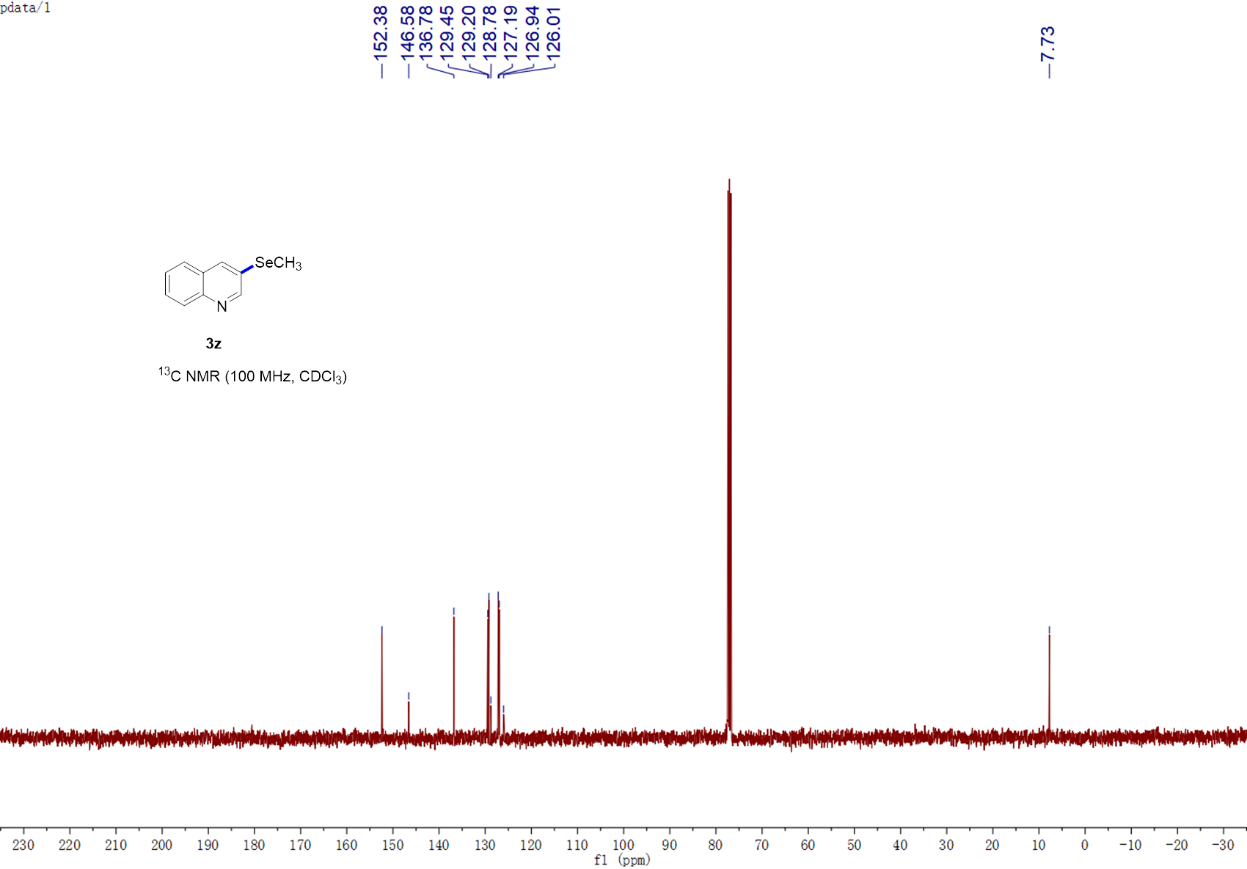
**

**^1^H NMR (400 MHz, Chloroform-*d*) spectrum of 3aa**

**
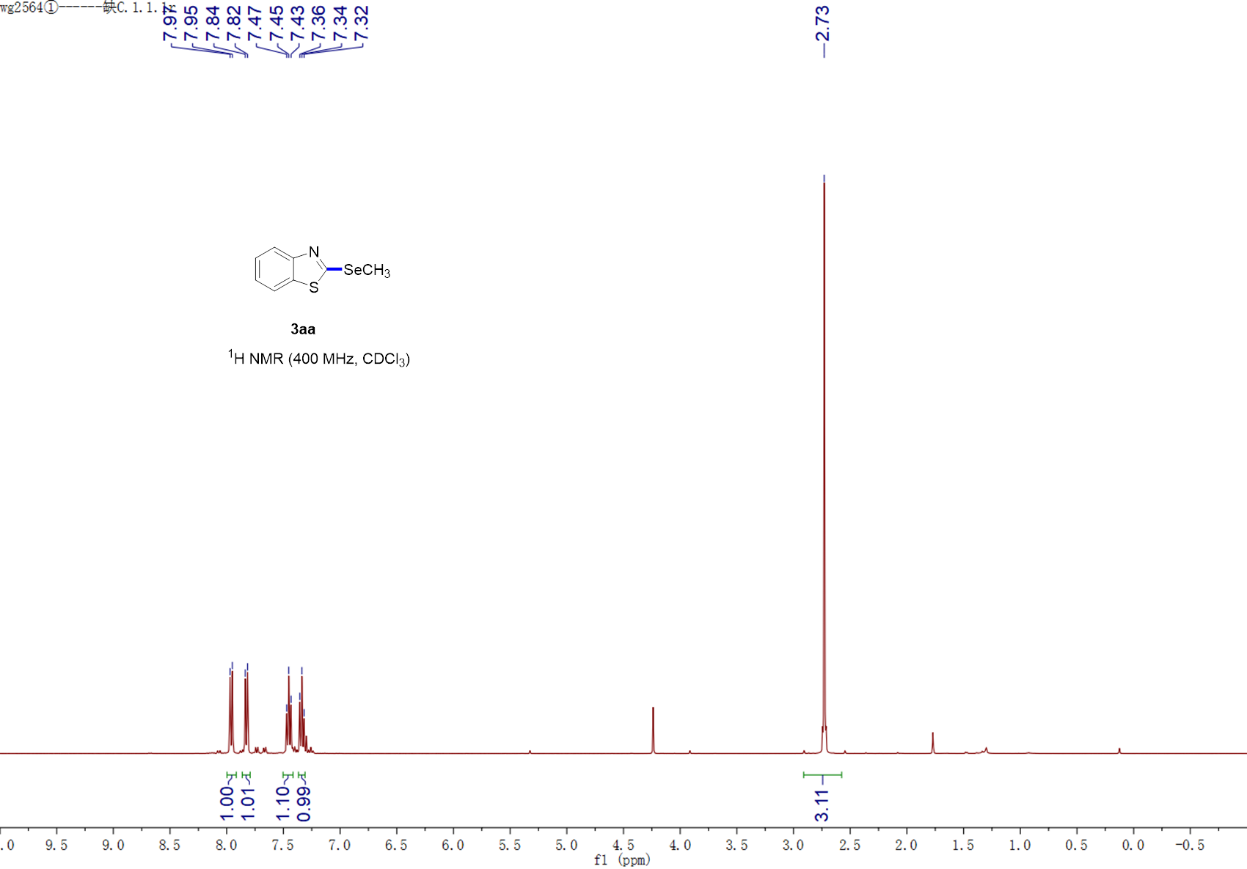
**

**^13^C{^1^H} NMR (100 MHz, Chloroform-*d*) spectrum of 3aa**

**
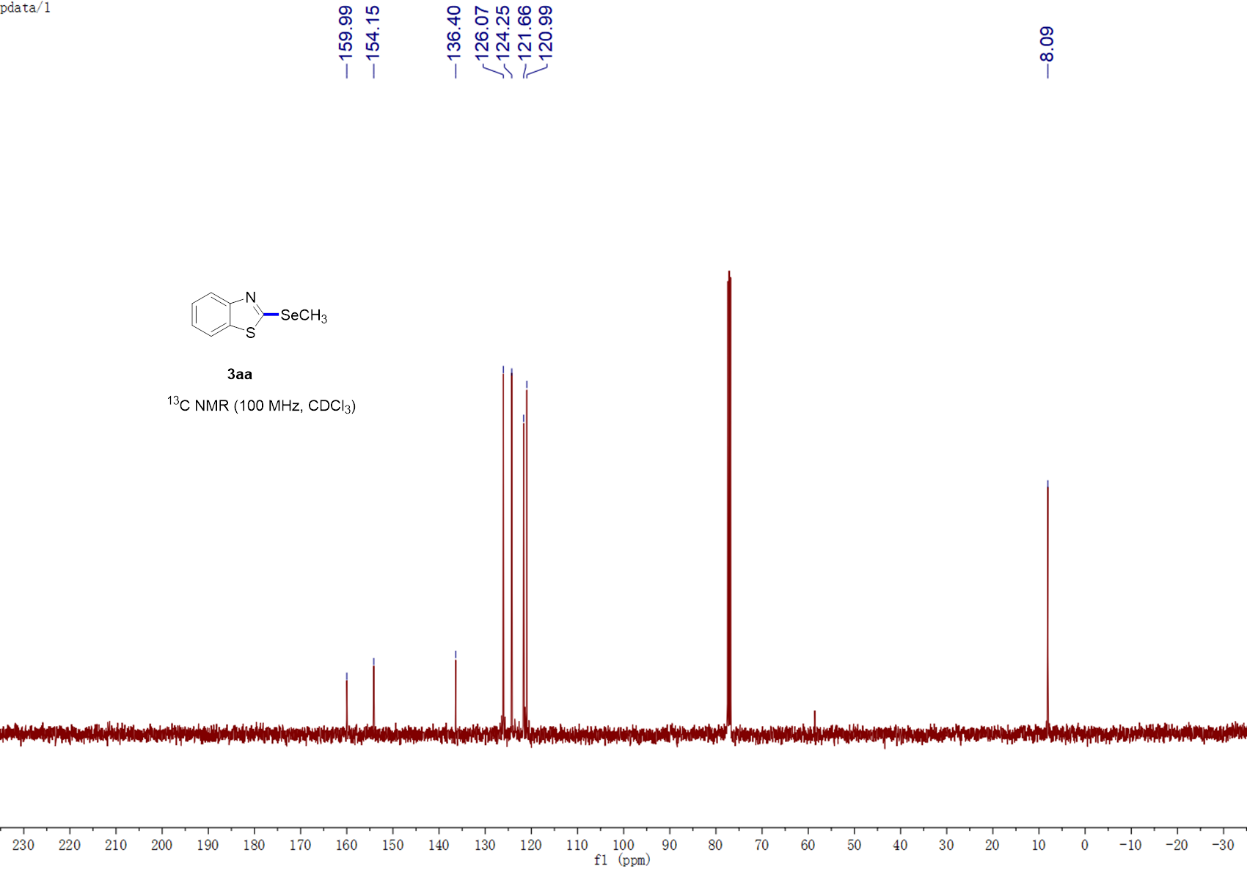
**

**^1^H NMR (400 MHz, Chloroform-*d*) spectrum of 4a**

**
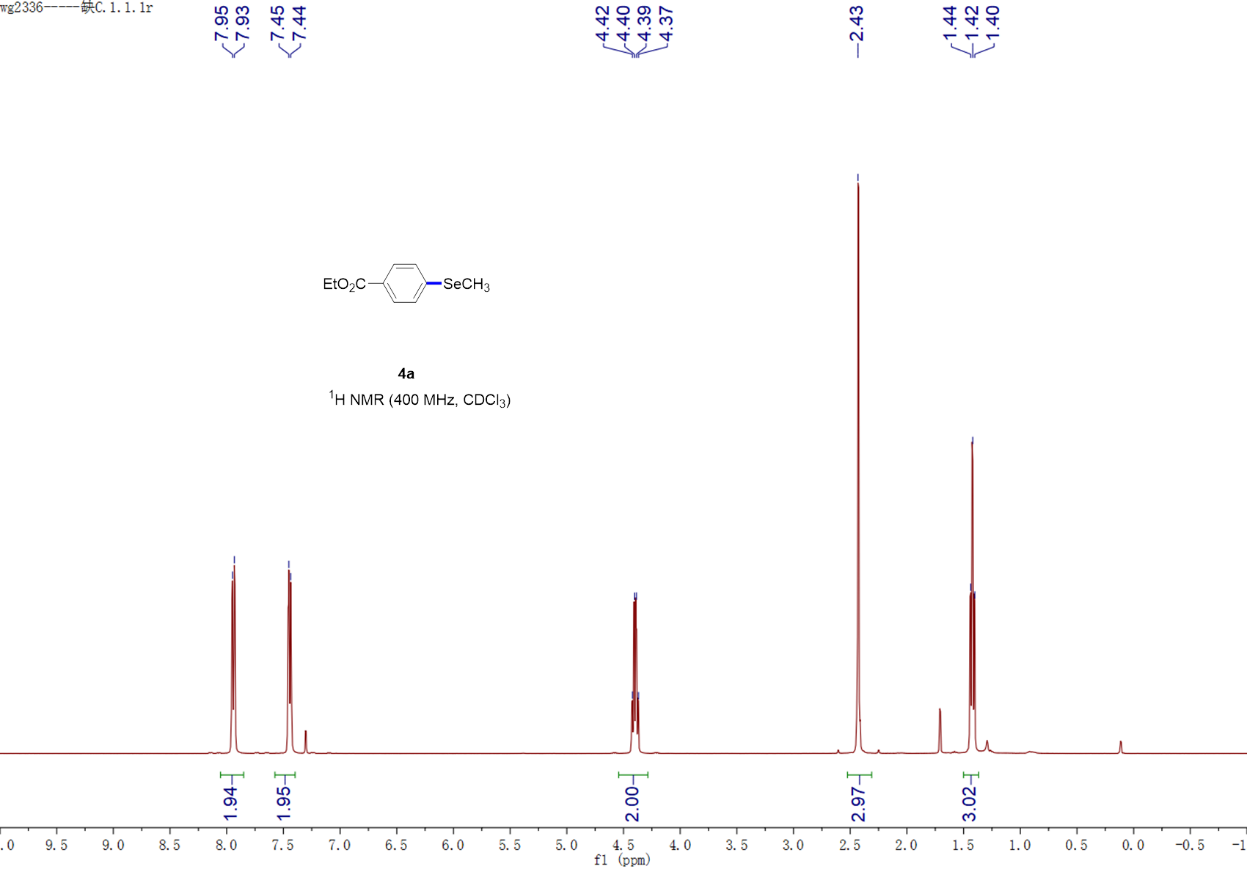
**

**^13^C{^1^H} NMR (100 MHz, Chloroform-*d*) spectrum of 4a**

**
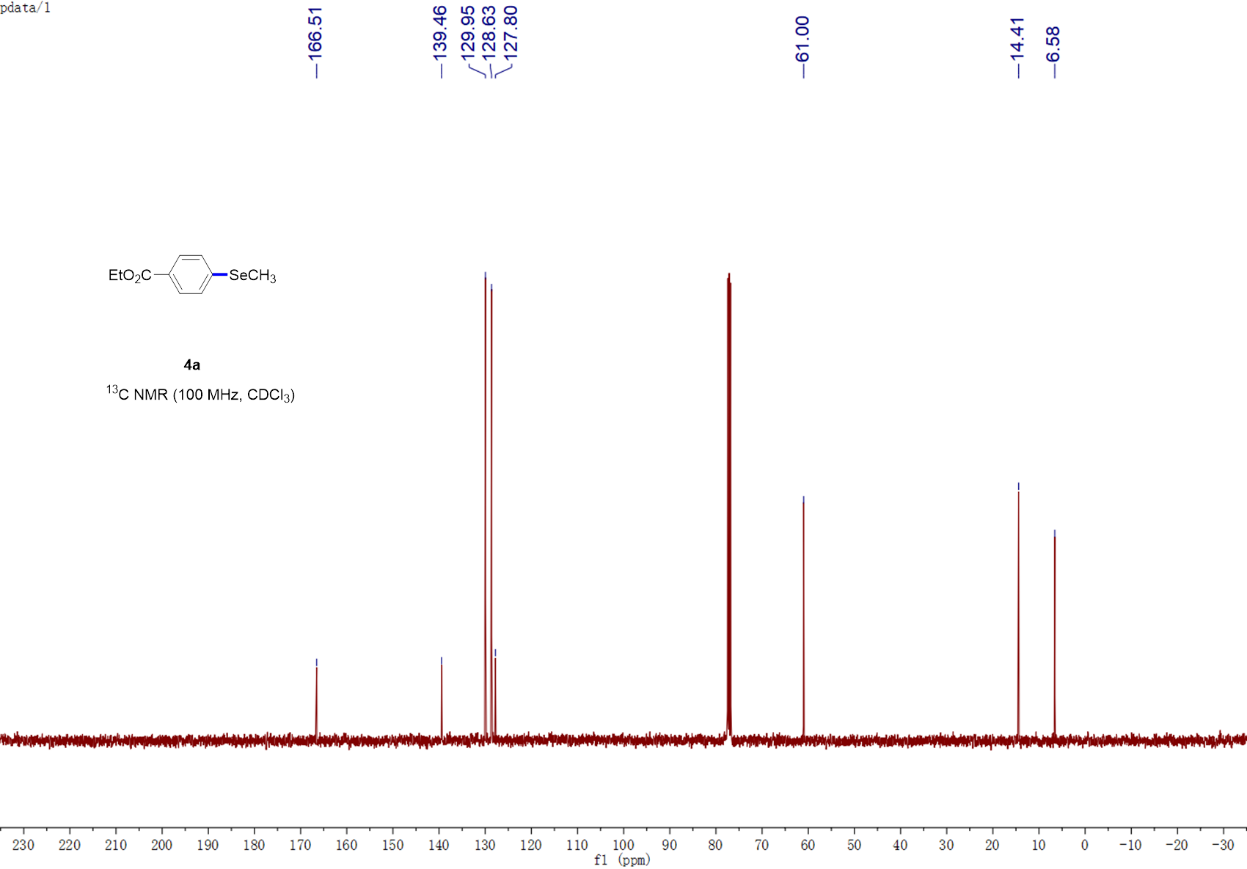
**

**^1^H NMR (400 MHz, Chloroform-*d*) spectrum of 4b**

**
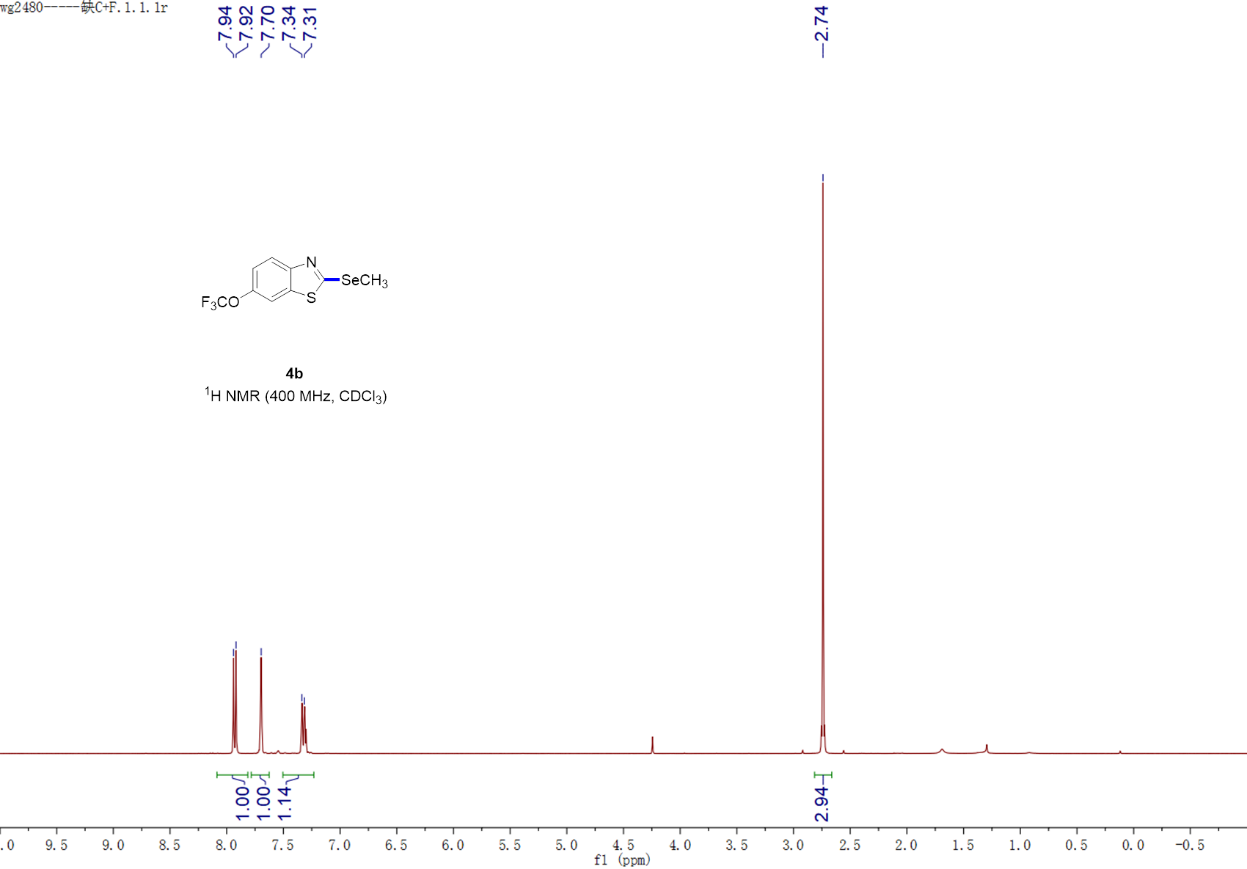
**

**^13^C{^1^H} NMR (100 MHz, Chloroform-*d*) spectrum of 4b**

**
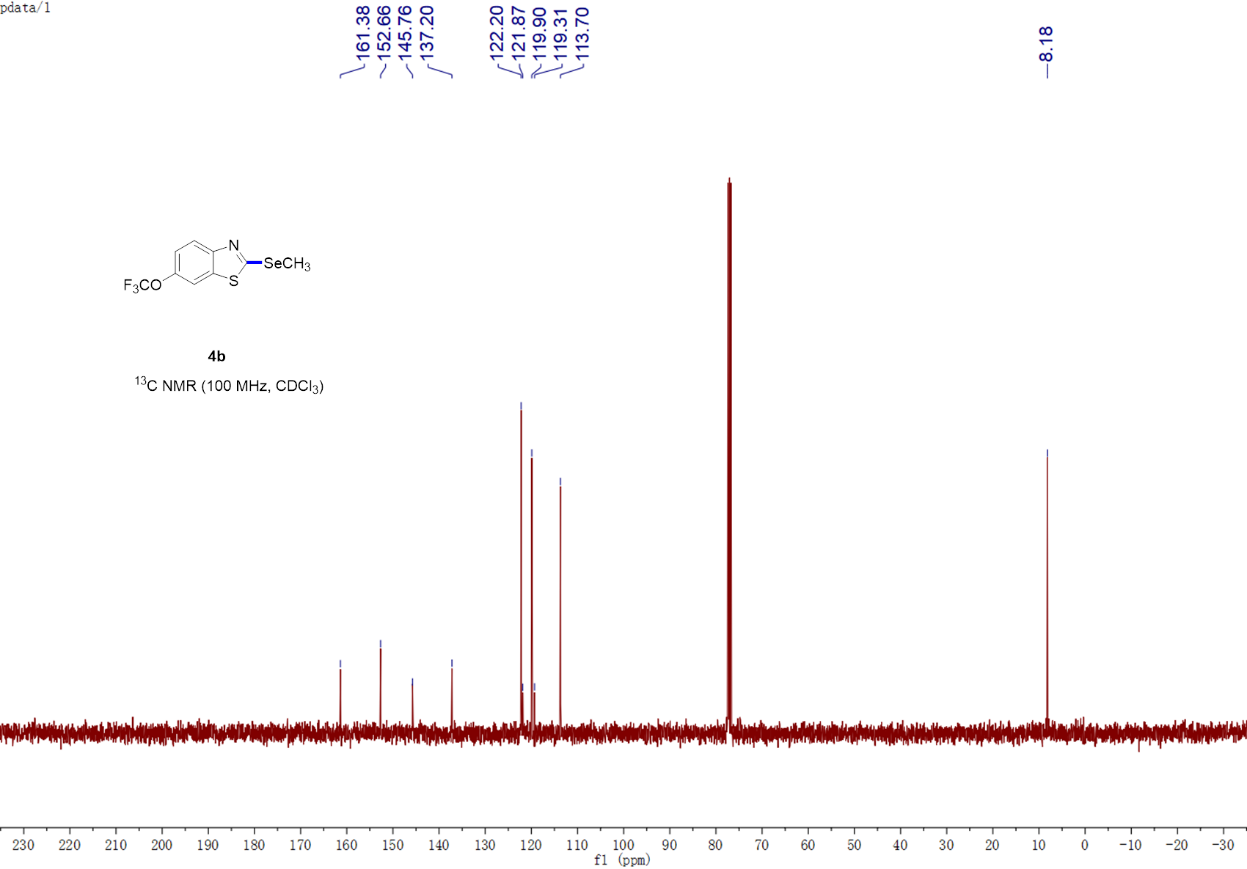
**

**^19^F NMR (375 MHz, Chloroform-*d*) spectrum of 4b**

**
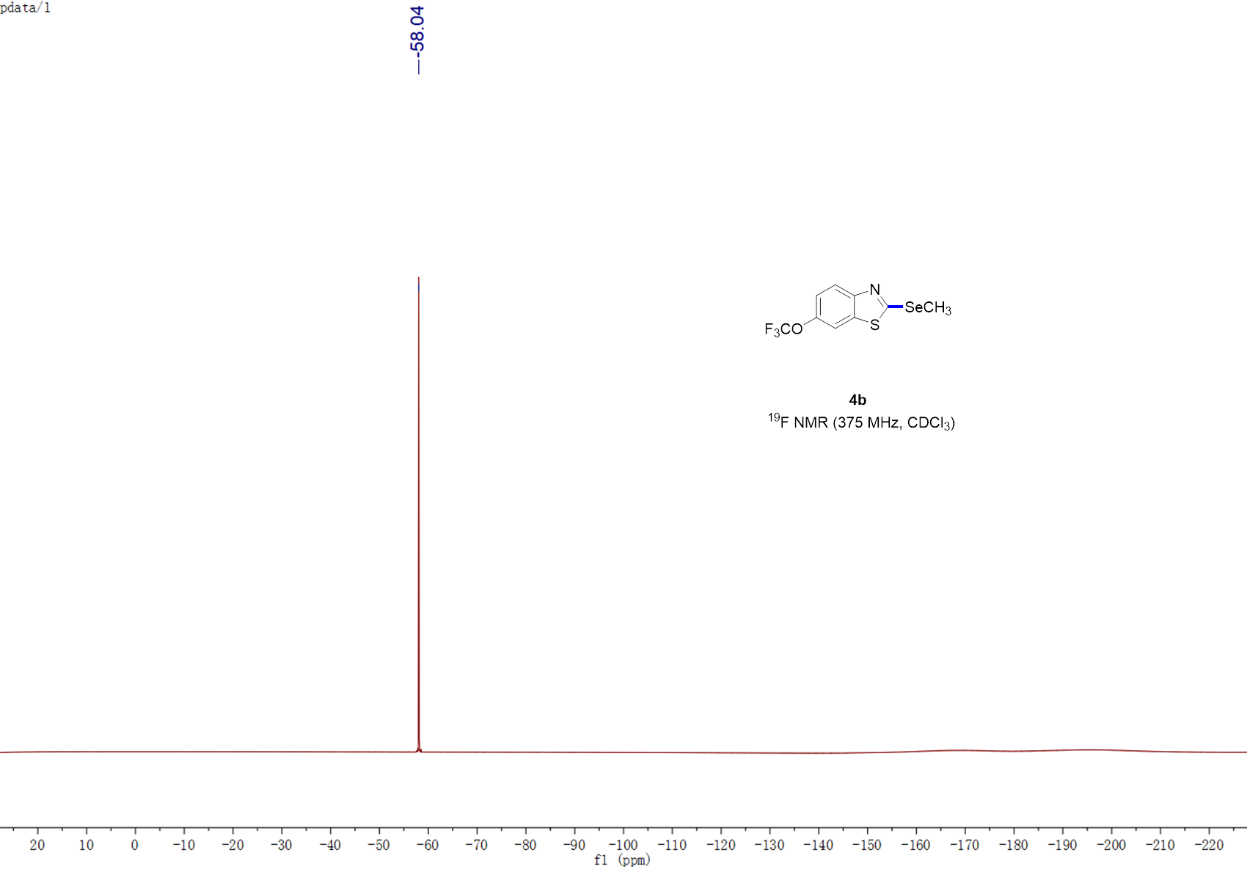
**

**^1^H NMR (400 MHz, Chloroform-*d*) spectrum of 4c**

**
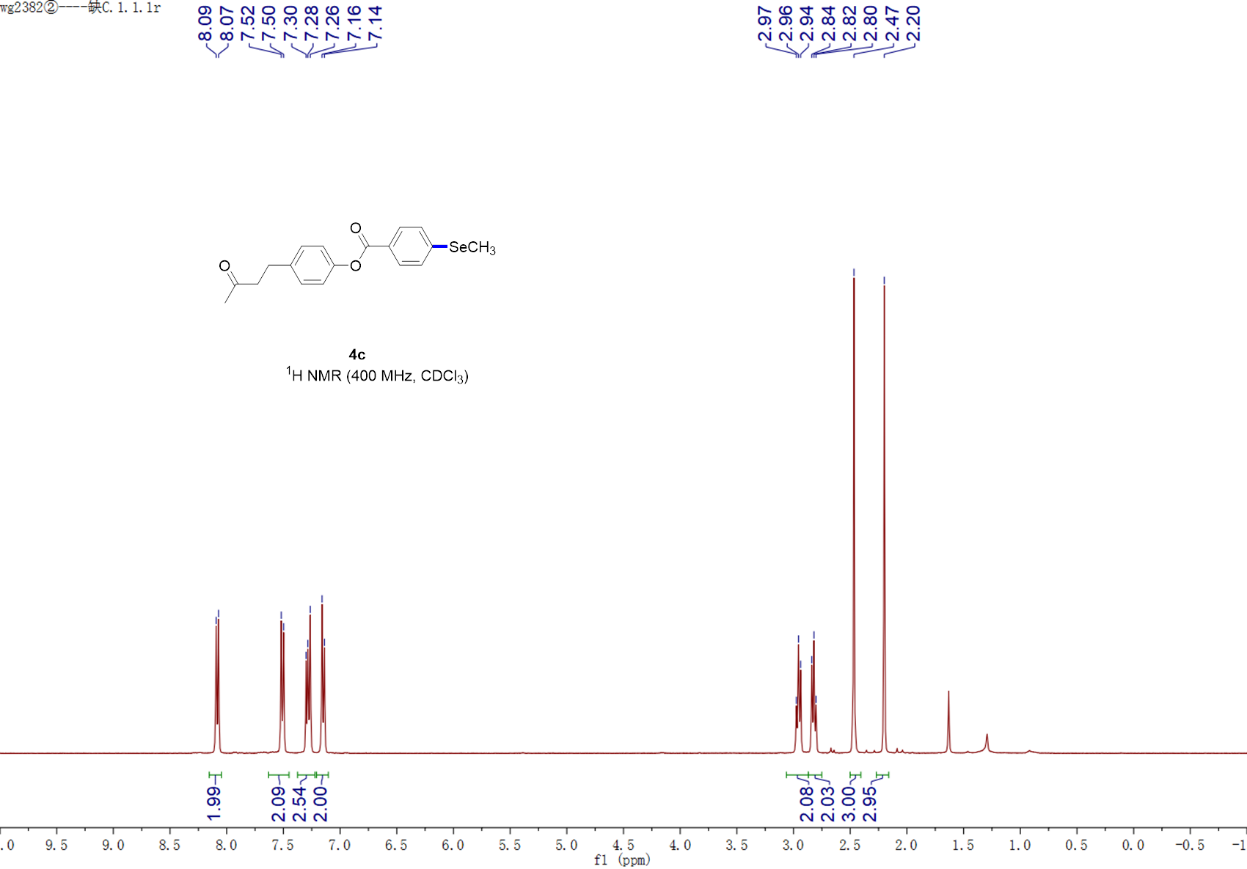
**

**^13^C{^1^H} NMR (100 MHz, Chloroform-*d*) spectrum of 4c**

**
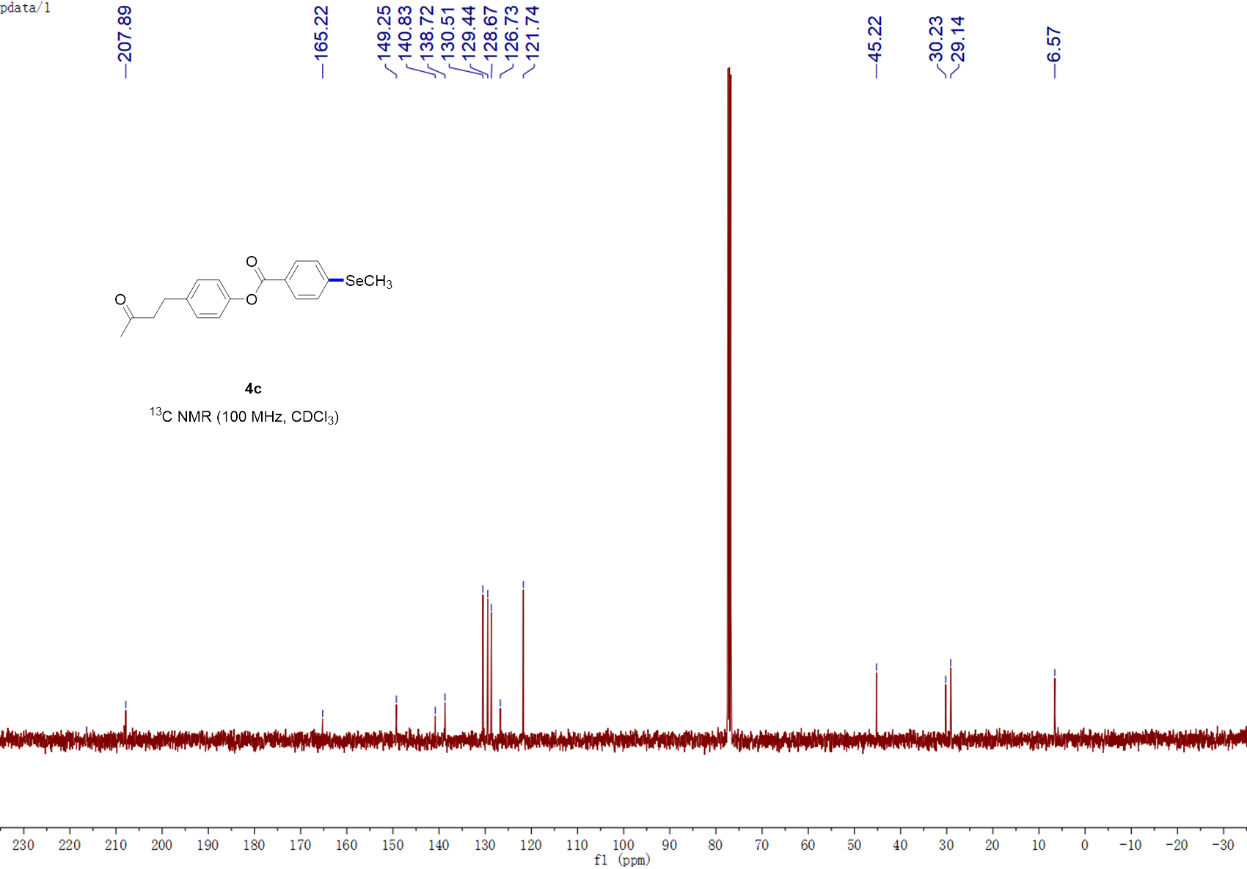
**

**^1^H NMR (400 MHz, Chloroform-*d*) spectrum of 4d**

**
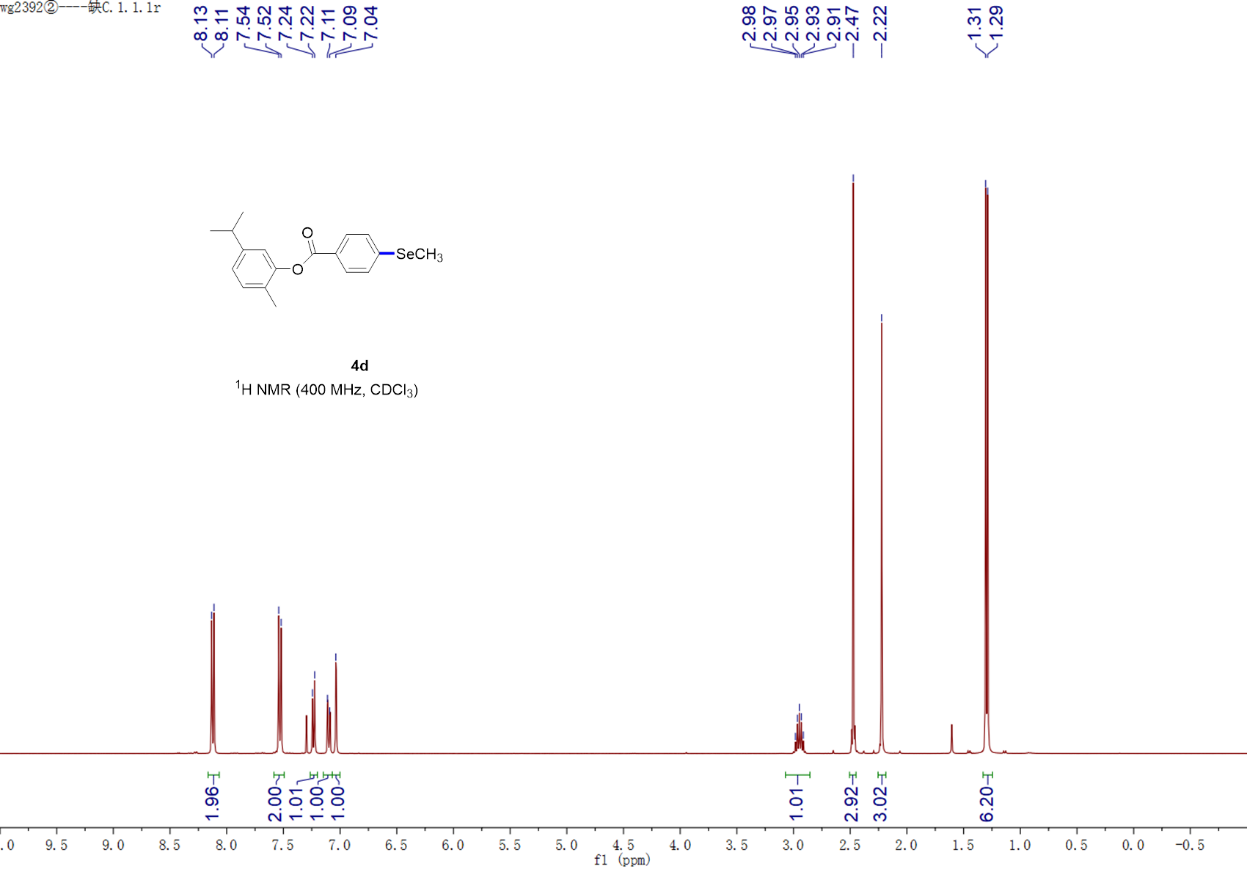
**

**^13^C{^1^H} NMR (100 MHz, Chloroform-*d*) spectrum of 4d**

**
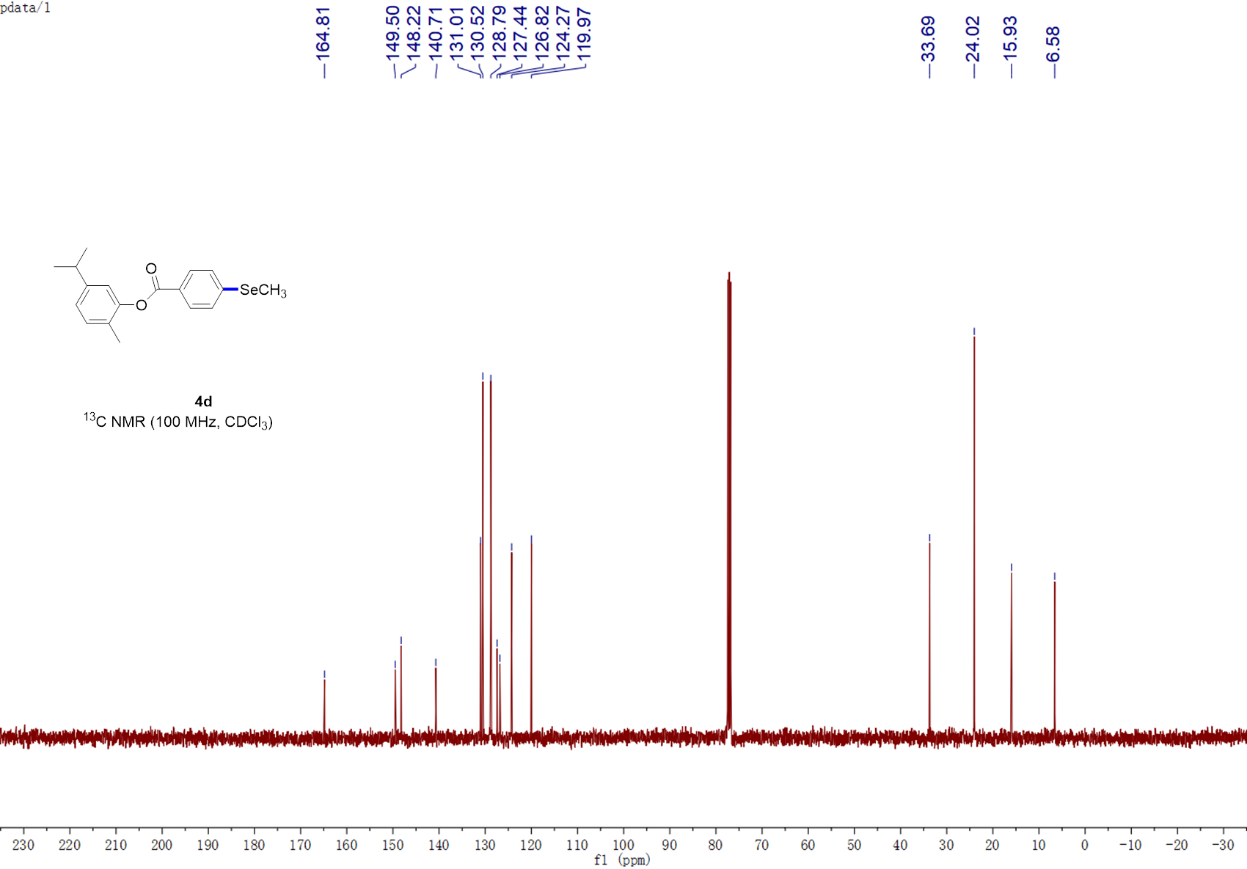
**

**^1^H NMR (400 MHz, Chloroform-*d*) spectrum of 4e**

**
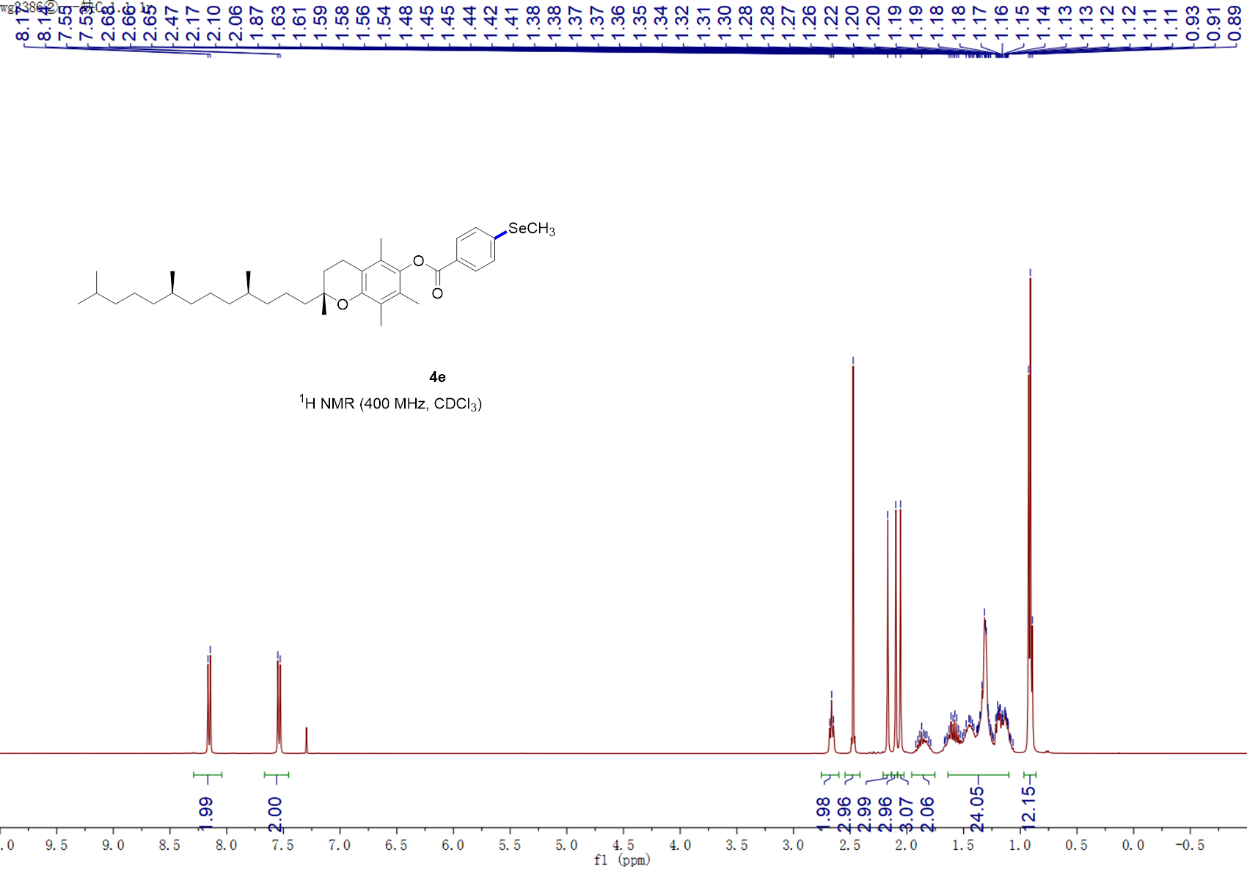
**

**^13^C{^1^H} NMR (100 MHz, Chloroform-*d*) spectrum of 4e**

**
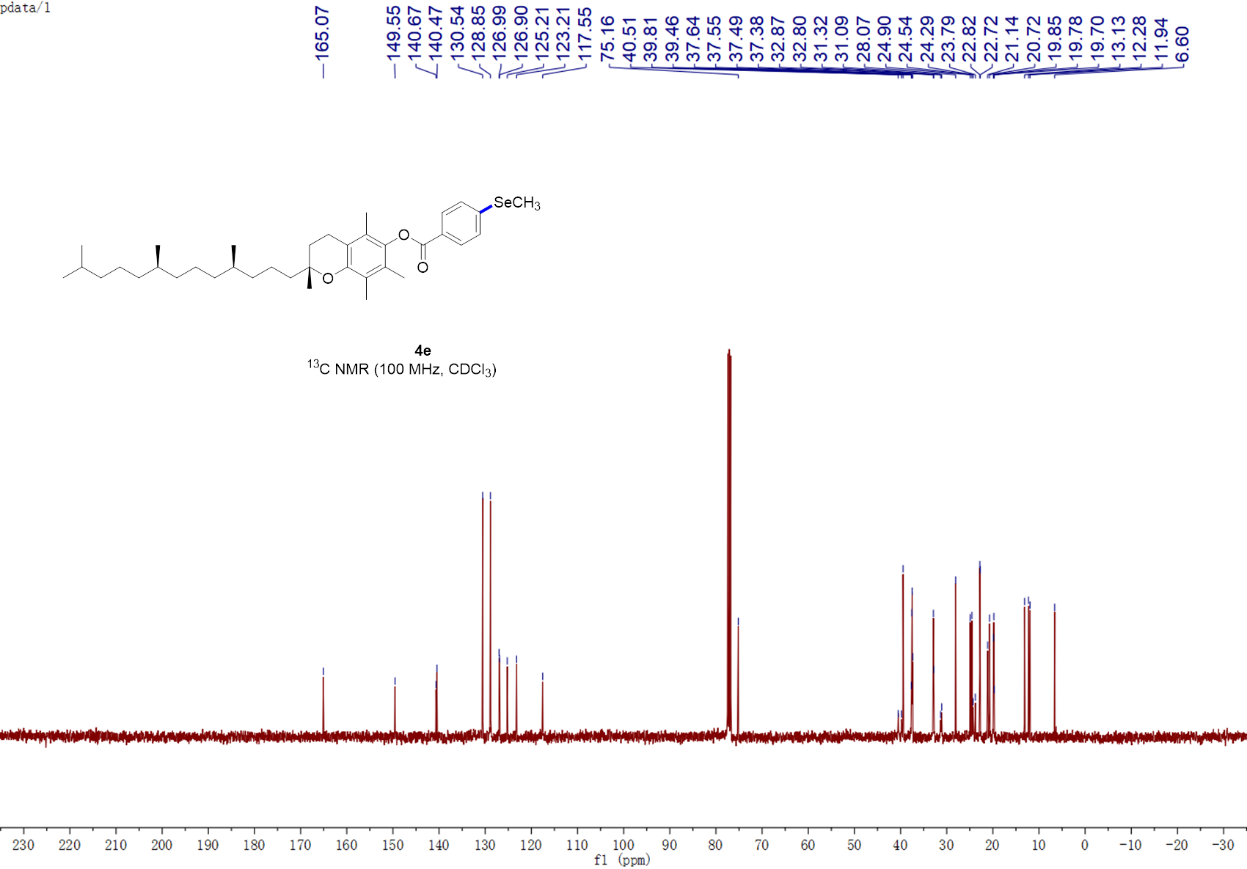
**

**^1^H NMR (400 MHz, Chloroform-*d*) spectrum of 4f**

**
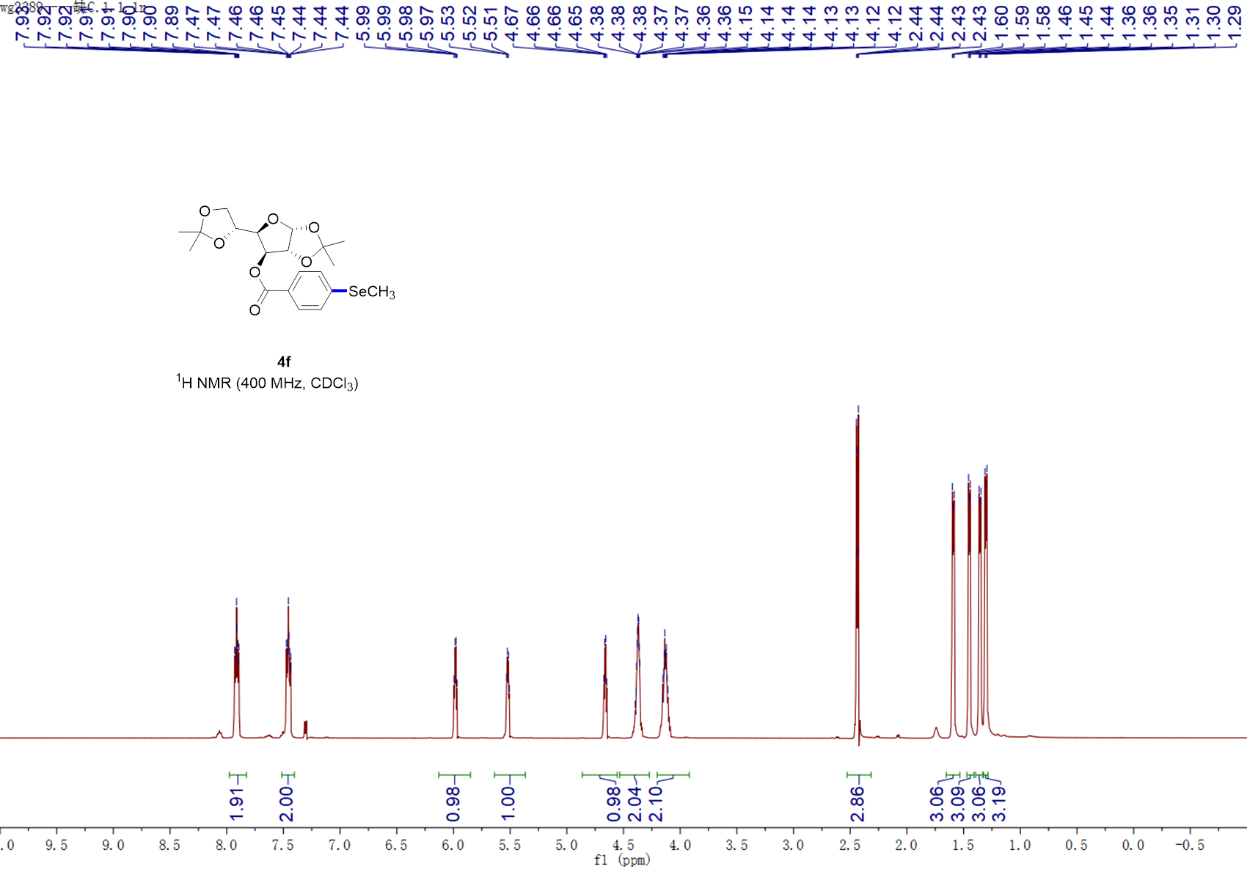
**

**^13^C{^1^H} NMR (100 MHz, Chloroform-*d*) spectrum of 4f**

**
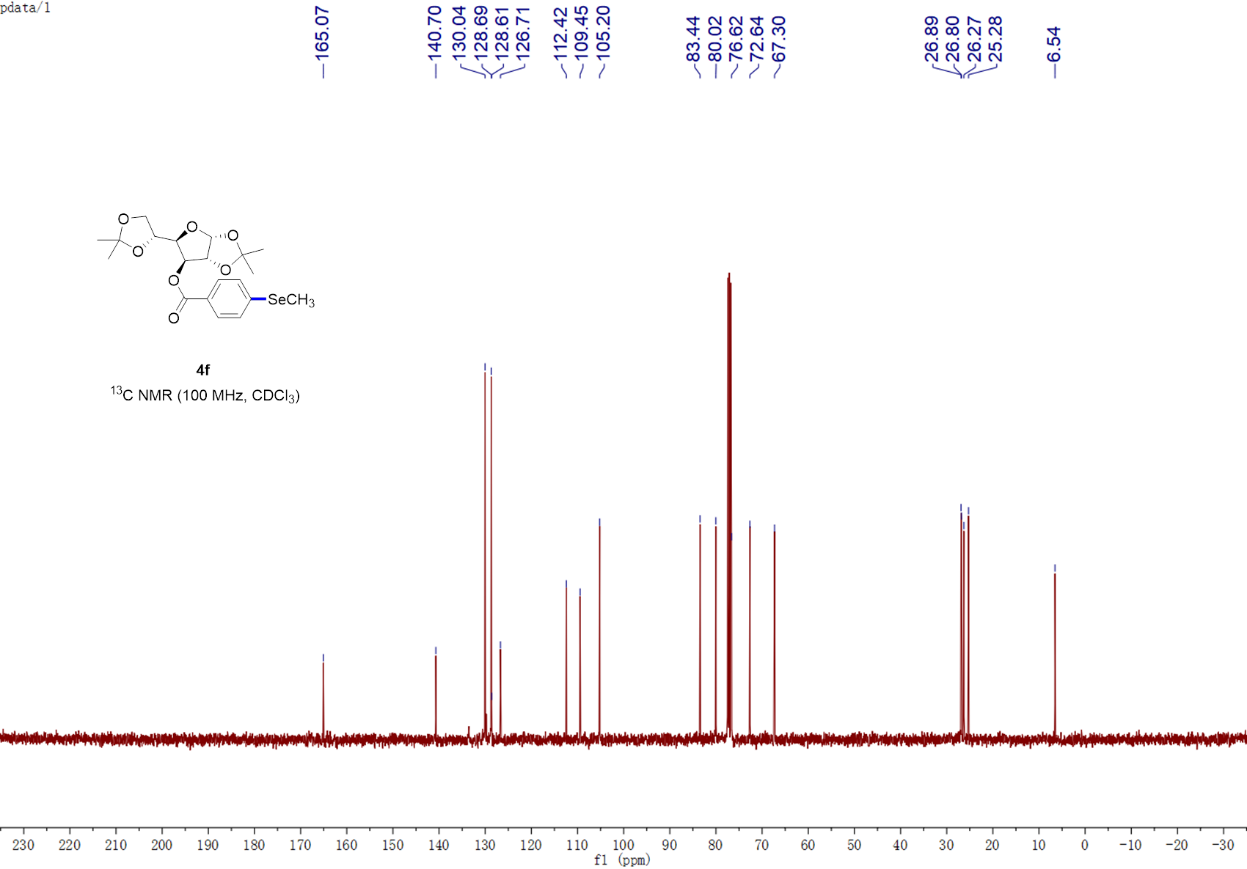
**

**^1^H NMR (400 MHz, Chloroform-*d*) spectrum of 4g**

**
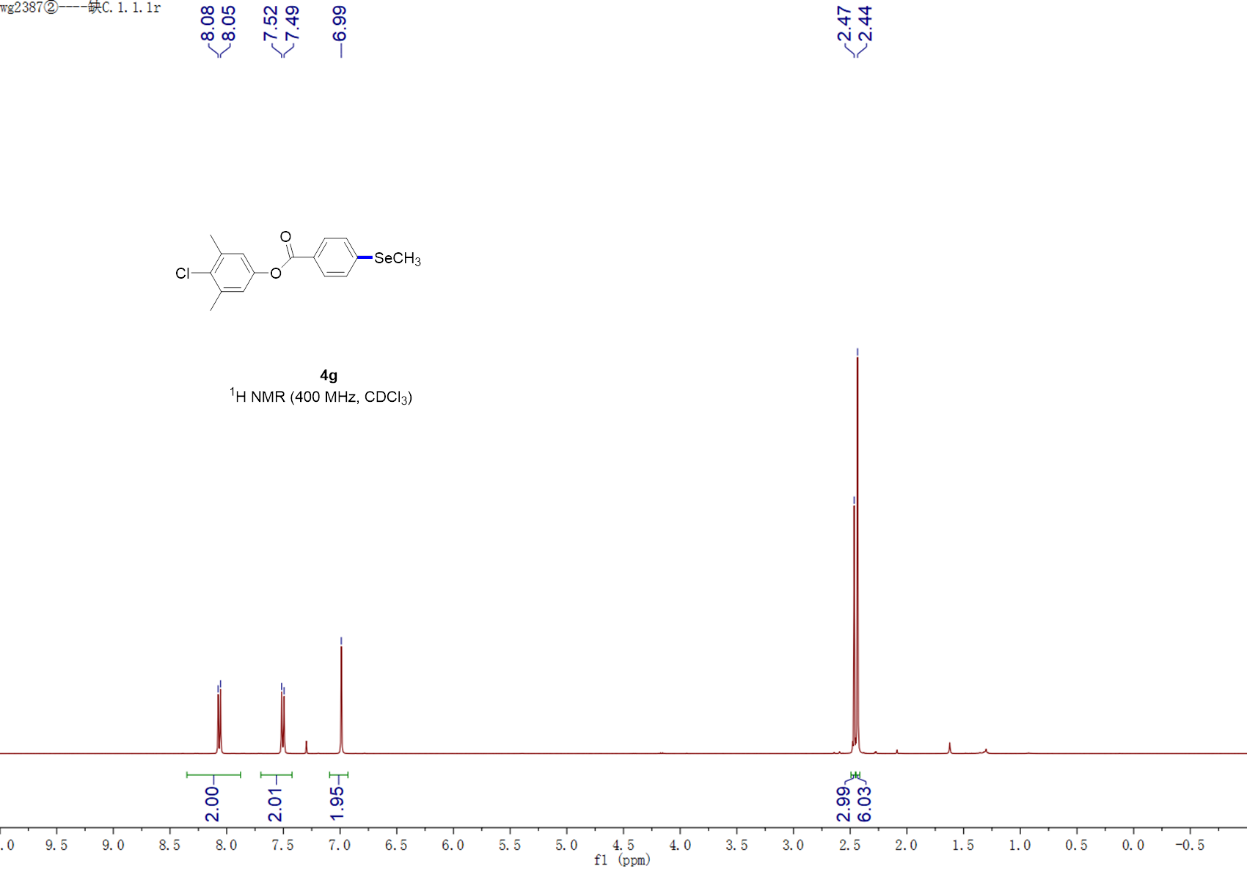
**

**^13^C{^1^H} NMR (100 MHz, Chloroform-*d*) spectrum of 4g**

**
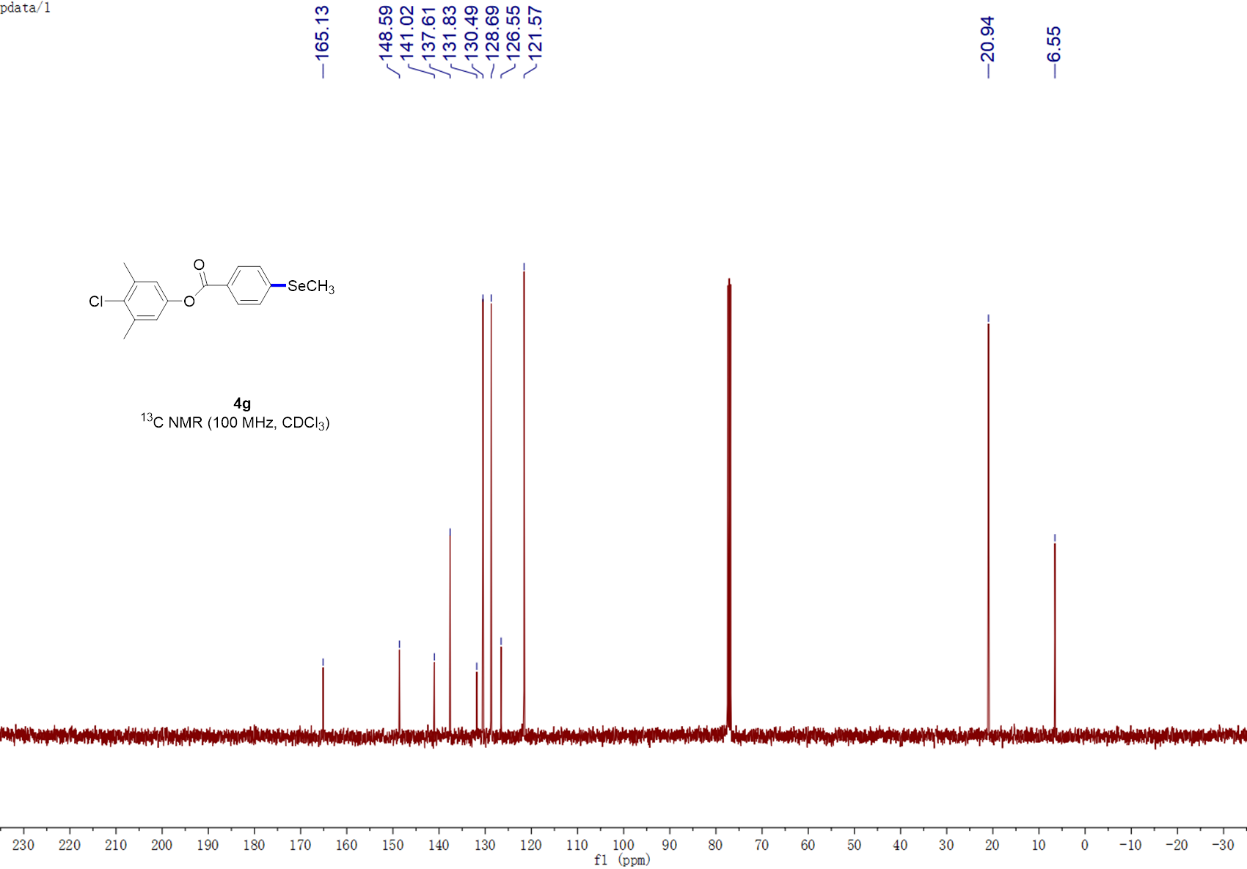
**

**^1^H NMR (400 MHz, Chloroform-*d*) spectrum of 4h**

**
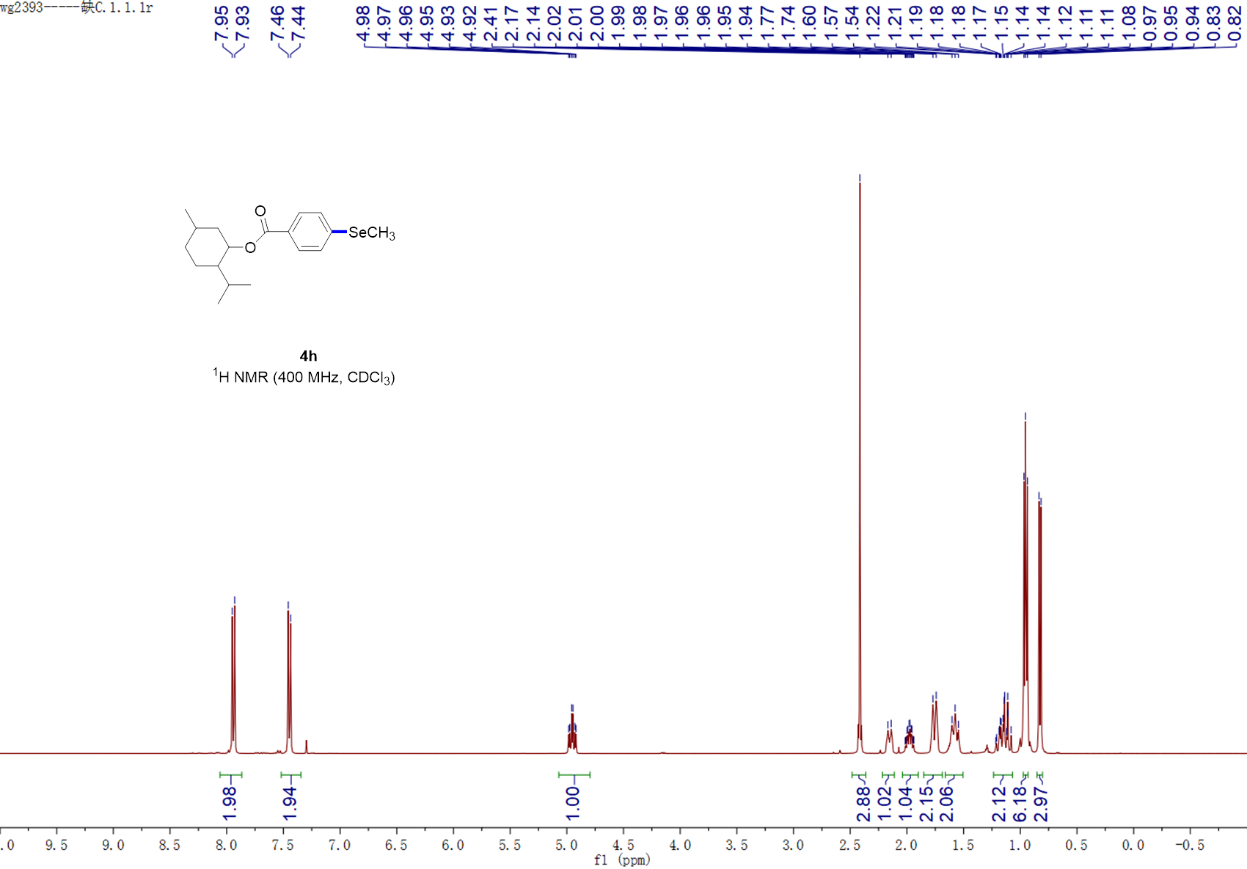
**

**^13^C{^1^H} NMR (100 MHz, Chloroform-*d*) spectrum of 4h**

**
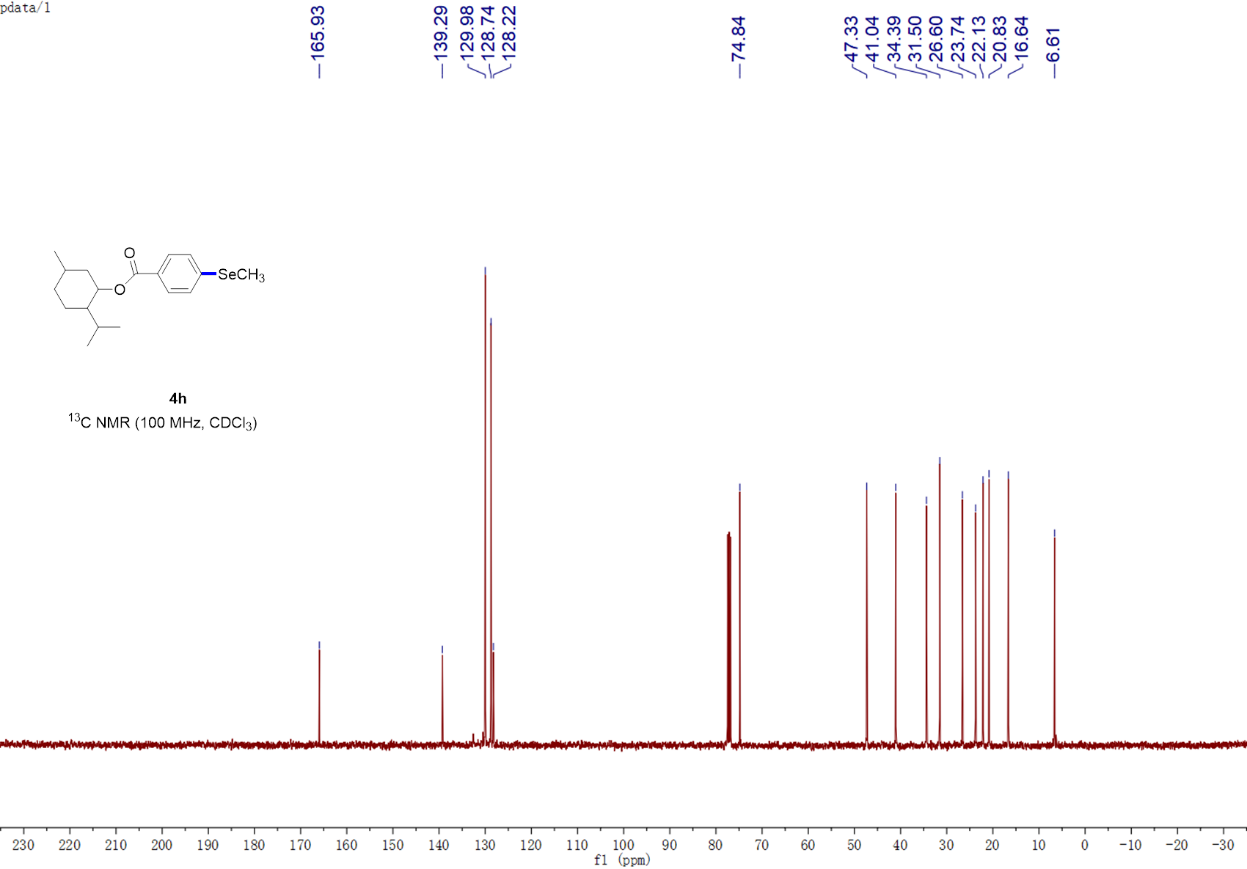
**

**^1^H NMR (400 MHz, Chloroform-*d*) spectrum of 5a**

**
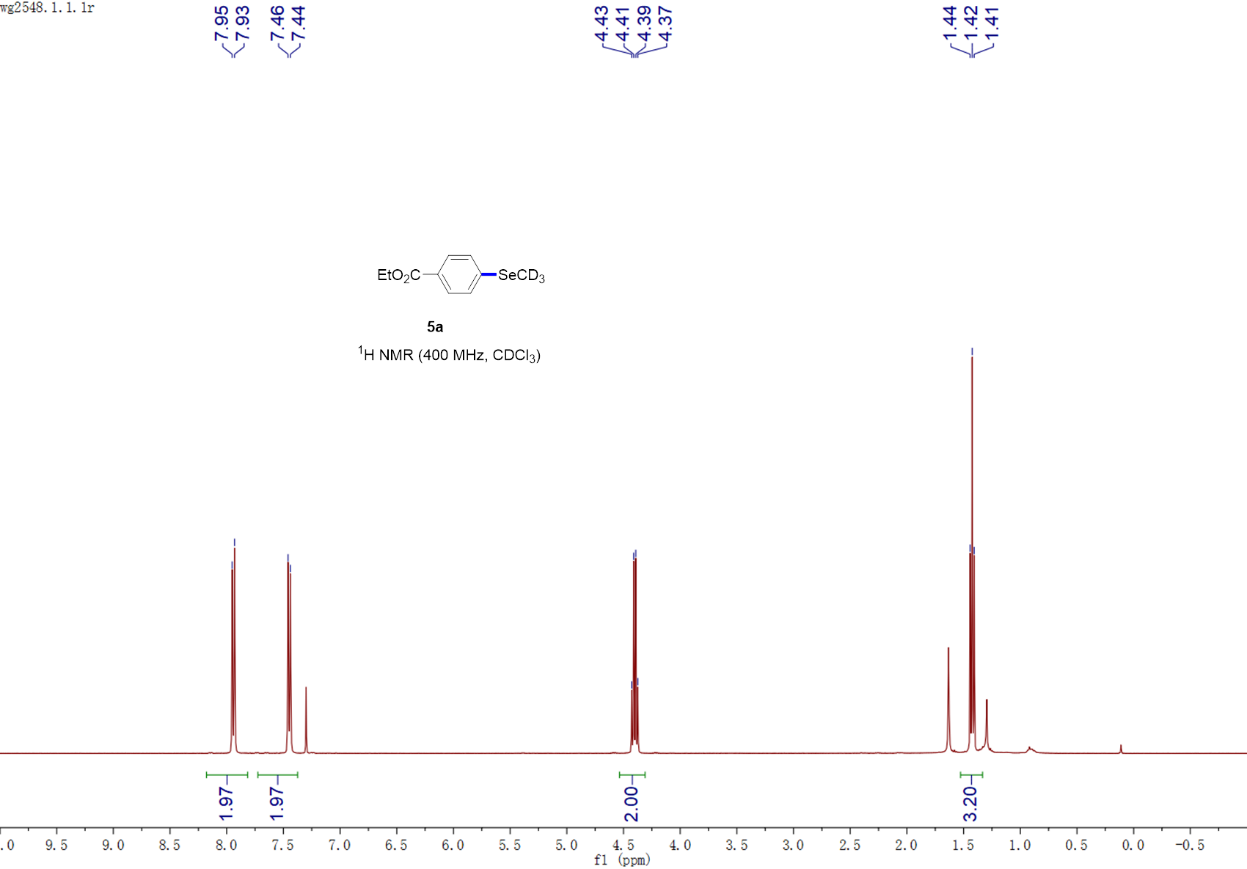
**

**^13^C{^1^H} NMR (100 MHz, Chloroform-*d*) spectrum of 5a**

**
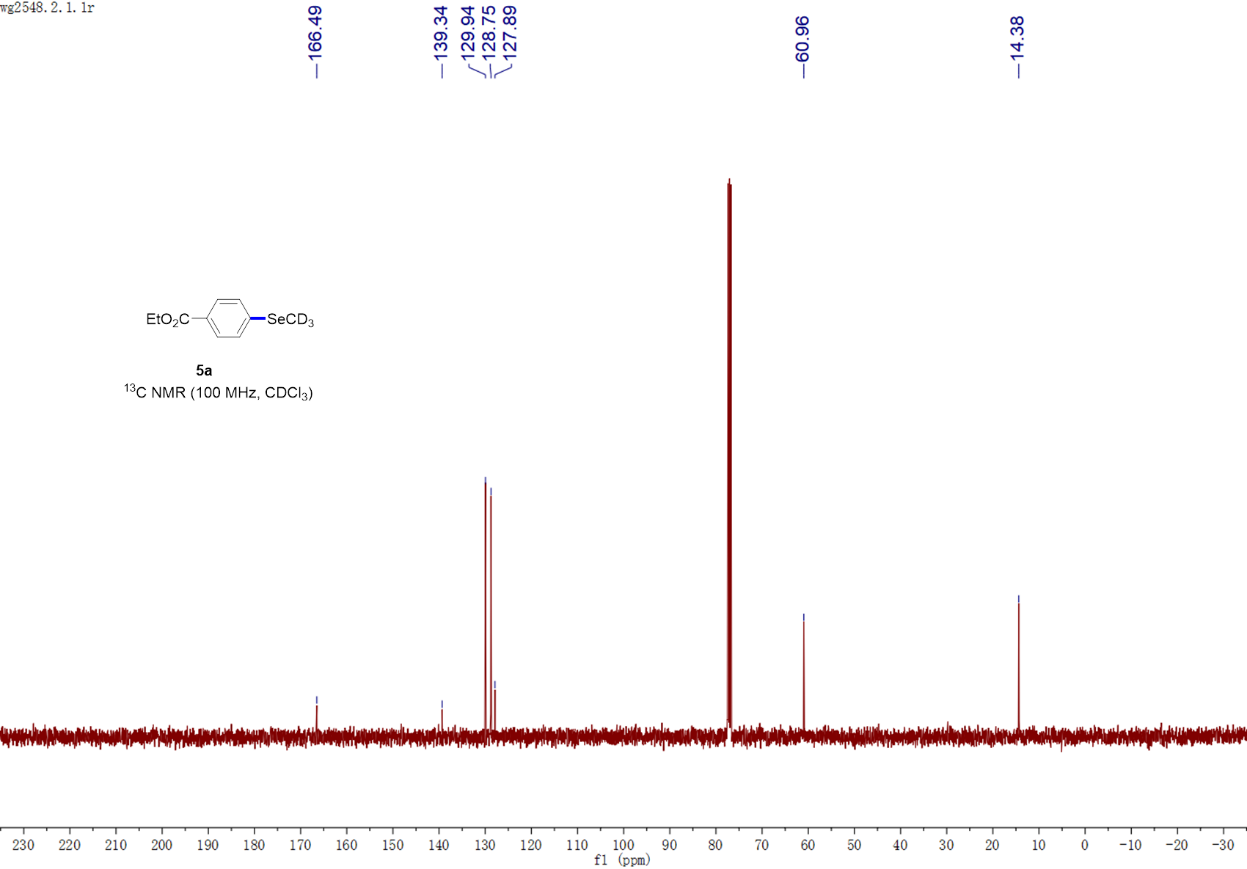
**

**^1^H NMR (400 MHz, Chloroform-*d*) spectrum of 5b**

**
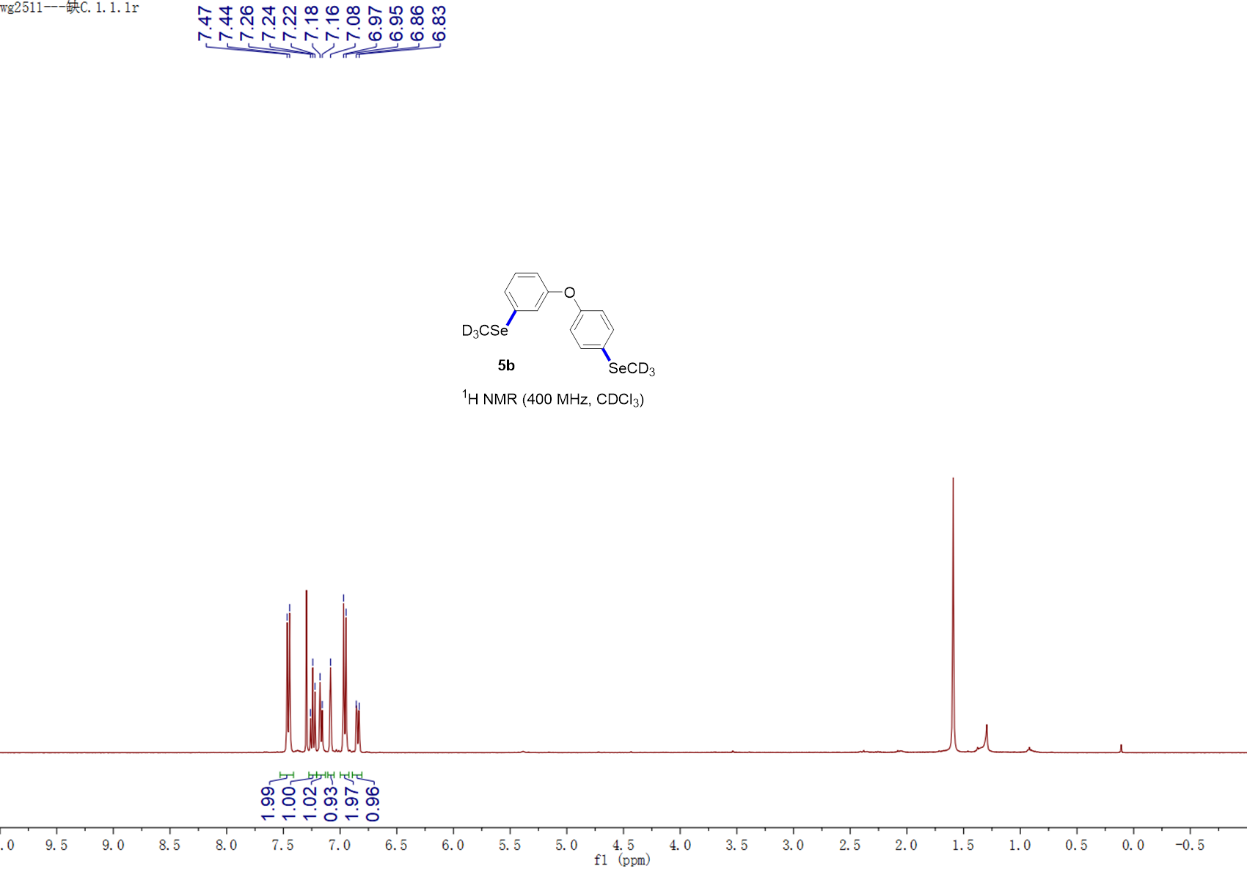
**

**^13^C{^1^H} NMR (100 MHz, Chloroform-*d*) spectrum of 5b**

**
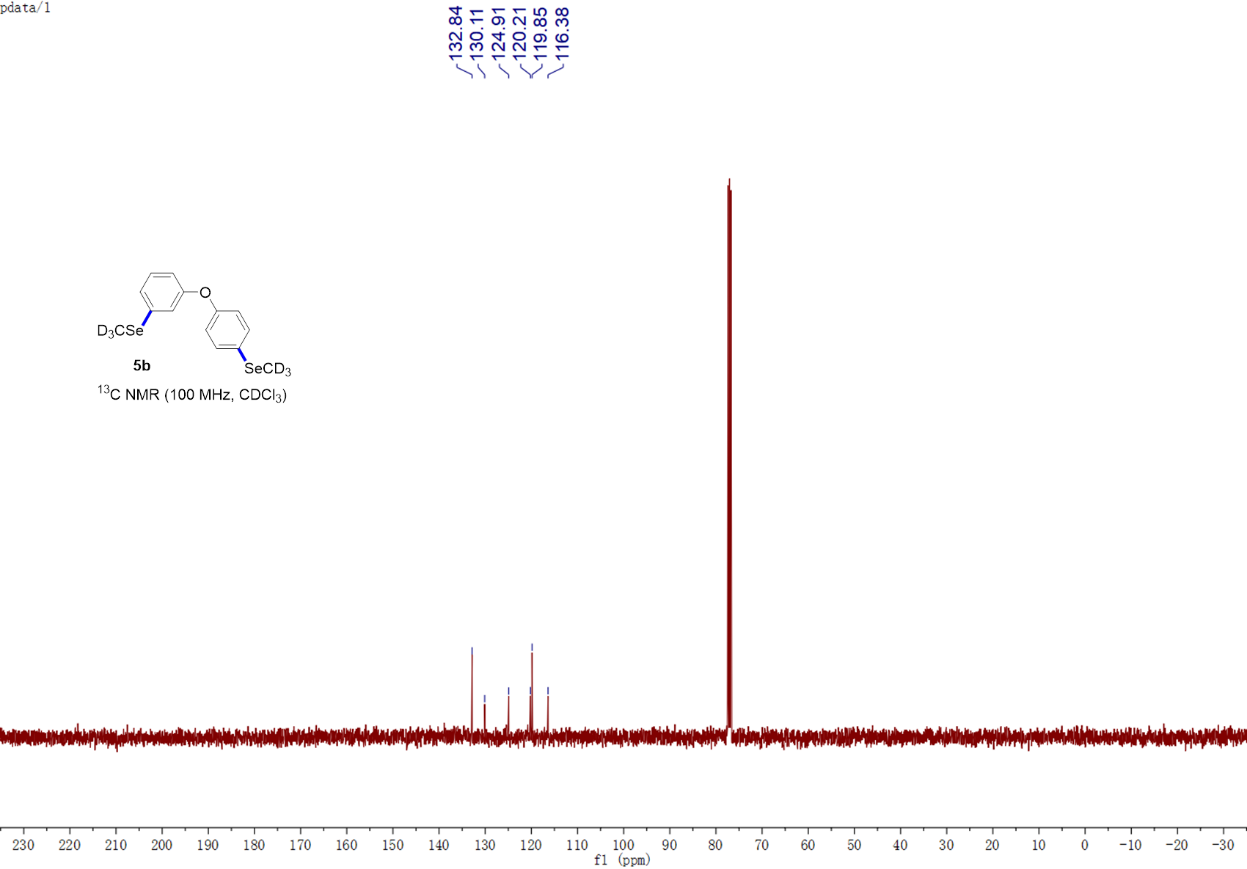
**

**^1^H NMR (400 MHz, Chloroform-*d*) spectrum of 5c**

**
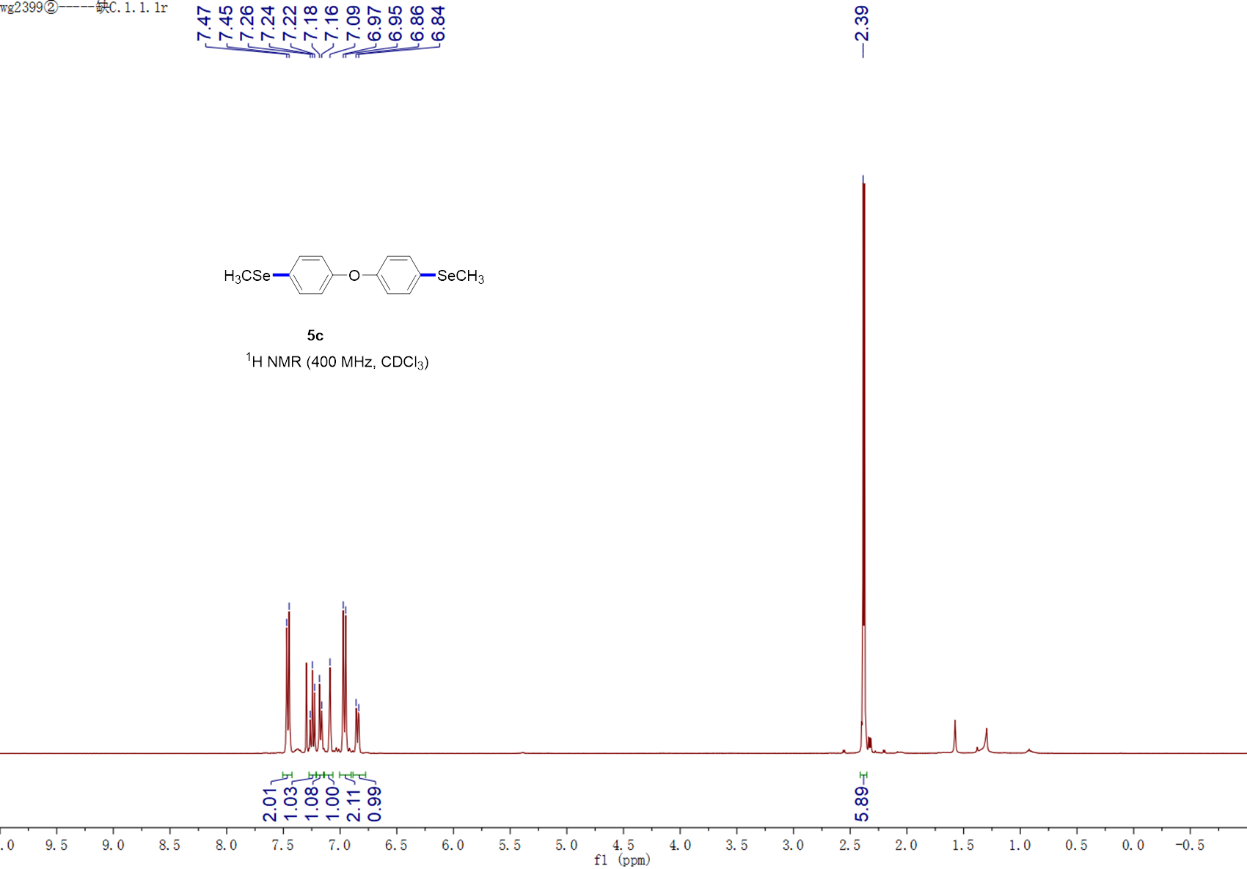
**

**^13^C{^1^H} NMR (100 MHz, Chloroform-*d*) spectrum of 5c**

**
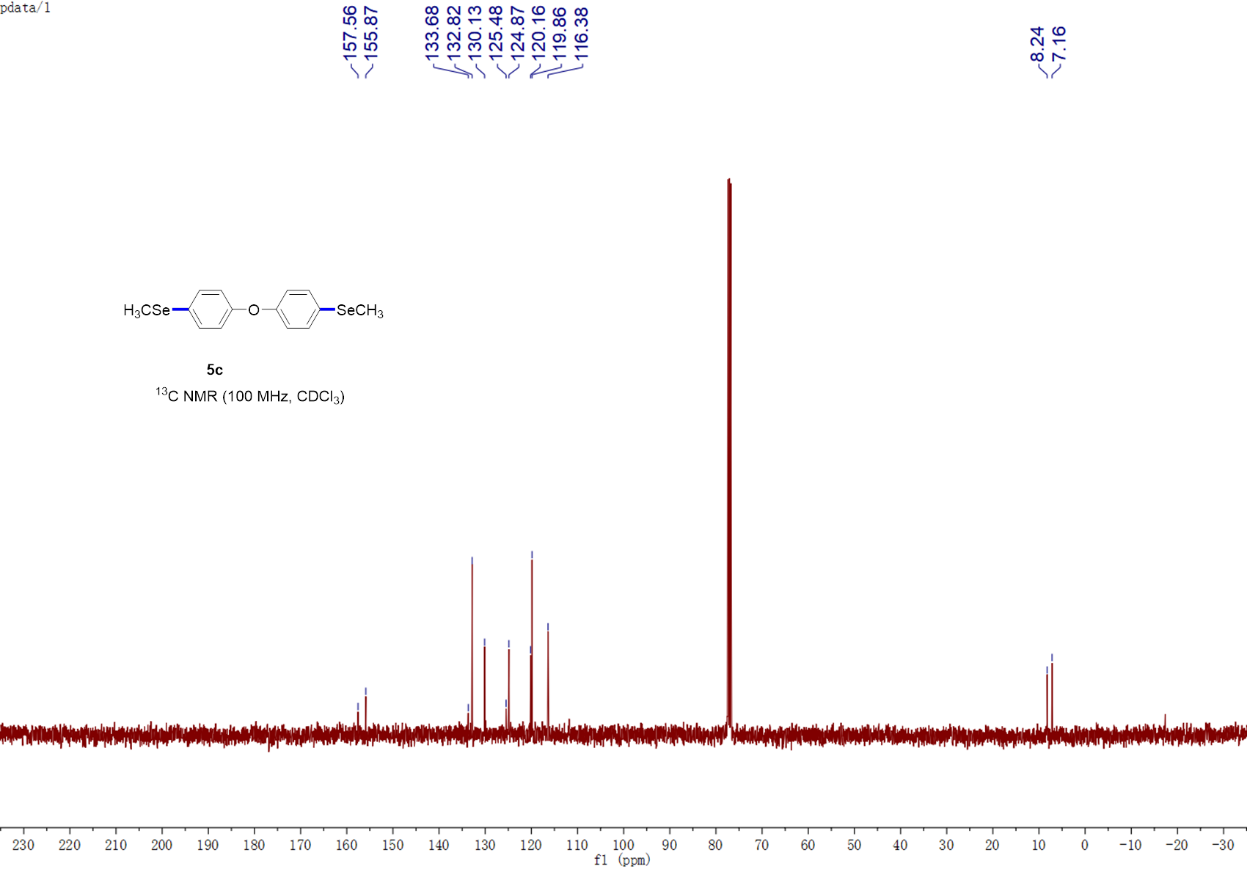
**

**^1^H NMR (400 MHz, Chloroform-*d*) spectrum of 5d**

**
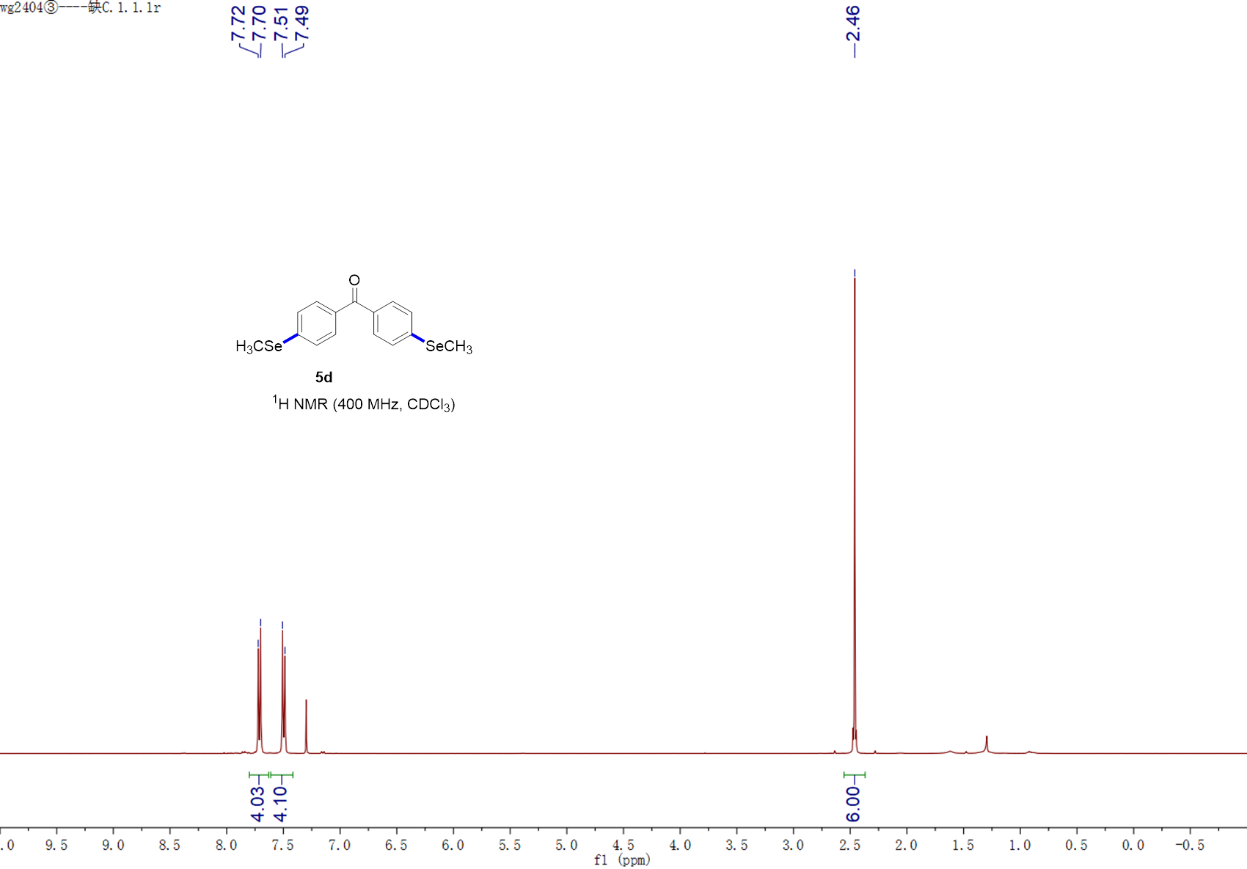
**

**^13^C{^1^H} NMR (100 MHz, Chloroform-*d*) spectrum of 5d**

**
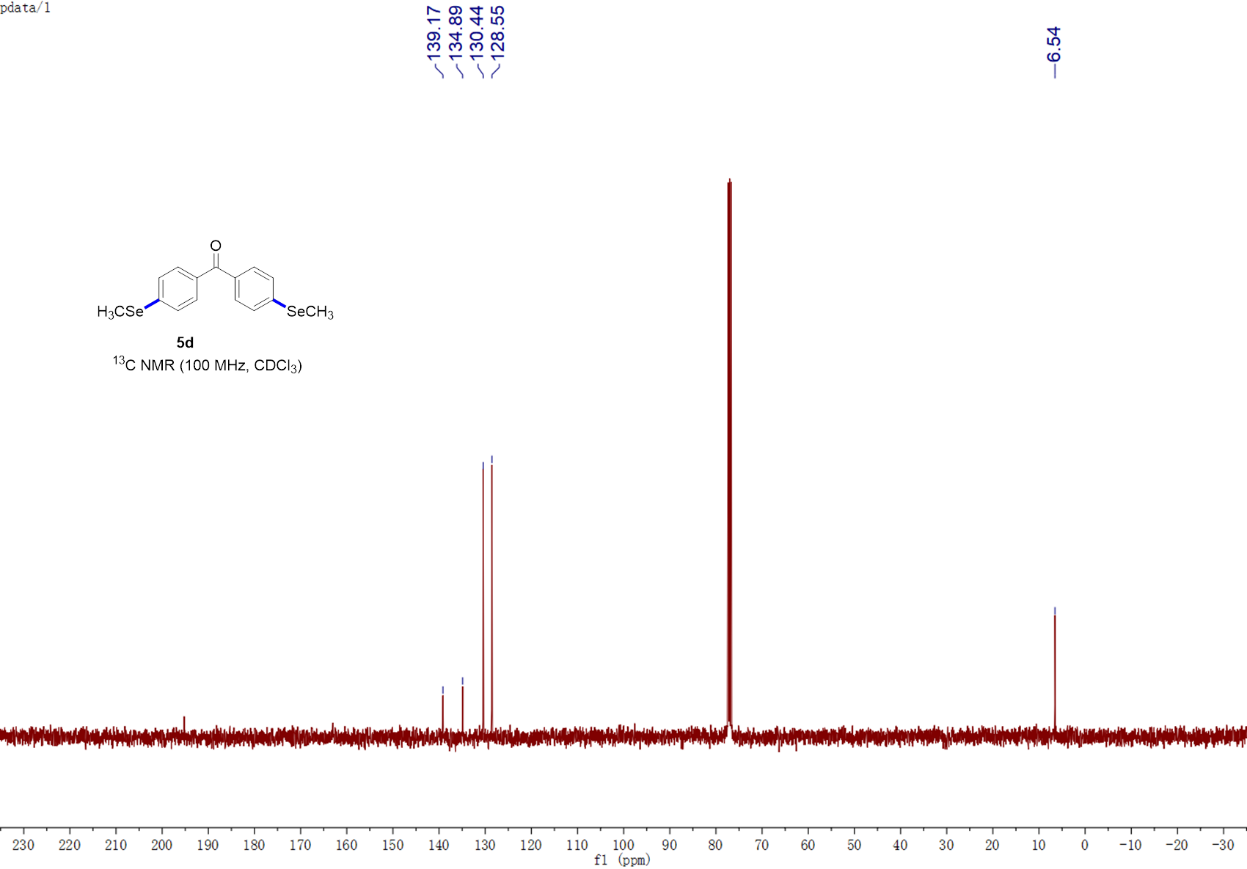
**

**^1^H NMR (400 MHz, Chloroform-*d*) spectrum of 5e**

**
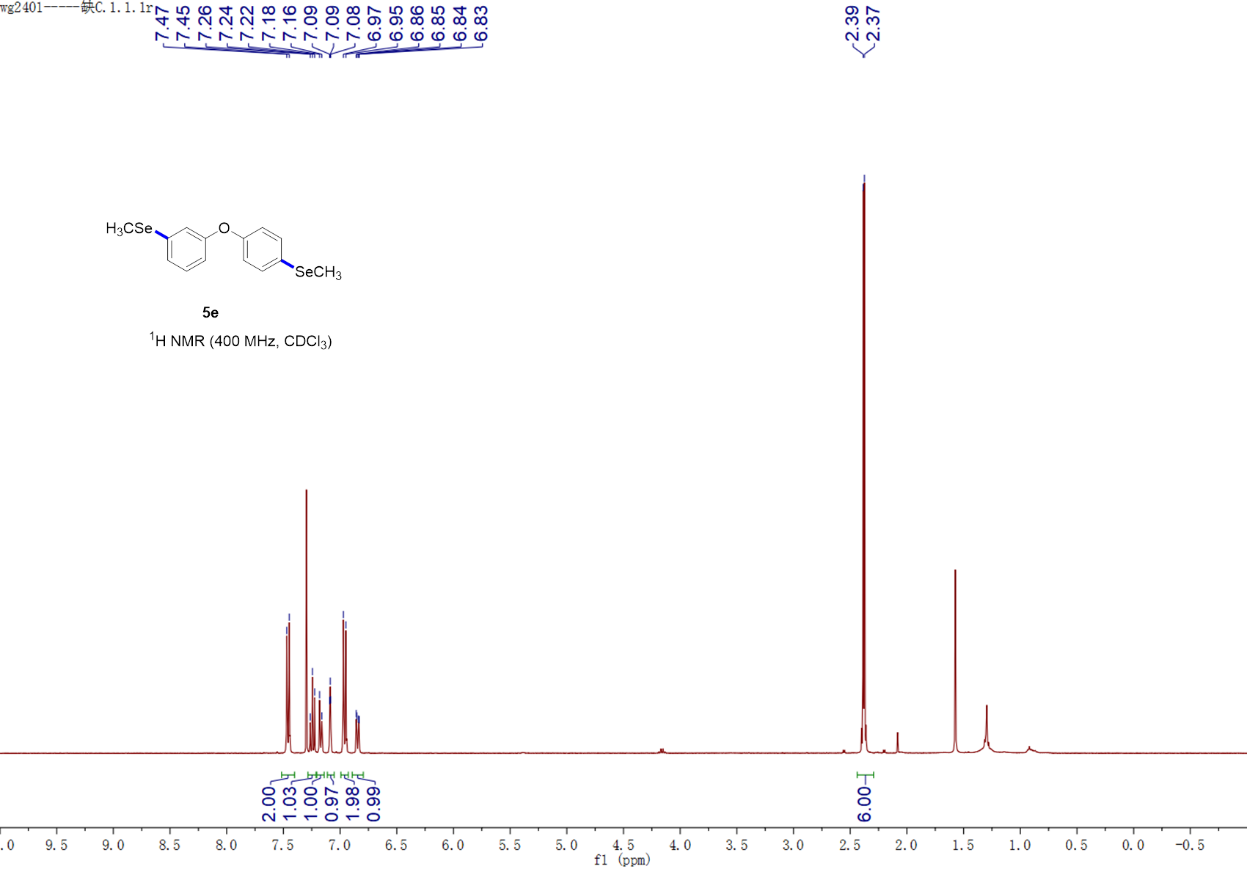
**

**^13^C{^1^H} NMR (100 MHz, Chloroform-*d*) spectrum of 5e**

**
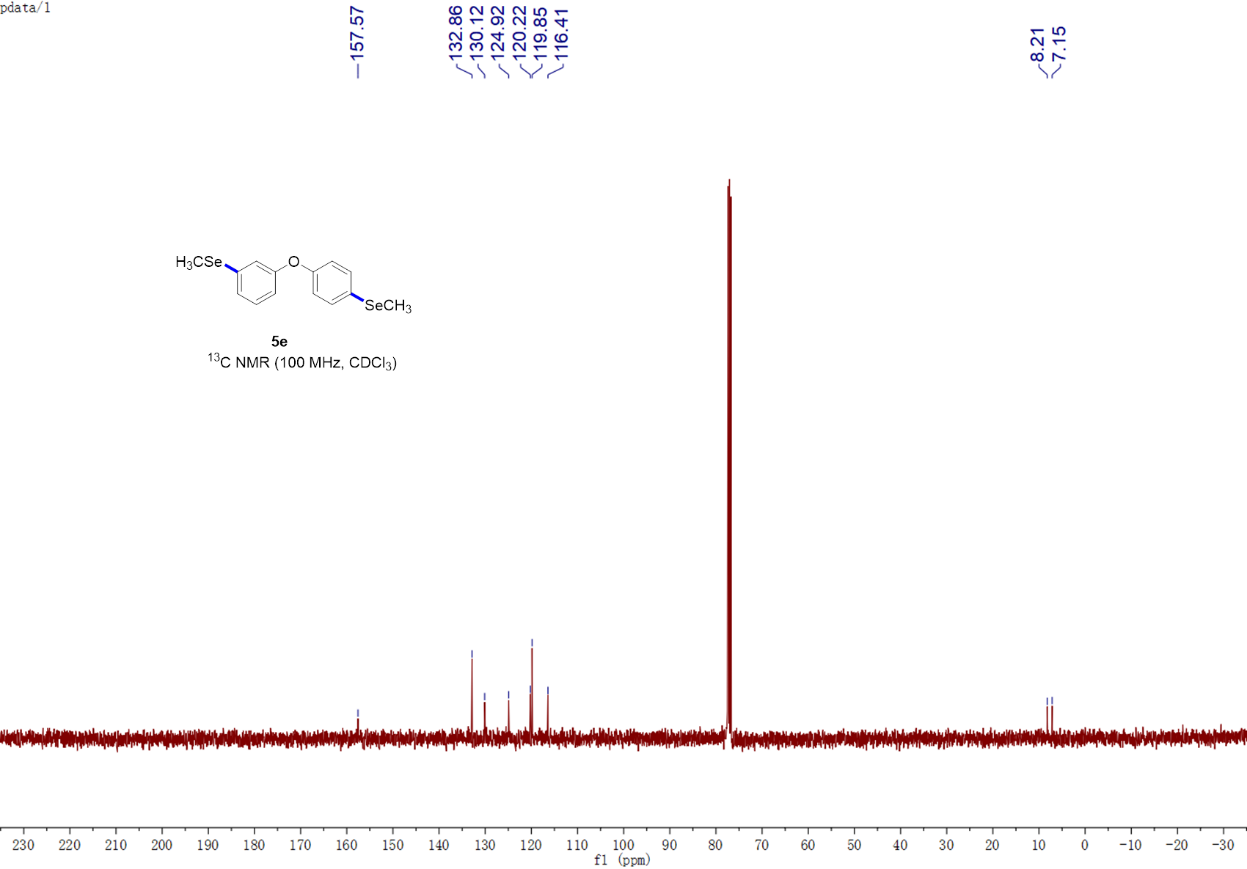
**

**^1^H NMR (400 MHz, Chloroform-*d*) spectrum of 5f**

**
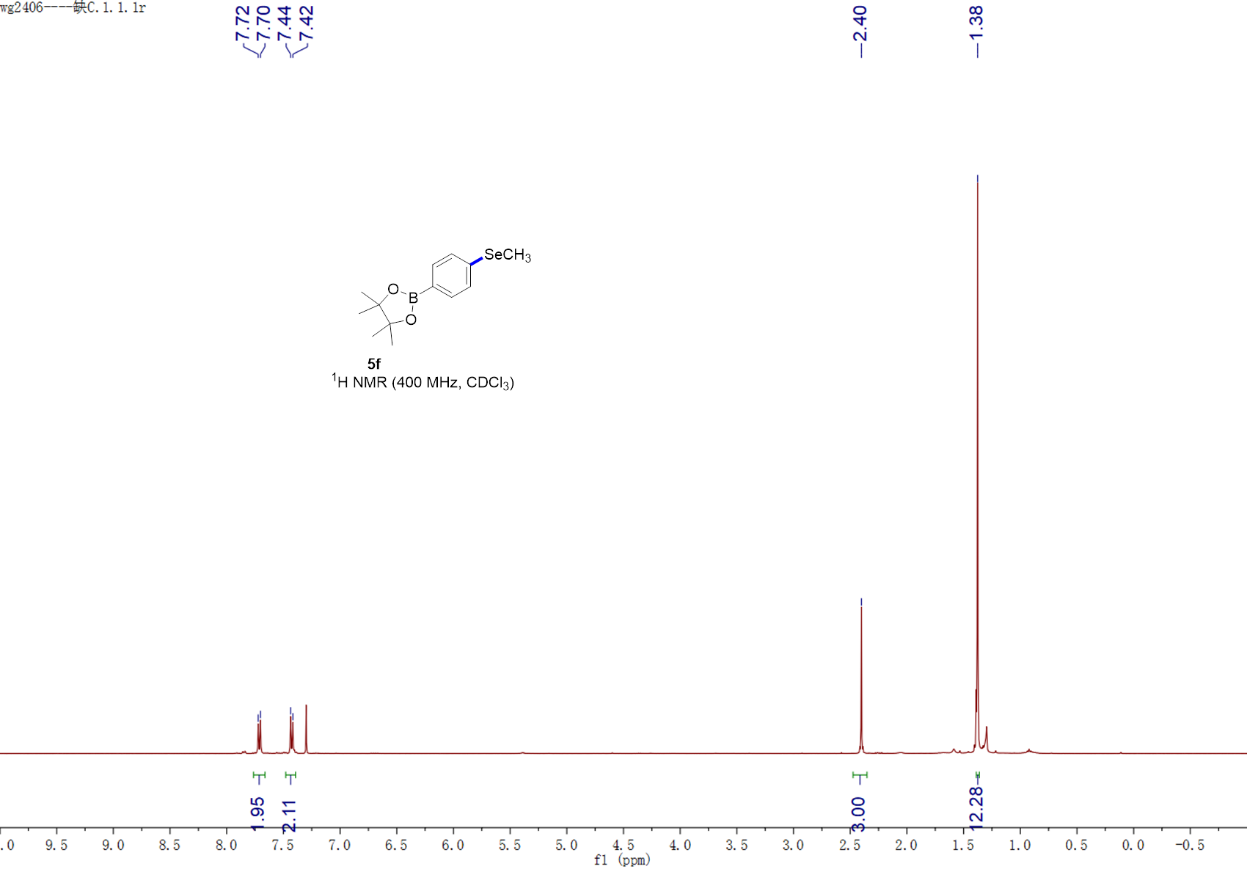
**

**^13^C{^1^H} NMR (100 MHz, Chloroform-*d*) spectrum of 5f**

**
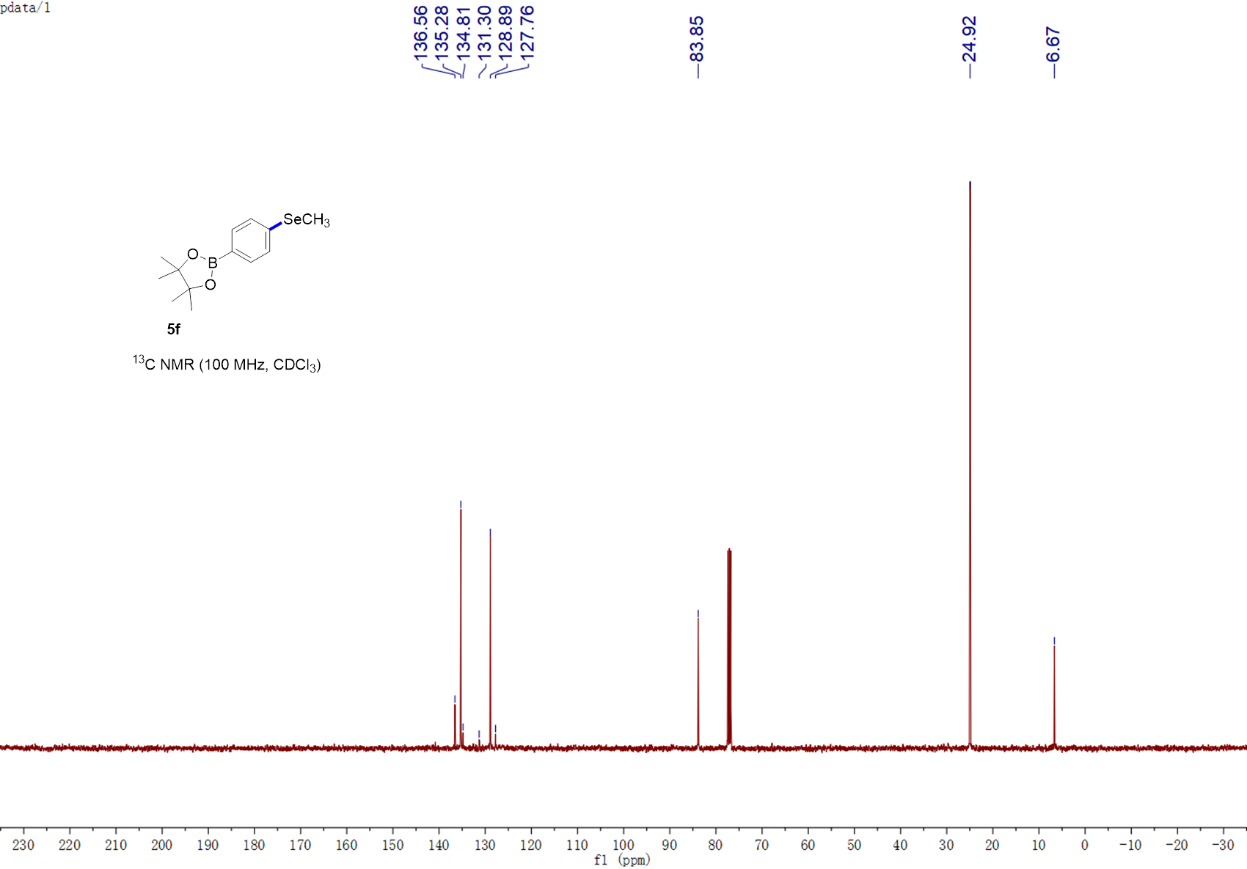
**

**^1^H NMR (400 MHz, Chloroform-*d*) spectrum of 5g**

**
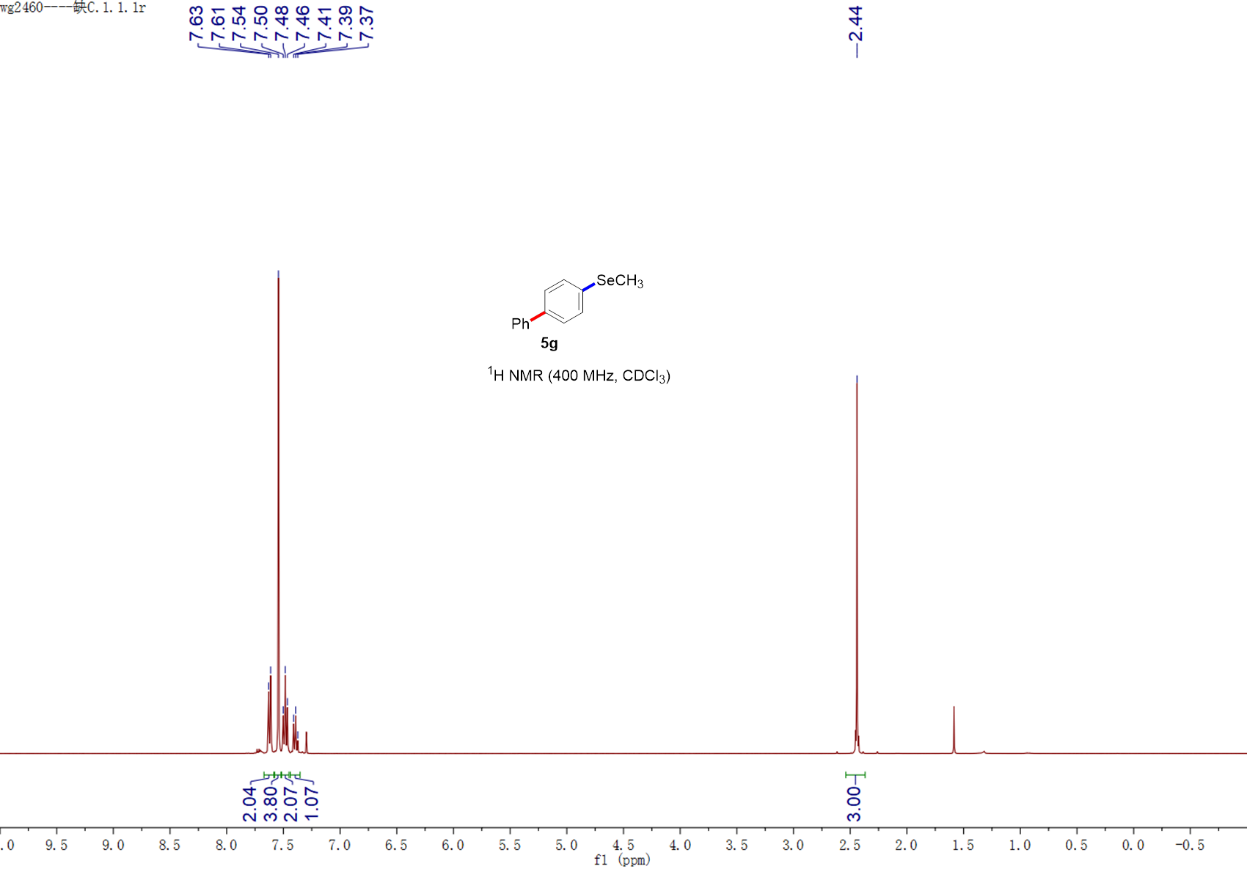
**

**^13^C{^1^H} NMR (100 MHz, Chloroform-*d*) spectrum of 5g**

**
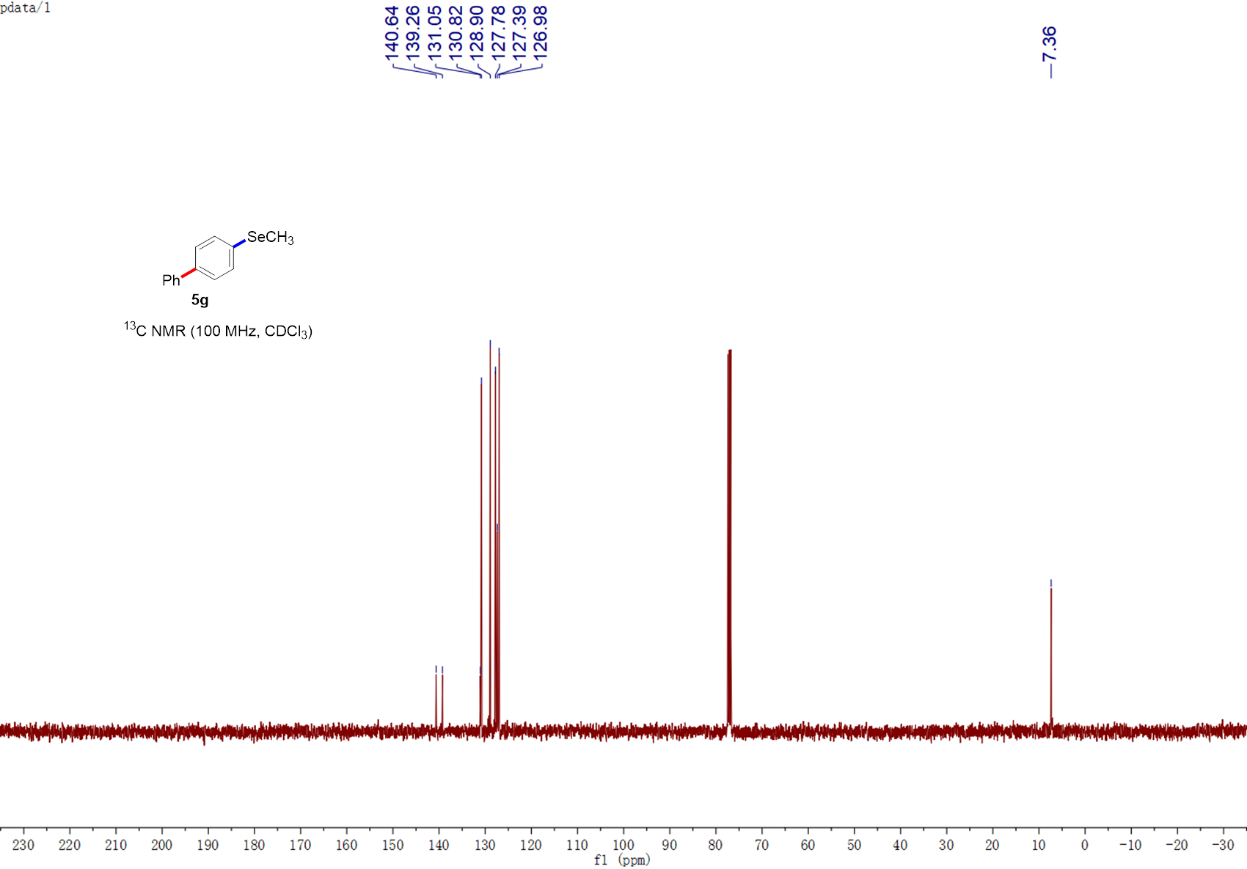
**

**^1^H NMR (400 MHz, Chloroform-*d*) spectrum of 5h**

**
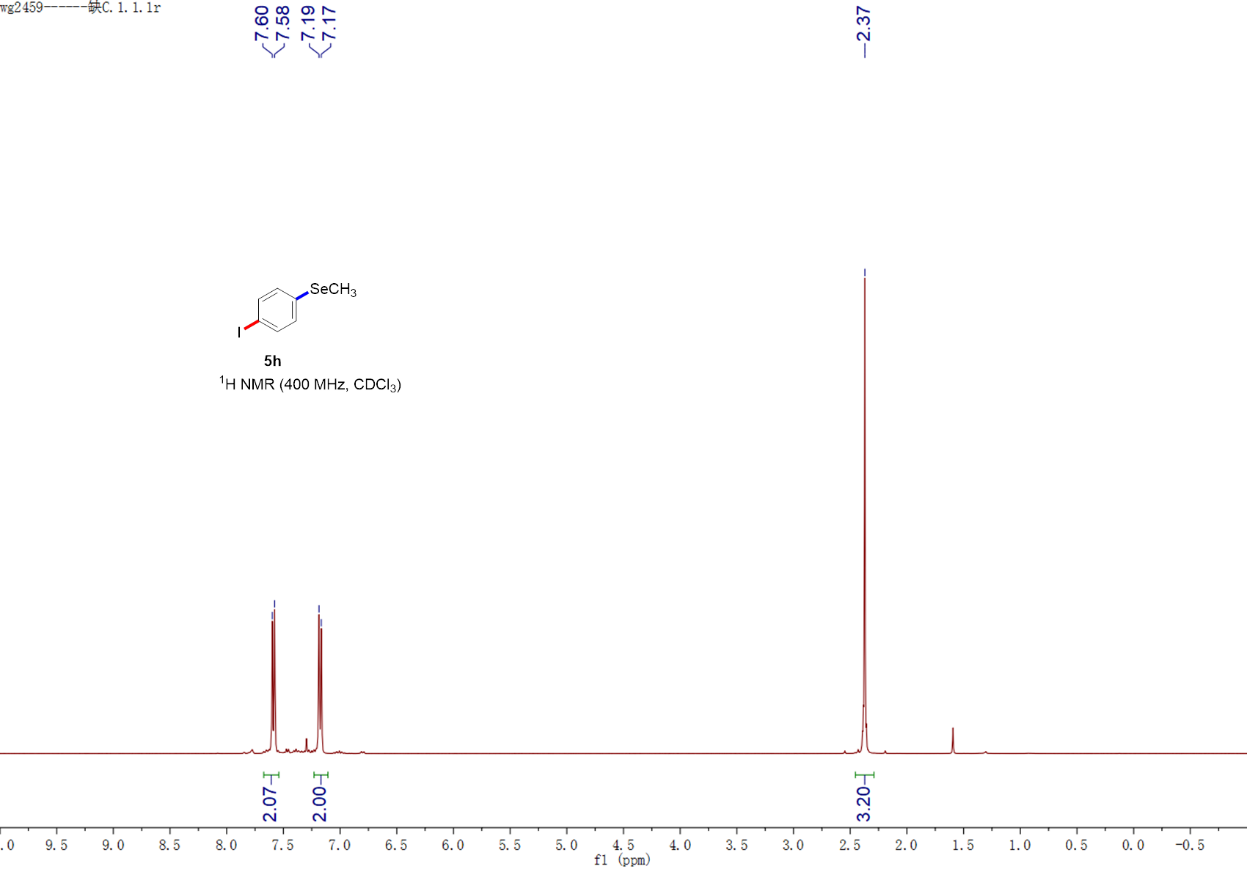
**

**^13^C{^1^H} NMR (100 MHz, Chloroform-*d*) spectrum of 5h**

**
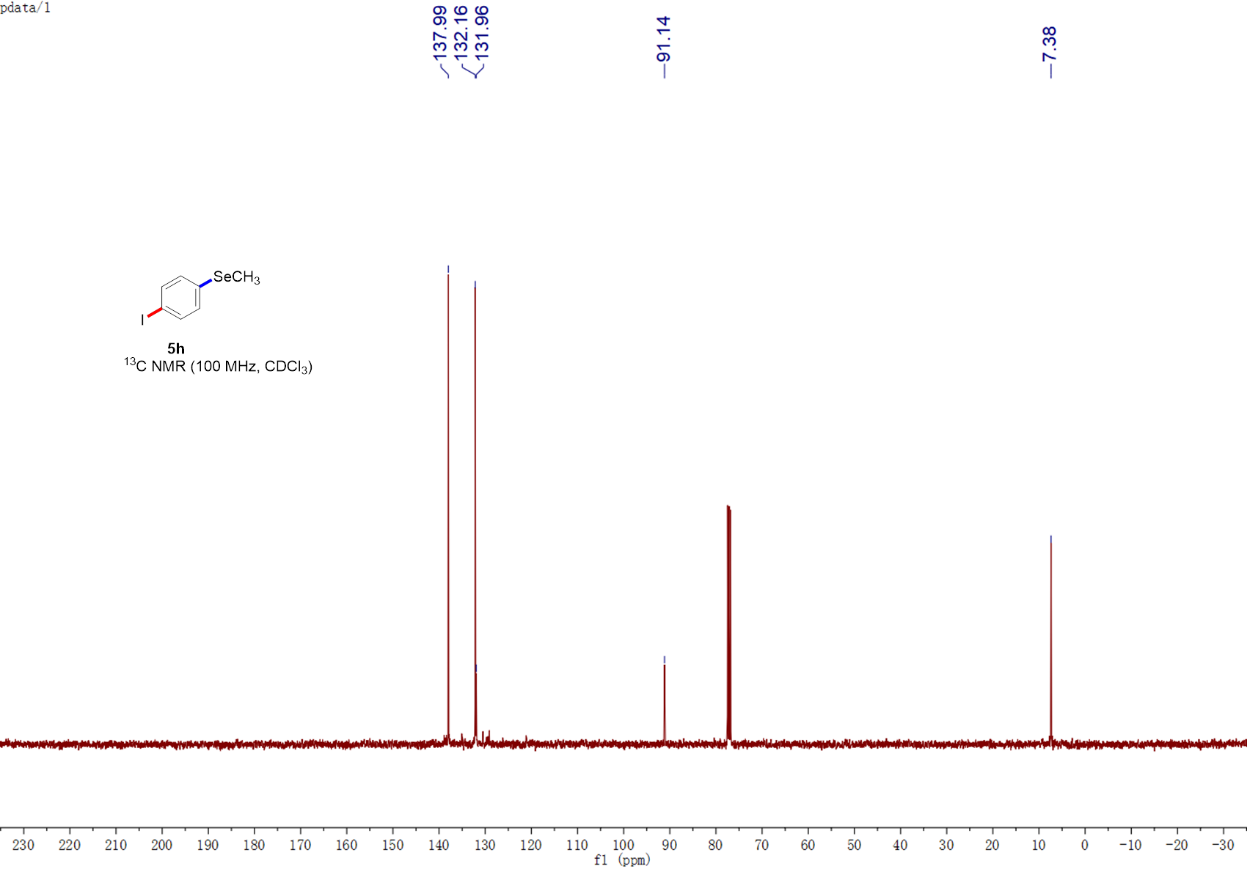
**

**^1^H NMR (400 MHz, Chloroform-*d*) spectrum of 5i**

**
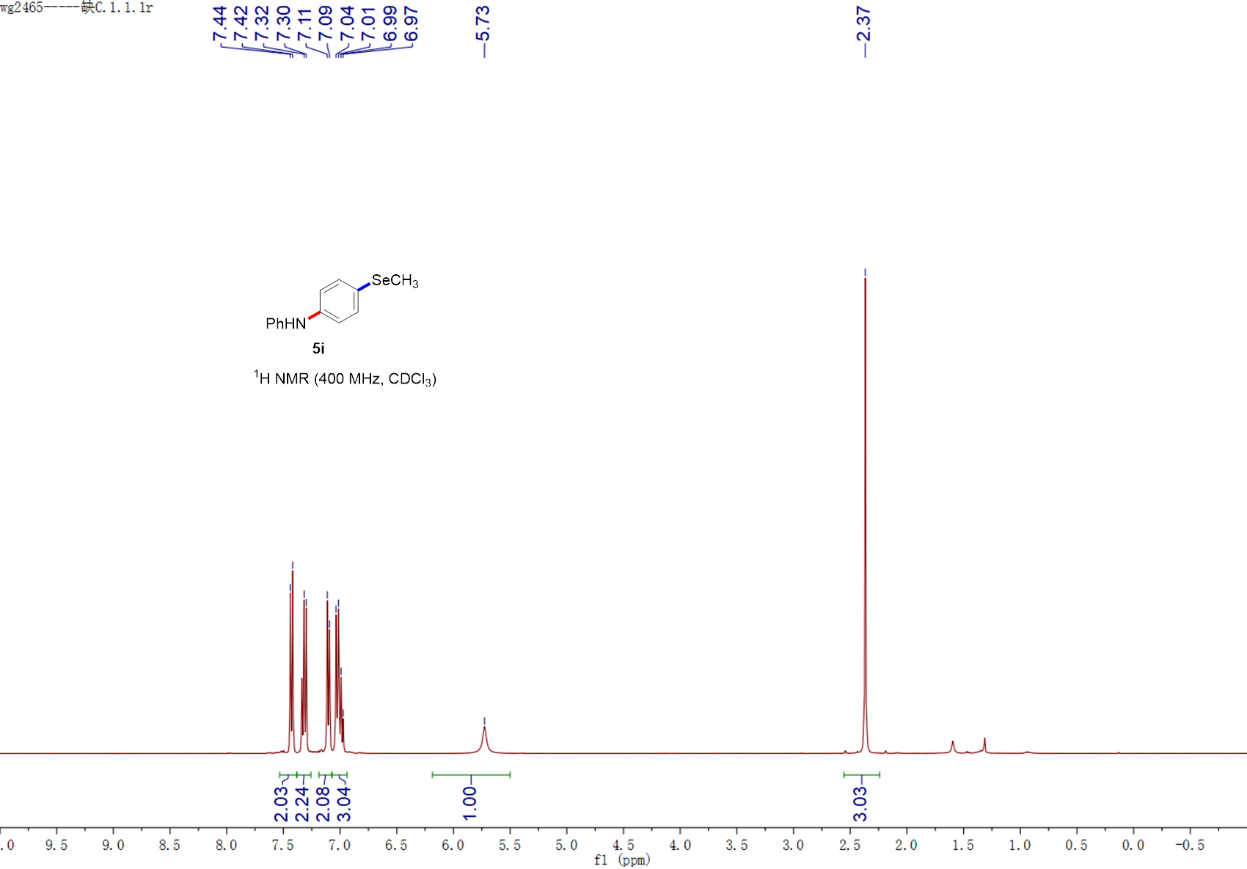
**

**^13^C{^1^H} NMR (100 MHz, Chloroform-*d*) spectrum of 5i**

**
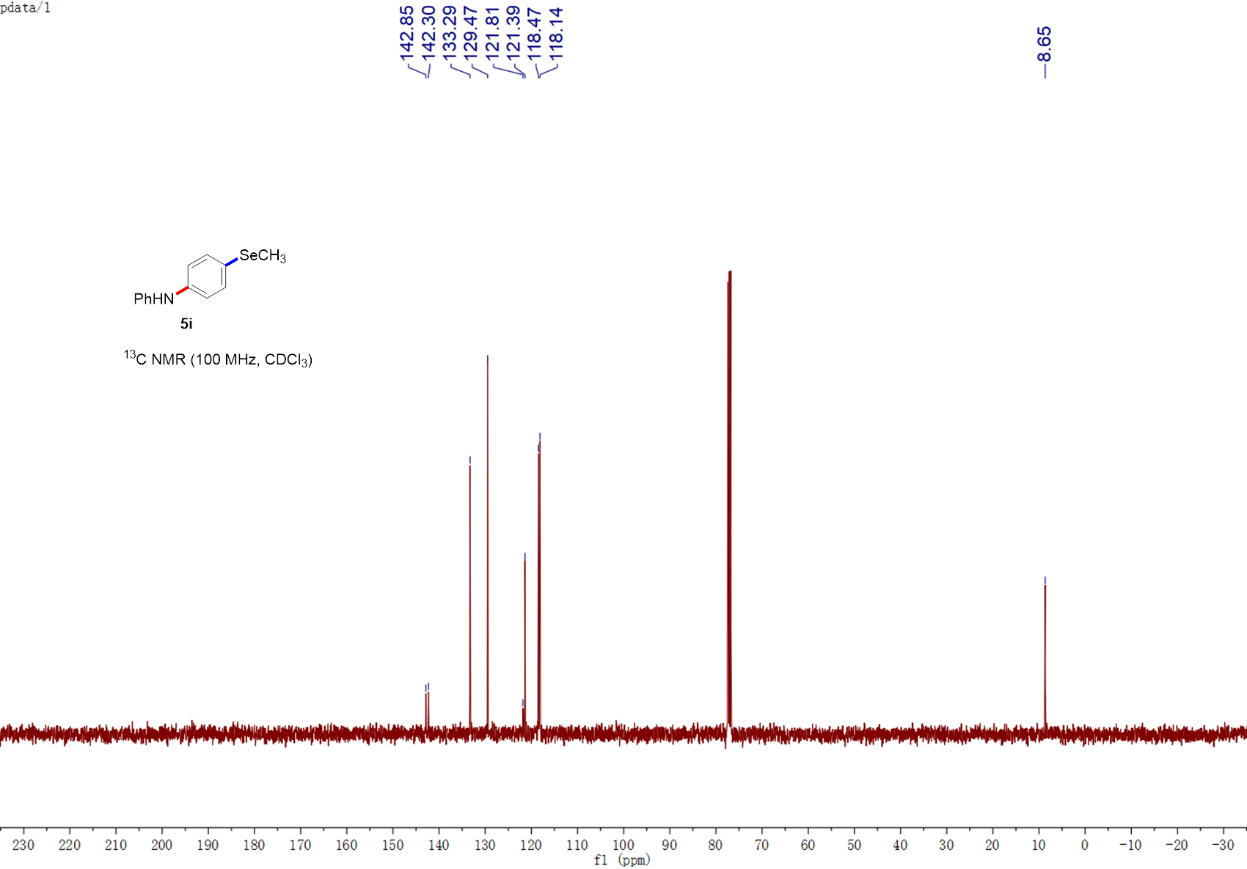
**

**^1^H NMR (400 MHz, Chloroform-*d*) spectrum of 5j**

**^13^C{^1^H} NMR (100 MHz, Chloroform-*d*) spectrum of 5j**

**^1^H NMR (400 MHz, Chloroform-*d*) spectrum of 5k**

**^13^C{^1^H} NMR (100 MHz, Chloroform-*d*) spectrum of 5k**

**HRMS of Products**

**HRMS of 3a**

**HRMS of 3b**

**HRMS of 3c**

**HRMS of 3d**

**HRMS of 3e**

**HRMS of 3f**

**HRMS of 3g**

**HRMS of 3h**

**HRMS of 3i**

**HRMS of 3j**

**HRMS of 3k**

**HRMS of 3l**

**HRMS of 3m**

**HRMS of 3n**

**HRMS of 3o**

**HRMS of 3p**

**HRMS of 3q**

**HRMS of 3r**

**HRMS of 3s**

**HRMS of 3u**

**HRMS of 3v**

**HRMS of 3w**

**HRMS of 3x**

**HRMS of 3y**

**HRMS of 3z**

**HRMS of 3aa**

**HRMS of 3ab**

**HRMS of 4a**

**HRMS of 4b**

**HRMS of 4c**

**HRMS of 4d**

**HRMS of 4e**

**HRMS of 4f**

**HRMS of 4g**

**HRMS of 4h**

**HRMS of 5a**

**HRMS of 5b**

**HRMS of 5c**

**HRMS of 5d**

**HRMS of 5e**

**HRMS of 5f**

**HRMS of 5g**

**HRMS of 5h**

**HRMS of 5i**

**HRMS of 5j**

**HRMS of 5k**
